# Convergent gene losses and pseudogenizations in multiple lineages of stomachless fishes

**DOI:** 10.1038/s42003-024-06103-x

**Published:** 2024-04-03

**Authors:** Akira Kato, Supriya Pipil, Chihiro Ota, Makoto Kusakabe, Taro Watanabe, Ayumi Nagashima, An-Ping Chen, Zinia Islam, Naoko Hayashi, Marty Kwok-Shing Wong, Masayuki Komada, Michael F. Romero, Yoshio Takei

**Affiliations:** 1 School of Life Science and Technology, Tokyo Institute of Technology, Yokohama, Japan; 2 Department of Biological Sciences, Tokyo Institute of Technology, Yokohama, Japan; 3 Center for Biological Resources and Informatics, Tokyo Institute of Technology, Yokohama, Japan; 4 Department of Physiology & Biomedical Engineering, Mayo Clinic College of Medicine & Science, Rochester, MN USA; 5 Department of Marine Bioscience, Atmosphere and Ocean Research Institute, The University of Tokyo, Kashiwa, Japan; 6 Department of Biological Sciences, Faculty of Science, Shizuoka University, Shizuoka, Japan; 7 Department of Biomolecular Science, Toho University, Funabashi, Japan; 8 Cell Biology Center, Institute of Innovative Research, Tokyo Institute of Technology, Yokohama, Japan; 9 Department of Nephrology & Hypertension, Mayo Clinic College of Medicine & Science, Rochester, MN USA

**Keywords:** Evolutionary biology, Molecular evolution, Ichthyology, Stomach

## Abstract

The regressive evolution of independent lineages often results in convergent phenotypes. Several teleost groups display secondary loss of the stomach, and four gastric genes, *atp4a*, *atp4b*, *pgc*, and *pga2* have been co-deleted in agastric (stomachless) fish. Analyses of genotypic convergence among agastric fishes showed that four genes, *slc26a9*, *kcne2*, *cldn18a*, and *vsig1*, were co-deleted or pseudogenized in most agastric fishes of the four major groups. *kcne2* and *vsig1* were also deleted or pseudogenized in the agastric monotreme echidna and platypus, respectively. In the stomachs of sticklebacks, these genes are expressed in gastric gland cells or surface epithelial cells. An ohnolog of *cldn18* was retained in some agastric teleosts but exhibited an increased non-synonymous substitution when compared with gastric species. These results revealed novel convergent gene losses at multiple loci among the four major groups of agastric fish, as well as a single gene loss in the echidna and platypus.

## Abstract (web summary)

Several teleost groups display secondary loss of the stomach. Analyses of genotypic convergence among agastric fishes showed that four genes, *slc26a9*, *kcne2*, *cldn18a*, and *vsig1*, are co-deleted or pseudogenized in most agastric fishes.

## Introduction

Actinopterygii (ray-finned fishes) consists of 44 orders, 453 families, and approximately 30,000 species, thereby constituting the largest class of fishes, as well as greater than half of all extant vertebrates^1–3^. The stomach is absent from the gastrointestinal tract in certain Actinopterygii orders, while others have true stomachs that secrete gastric acid and pepsinogen from the gastric gland. Cypriniformes (~3,200 species; e.g., minnows), Beloniformes and Cyprinodontiformes (~1200 species; e.g., medaka and killifish, respectively), Tetraodontiformes (~3500 species; e.g., pufferfishes), and Labriformes (~600 species; e.g., wrasse) are the main predominantly agastric orders of this class^4,5^. These groups are phylogenetically scattered, showing that the Actinopterygii originally possessed a stomach, but this organ disappeared in the ancestors of each agastric lineage individually. In the most recent review of stomach loss in fishes, Wilson and Castro^5^ estimated that 7% of families and 20–27% of fish species are agastric, and at least 15 individual stomach loss events have occurred in fishes during evolution.

Secondary loss of an organ or tissue is a type of regressive evolution that has received considerable attention as a model of evolution, development, and physiology. These losses are convergent phenotypes, suggesting the presence of a specific benefit and selection in each lineage. For example, secondary eye and pigment losses are observed in cave animals such as cavefishes (*Astyanax mexicanus*, *Amblyopsis rosae*, and *Typhlichthys subterraneus*) and cave salamanders^6,7^, with eye loss suggested to relate to the conservation of metabolic energy^8^. Other examples are the loss of the swim bladder in Pleuronectiformes, Gobiiformes, and Scorpaenidae^9^, and the disappearance of scales in some lineages of Actinopterygii^10^. Most stenohaline marine fishes lack a distal tubule from the nephron of the kidney, and glomeruli are absent in a small number of marine teleosts as they have minimal functional significance^11^. Snakes and scincid lizards have lost their limbs^12,13^, most cetaceans present missing hind limbs^14^, and the aquatic frog *Barbourula kalimantanensis* lacks a lung^15^. In the platypus, the stomach is completely aglandular and has been reduced to a simple dilatation of the lower esophagus^16^. In echidna, the small stomach contains a high gastric fluid pH but lacks a gastric gland^16^. The secondary stomach loss in Actinopterygii, as well as the loss of gastric gland in monotremes, is an interesting example to elucidate the cause of the secondary loss of an organ; nevertheless, the physiological benefits and developmental mechanisms involved in this secondary loss have not yet been clarified.

Genome sequences of many ray-finned fishes have been recently published and the number of species available allows some comprehensive analysis on the genomic difference between gastric and agastric fishes. In particular, agastric fishes from the following four orders of teleosts have been sequenced: zebrafish (*Danio rerio*; Cypriniformes)^17^, Japanese medaka (*Oryzias latipes*; Beloniformes)^18^, pufferfish (*Takifugu rubripes* and *Tetraodon nigroviridis*; Tetraodontiformes)^19,20^, and wrasse (*Labrus bergylta*)^21^. Genome sequences of gastric fishes such as three-spined stickleback (*Gasterosteus aculeatus*)^22^, Atlantic cod (*Gadus morhua*)^23^, and Nile tilapia (*Oreochromis niloticus*)^24^ have also been published. Based on these analyses, the H^+^/K^+^-ATPase (*atp4a* and *atp4b*) and pepsinogens (*pga*, *pgc*) genes are co-deleted in the genomes of agastric species but are present in the genomes of gastric species^4,21^. In monotremes, convergent gene losses for *atp4a*, *atp4b*, *pgc*, and *pga* occurred in platypus, and those for *pgc* and *pga* occurred in echidna, suggesting that the loss of *pgc* and *pga* occurred before the platypus-echidna split at more than 21 mya^16,25^.

During our studies of anion transporters of solute carrier family 26 (Slc26) in pufferfish and eels^26,27^, we found that the gene or cDNA for Slc26a9 was absent in the expressed sequencing tag (EST) and genome databases of pufferfish, zebrafish, and Japanese medaka, but present in those of three-spined stickleback, rainbow trout, Atlantic cod, and Nile tilapia. In mice, Slc26a9 is highly expressed in the stomach and lung^28^, and its deletion causes tubulovesicle loss in parietal cells, acid^29^ and prostaglandin-stimulated HCO_3_^−^ secretion impairment in the stomach^30^, and airway mucus obstruction through airway inflammation^31^. These results indicate that the absence of *slc26a9* in fish species is correlated with stomach loss, and that more genes that are important for gastric function could be lost among agastric fishes in a convergent manner. To confirm this hypothesis, we compared gene losses between agastric and gastric fishes and identified additional genes that are co-deleted in agastric fishes to demonstrate a novel genotypic convergence in relation to stomach loss.

## Results

### Screening of genes co-deleted in the genomes of agastric fishes

**Fig. 1:**
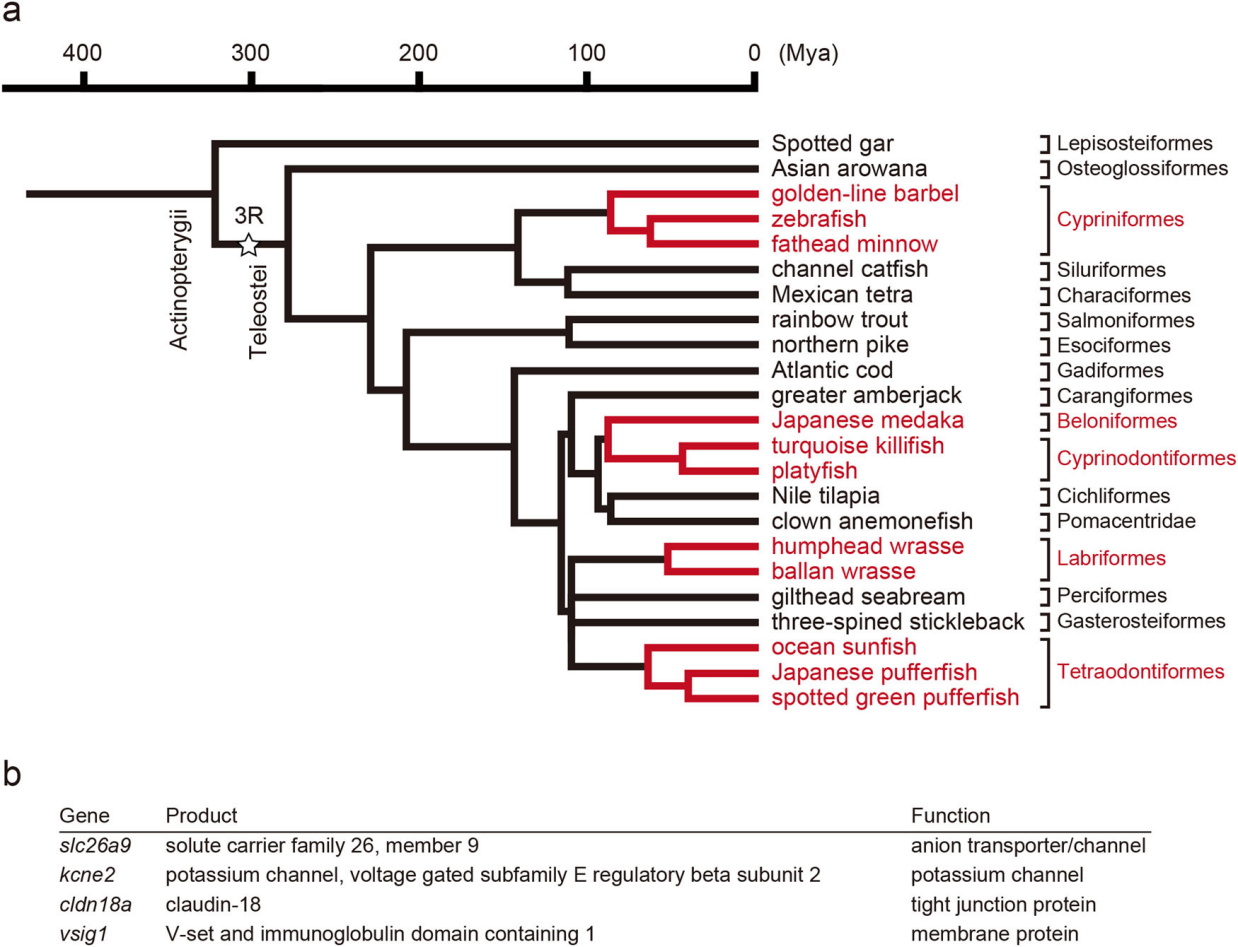
Evolutional relationships of Actinopterygii gastric and agastric species and list of genes co-deleted in the genomes of agastric fishes identified in this study. **a** The time-calibrated phylogeny of 23 species analyzed in this study was prepared based on Near et al. ^1,130^ and the TimeTree database (http://www.timetree.org/)^131^. Species in lineages of the four agastric lineages (Cypriniformes, Beloniformes & Cyprinodontiformes, Tetraodontiformes, and Labriformes) are shown in brown. 3R, teleost-specific third-round whole genome duplication. **b** List of genes co-deleted in the genomes of agastric fishes identified in this study^29,48,49,132^.

Genes which are commonly absent stomachless fish genomes were screened by database mining. First, a list of all annotated genes in the three-spined stickleback genome database^22^ was obtained using the Ensembl BioMart tool^32^ and compared to those of agastric fishes (zebrafish;^17^, Japanese medaka;^18^, spotted green pufferfish;^20^, and Japanese pufferfish;^19^); approximately 80 three-spined stickleback genes were identified that were absent in the gene annotations of the agastric fishes. Second, the presence or absence of the identified genes was confirmed by a homology search in the genome databases for agastric fishes (zebrafish, Japanese medaka, spotted green pufferfish, and Japanese pufferfish). Blast analyses showed that many of those genes were present in agastric fishes but not correctly annotated or annotated with a different name. Ten genes, *atp4a*, *atp4b*, *pgc, slc26a9*, *kcne2*, *vsig1*, *pqlc2l*, *pradc1*, *atp6v0d2*, and *ankub1*, were confirmed to be absent in the genome of these agastric fishes but present in three-spined stickleback. A similar analysis was performed on 23 Actinopterygii species. Phylogenetic relationships among the 23 species are shown in Fig. 1a. Finally, six genes, *atp4a*, *atp4b*, *pgc, slc26a9*, *kcne2*, and *vsig1* were confirmed to be absent in the genome of the majority of the agastric fishes but present in gastric species. Three of these six genes (*atp4a*, *atp4b*, and *pgc*) were also reported absent in agastric fishes by Castro et al.^4^, corroborating the validity of this strategy. However, *pga2* was not included in the list, indicating the incompleteness of this method.

We next individually analyzed the presence of genes whose function or expression in the stomach of mammals was recognized using blast analyses. As previously reported^4^, *pga2* was confirmed to be absent in the genomes of agastric fishes. In addition, a teleost fish-specific ohnolog of the claudin 18 gene, *cldn18a*, was found to be co-deleted in the genome databases of these fishes. Another ohnolog, *cldn18b* was shown to be present in gastric fishes and some agastric fishes (zebrafish and Japanese pufferfish). In total, four genes (*slc26a9*, *kcne2*, *cldn18a*, and *vsig1*) were found to be co-deleted in the genome databases of the most agastric fish species of Actinopterygii (Fig. 1b).

### Identification of genes co-deleted in the genomes of agastric fishes

**Fig. 2:**
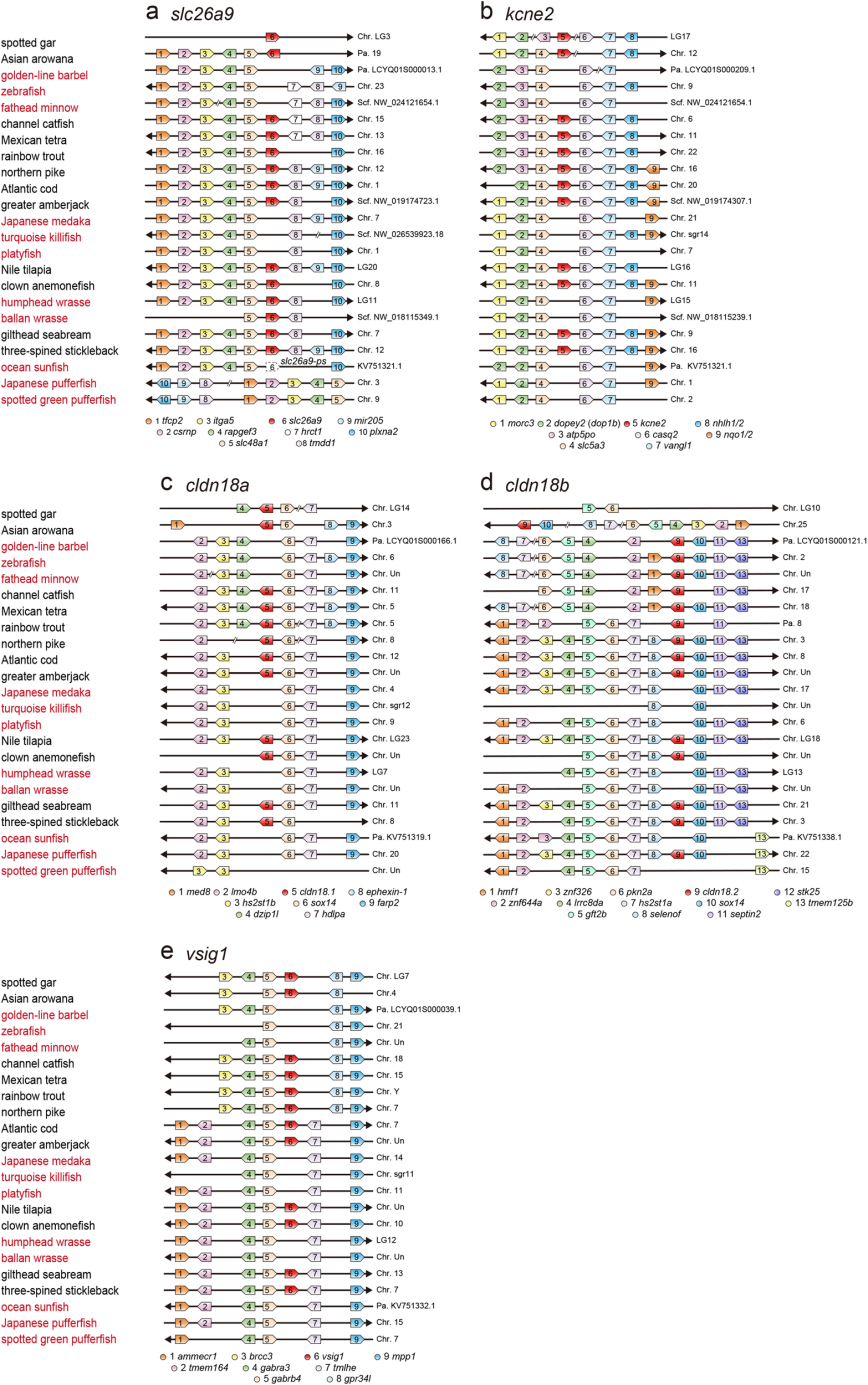
Co-deletion of genes in genomes of agastric fishes found in this study. The synteny analyses of *slc26a9* (**a**), *kcne2* (**b**), *cldn18a* (**c**), *cldn18b* (**d**), *vsig1* (**e**) in the genome databases of 23 Actinopterygii species are shown. Names of species of the four agastric lineages (Cypriniformes, Beloniformes and Cyprinodontiformes, Tetraodontiformes, and Labriformes) are shown in red. Chr. chromosome; Scf. scaffold. Accession numbers of each gene are shown in Supplementary Tables 1–5.

**Fig. 3:**
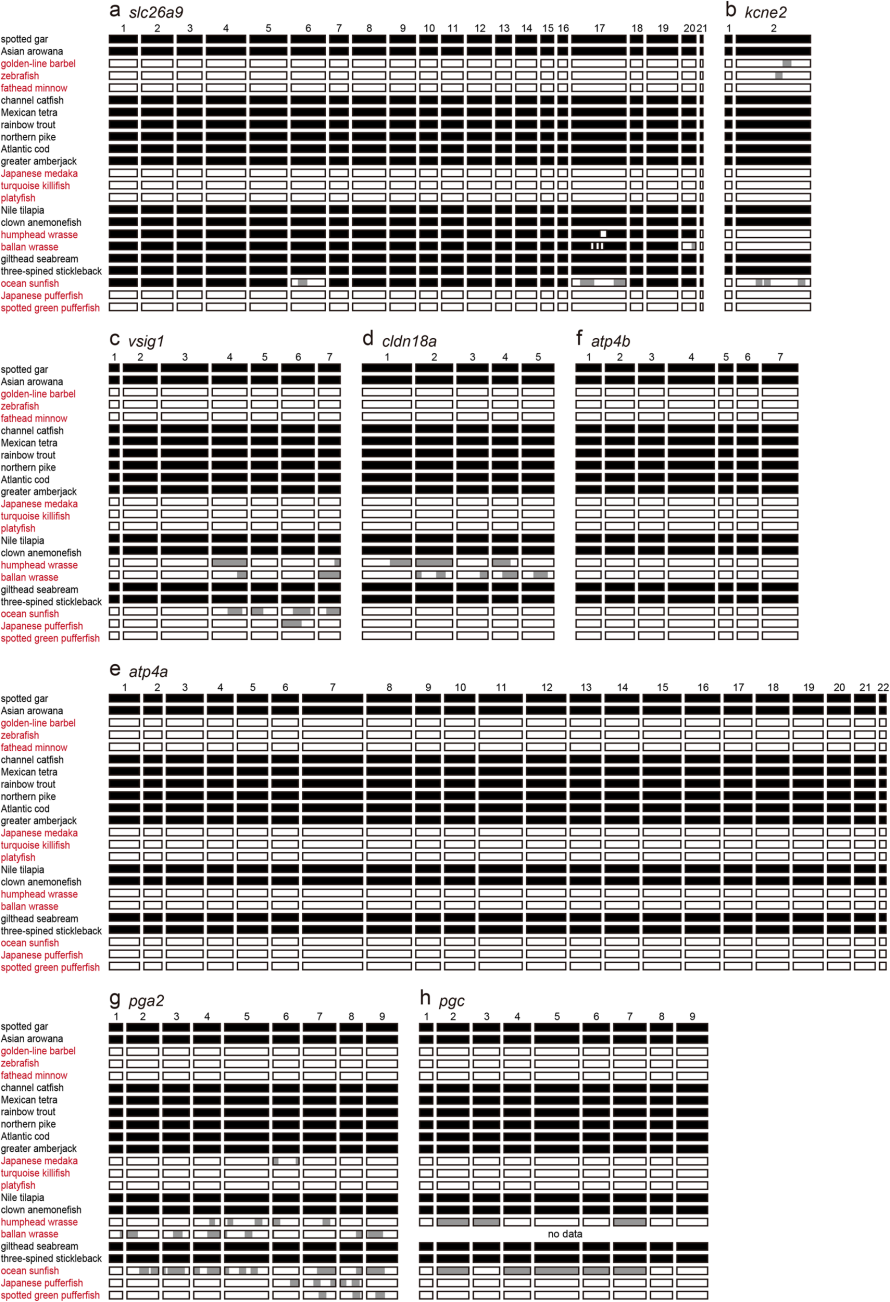
Genetic deletions or changes in agastric fishes. Deletions in the exons of *slc26a9* (**a**), *kcne2* (**b**), *vsig1* (**c**), *cldn18a* (**d**), *atp4a* (**e**), *atp4b* (**f**), *pga2* (**g**), and *pgc* (**h**) in 23 ray-finned fish species. Schematic representations of the dot plot analyses (Supplementary Figs. 1–8) are shown. The presence or absence of exons of each gene are indicated by black and white boxes, respectively. Partially homologous exons are shown by gray boxes. Species in the four agastric lineages are shown in red.

Synteny and dot plot analyses were performed to evaluate gene loss and pseudogenization, respectively. A synteny analysis of the four identified genes (*slc26a9*, *kcne2*, *cldn18a*, and *vsig1*) and the related *cldn18b* ohnolog was performed on 23 Actinopterygii species and shown in Fig. 2 and Supplementary Tables 1–5. The results of dot plot analyses are shown in Supplementary Figs. 1–4. A summary of the presence or deletion of each exon-coding region is shown in Fig. 3. The results showed that *kcne2*, *vsig1*, and *cldn18a* were absent or pseudogenized in all 11 species in four agastric lineages (Cypriniformes, Beloniformes and Cyprinodontiformes, Tetraodontiformes, and Labriformes) but present in all other species in 12 gastric lineages (Figs. 2b, c, e, and 3b–d). *slc26a9* was absent or pseudogenized in nine agastric species in three lineages (Cypriniformes, Beloniformes, Cyprinodontiformes, and Tetraodontiformes) but present in the other species including two species of Labriformes (wrasses) (Figs. 2a and 3a). The *cldn18b* ohnolog was deleted in seven species in two lineages (Beloniformes, Cyprinodontiformes, and Tetraodontiformes) but existed in the other species including four species in agastric lineages (Cypriniformes and Tetraodontiformes) (Fig. 2d).

**Fig. 4:**
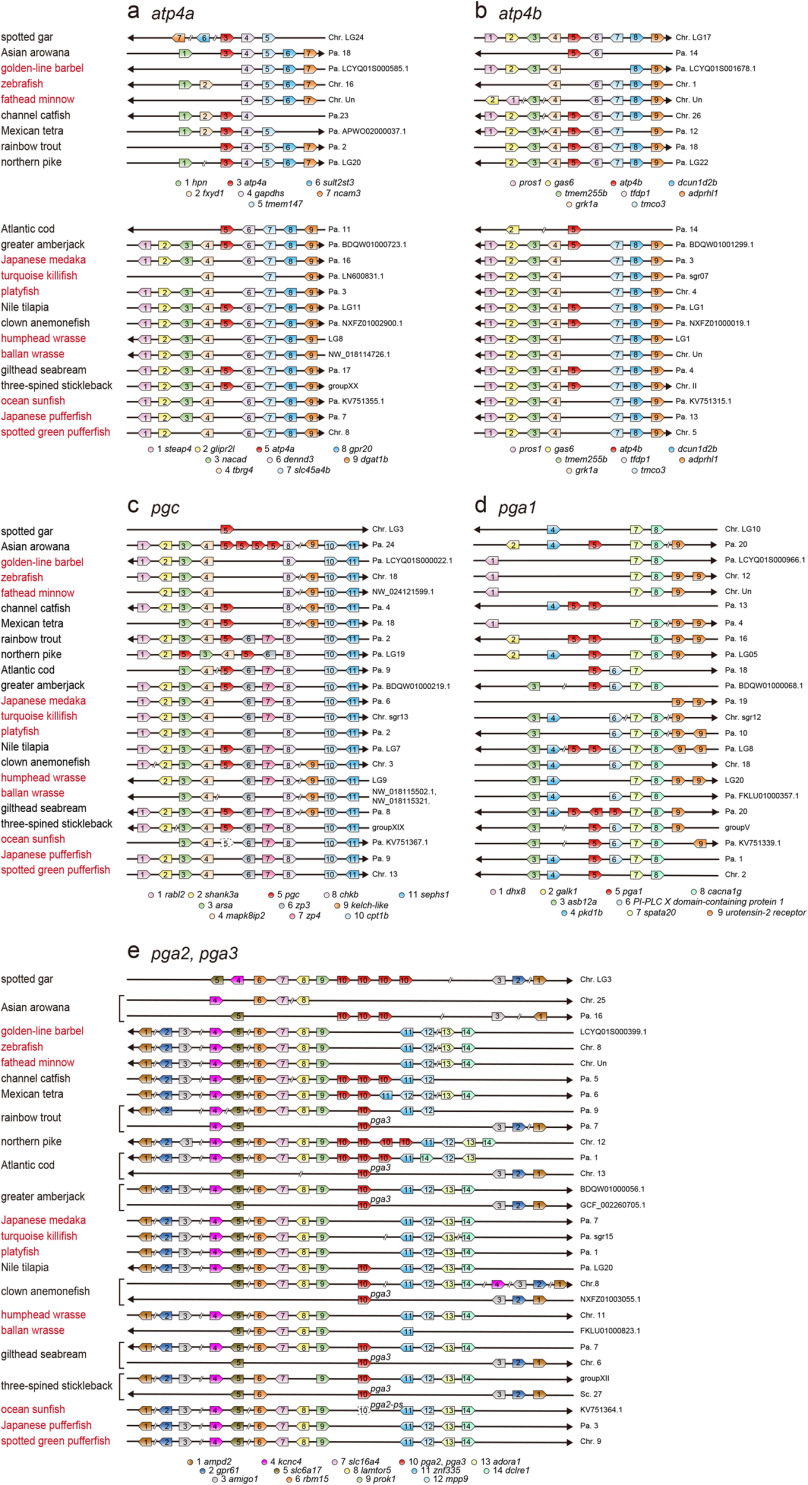
Synteny analyses of genes known to be co-deleted in the genomes of agastric fishes. The synteny analyses of *atp4a* (**a**), *atp4b* (**b**), *pgc* (**c**), and *pga* (**d**, **e**) in the genome databases of 23 Actinopterygii species are shown. Names of species of the four agastric lineages (Cypriniformes, Beloniformes and Cyprinodontiformes, Tetraodontiformes, and Labriformes) are shown in red. Chr., chromosome; Scf., scaffold. Accession numbers of each gene are shown in Supplementary Tables 6–10.

Synteny and dot plot analyses of *atp4a*, *atp4b*, *pgc*, and *pga2* was similarly performed (Figs. 3e–h and 4, Supplementary Figs. 5–8, Supplementary Tables 6–10). The results revealed that *atp4a*, *atp4b*, *pga2*, and *pgc* were absent or pseudogenized in all 11 species in four agastric lineages but present in all the other species in 12 gastric lineages.

**Fig. 5:**
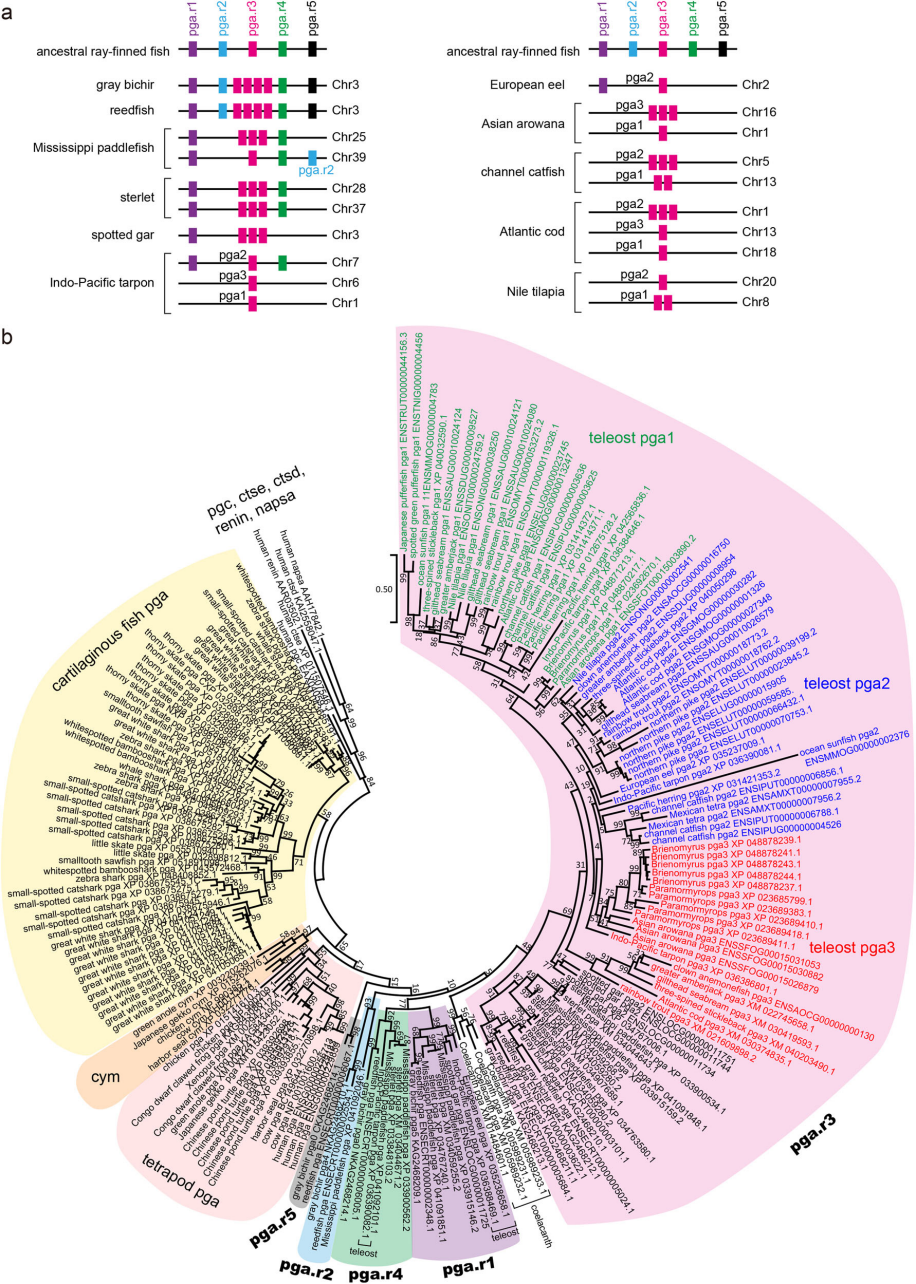
Evolution of *pga* orthologs in ray-finned fishes. **a** Schematic representation of the synteny of ray-finned fish-specific paralogs of *pga*. Putative ancestral paralogs in ray-finned fishes are indicated as *pga.r1*– *pga.r5*. Synteny of *pga* orthologs in representative species are shown. **b** Phylogenetic analysis of *pga* and related genes in ray-finned fishes, coelacanth, tetrapods, and cartilaginous fishes. The deduced amino acid sequence of each gene was aligned using ClustalW software and a phylogenetic tree was constructed using the maximum-likelihood method with MEGA software. Numbers indicate bootstrap values, and the scale bar represents the genetic distance of amino acid substitutions per site. A list of genome databases used for the analysis is shown in Table 1.

*pga* orthologs are distributed in three loci in the teleost genome and are named *pga1*, *pga2*, and *pga3*^4,33^ (Fig. 4d, e). Phylogenetic analysis of *pga* orthologs and the details of the evolutionary relationships are shown in Fig. 5 and described in the next chapter. In contrast to *pga2*, which was deleted in all 11 agastric species, *pga1* was deleted in eight species of three agastric lineages (Cypriniformes, Beloniformes, and Labriformes) but existed in other species including Tetraodontiformes (Fig. 4d). The *pga3* gene was deleted in several agastric and gastric fishes^4^ (Fig. 4e).

### Evolution of *pga* in bony vertebrates

Although teleost fishes have three *pga* paralogs, *pga1*, *pga2*, and *pga3*^4,33^, no clear evolutionary relationship among the paralogs has been uncovered. Therefore, a comprehensive analysis of *pga* was conducted on the genomic data of cartilaginous fish, tetrapods, lobe-finned fish, and ray-finned fish. The *pga* paralog nomenclature in representative species is shown (Fig. 5a), and a molecular phylogenetic tree was constructed (Fig. 5b). These results suggest that cartilaginous fish, tetrapods, lobe-finned fish, and ray-finned fish each have their own *pga* paralogs. In cartilaginous fishes, *pga* paralogs consist of four major branches, indicating that the divergence of these four branches occurred before the speciation of cartilaginous fishes, and that they acquired species-specific paralogs after speciation. The tetrapod *cym* is positioned as a tetrapod-specific paralog. The *pga* paralogs of the coelacanth, a lobe-finned fish, formed a single branch, suggesting that *pga* paralogs evolved independently in lobe-finned fish (Fig. 5b, clear highlight).

The *pga* paralogs of ray-finned fish formed five major branches, each of which contained *pga* from diverse species, suggesting that these five branches arose from the common ancestor of ray-finned fish. In gray bichir and reedfish, all *pga* paralogs were located in tandem (Fig. 5a), suggesting that these ancestral paralogs arose by tandem duplication. In this study, these ancestral *pga* paralogs were provisionally named *pga.r1*, *pga.r2*, *pga.r3*, *pga.r4* and *pga.r5*, with r1-r5 representing paralogs arising from ray-finned fish-specific tandem duplications. Synteny of extant *pga* derived from *pga.r1*-*pga.r5* is shown in Fig. 5a. Gray bichir, for example, has one ortholog derived from *pga.r1*, *pga.r2*, *pga.r4*, and *pga.r5*, and four from *pga.r3*. The spotted gar had one ortholog derived from *pga.r1* and three from *pga.r3*. All previously named *pga1*, *pga2*, and *pga3* in teleost fish are orthologs derived from *pga.r3*. Many teleosts only have orthologs derived from *pga.r3*, whereas the European eel has orthologs derived from *pga.r1* and *pga.r3* and the Indo-Pacific tarpon has orthologs derived from *pga.r1*, *pga.r3*, and *pga.r4*. These results can be considered an example of a birth-and-death model in gene family evolution^34^. In the phylogenetic tree, we included the amino acid sequence derived from the *pga2* pseudogene (*pga2-ps*) of ocean sunfish. Ocean sunfish *pga2-ps* was positioned in the teleost *pga2* group with a long branch.

### Expression of stickleback genes whose orthologs are deleted in agastric fishes

**Fig. 6:**
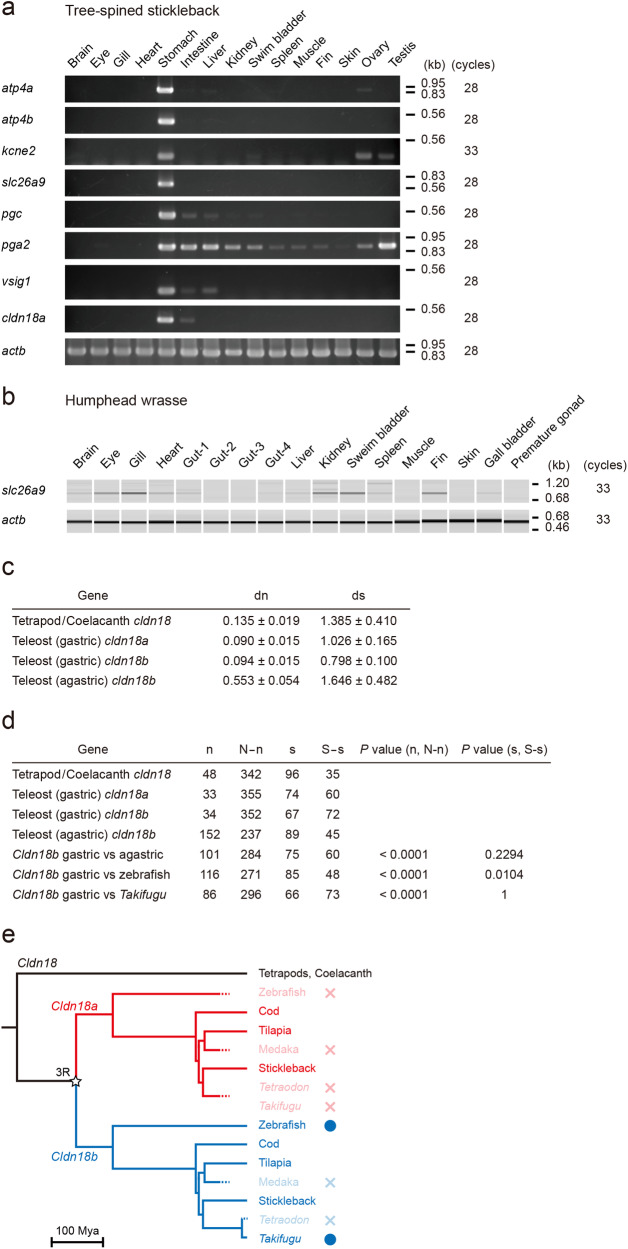
Expression of the three-spined stickleback and humphead wrasse genes whose orthologs are co-deleted in agastric fishes and rapid evolution of *cldn18b* in agastric fishes. **a** Expression of the three-spined stickleback genes whose orthologs are co-deleted in agastric fishes. Reverse transcription-PCR was performed on total RNAs purified from various three-spined stickleback tissues, and analyzed by agarose gel electrophoresis. **b** Expression of humphead wrasse *slc26a9*. Reverse transcription-PCR was performed on total RNAs purified from various humphead wrasse tissues, and the pseudo-gel images of PCR products were generated using a microchip electrophoresis system. *actb* was used as an internal control in each species. Numbers indicate the PCR cycles. **c** Average Jukes-Cantor (JC) distances of claudin 18 coding regions within or among the groups. Variants were estimated using the bootstrap method with 500 replicates. Nucleotide sequences from three species for tetrapod/coelacanth *cldn18*, three species for gastric teleost *cldn18a*, three species for gastric teleost *cldn18b*, and two species for agastric teleost *cldn18b* were used for the analysis. dn, non-synonymous substitutions per site; ds, synonymous substitutions per site. **d** Average numbers of non-synonymous differences (n), synonymous differences (s), unchanged non-synonymous sites (N-n), and unchanged synonymous sites (S-s) of claudin 18 coding regions within or among the groups. *P*-values from two-sided Fisher’s exact test are shown. **e** Evolutionary model of *cldn18* ohnologs in Teleostei. Time-calibrated phylogeny was prepared based on the reports of Near et al.^1,130^ and Kumar and Hedges^133^. Red and blue indicate the two ohnologs, *cldn18a* and *cldn18b*, respectively. Gene losses are indicated by dotted lines in the tree and x marks. Blue circles indicate rapidly evolved genes. An open star indicates teleost-specific third-round whole genome duplication^35^.

Various three-spined stickleback tissues were analyzed by semi-quantitative RT-PCR to determine the distributions of mRNAs for the eight genes (Fig. 6a), as well as for *actb* as a positive control showing cDNA integrity. The results showed that *atp4a*, *atp4b*, *kcne2*, *slc26a9*, *vsig1*, *cldn18a*, *pgc*, and *pga2* were highly expressed in the stomach. Several of these genes were also expressed in stickleback organs other than the stomach: *kcne2* was observed in the ovary and testis, *pgc* in the gut and liver, *pga* in various organs, including the gut, liver, and kidney, *vsig1* in the gut and liver, and *cldn18a* in the gut.

**Fig. 7:**
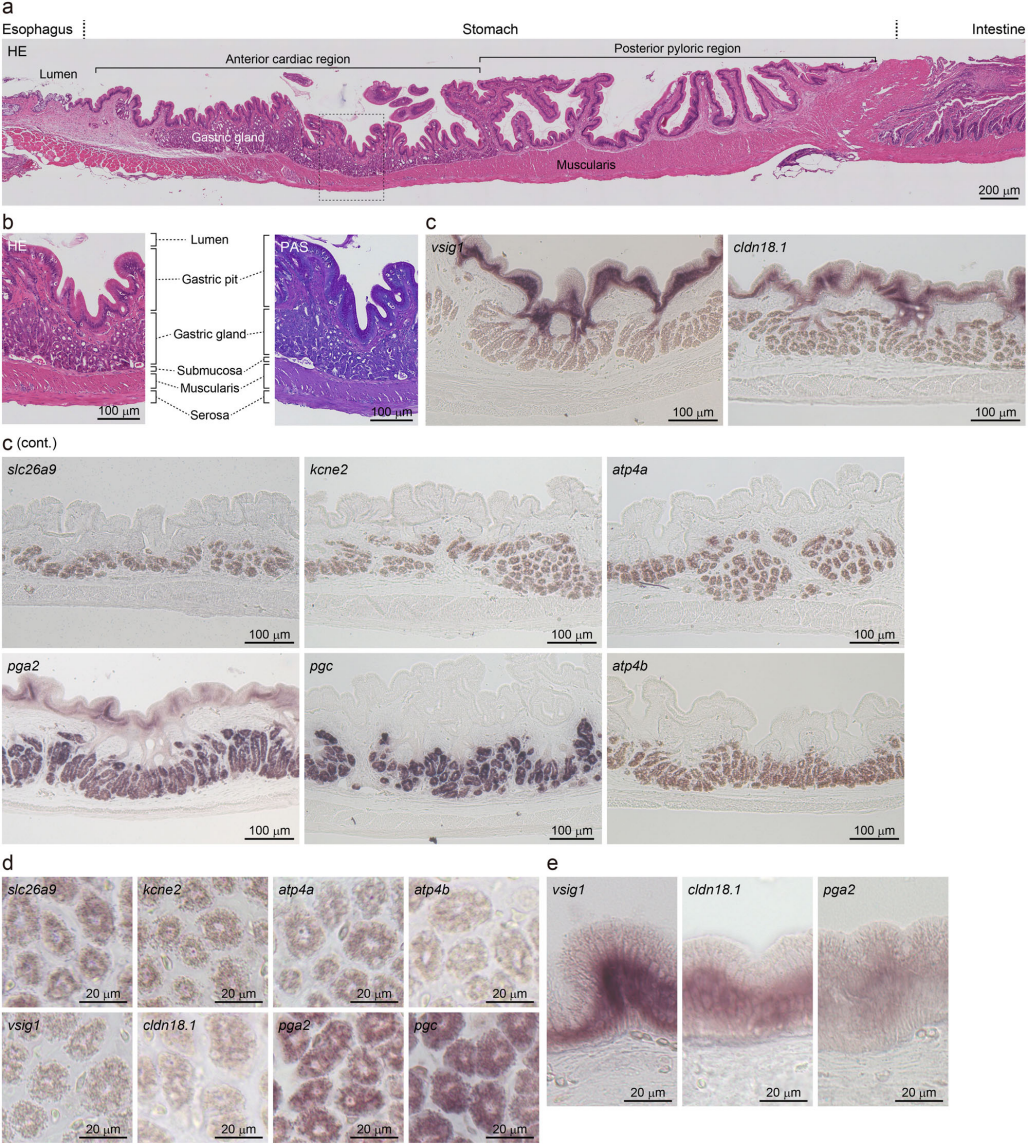
In situ hybridization histochemistry analysis of the three-spined stickleback stomach for the genes whose orthologs are co-deleted in gastric fishes. **a** A vertical section of the whole stomach stained with hematoxylin and eosin. **b** Large magnification views of gastric wall sections stained with hematoxylin and eosin (left) or Periodic acid-Schiff reagent (right). **c**–**e** In situ hybridization. The gastric wall sections were stained with antisense probes. Results with sense probes for the negative control are shown in Supplementary Fig. 1. Large magnification views for the gastric gland and gastric pit are shown in (**d**, **e**), respectively. HE Hematoxylin and eosin, PAS Periodic acid-Schiff.

To identify the cells expressing the genes at the tissue level, in situ hybridization and histology were performed on the three-spined stickleback gut (Fig. 7), which is composed of a mucosa, submucosa, muscularis, and serosa (Fig. 7a, b). The mucosa consists of a gastric pit and gastric (oxyntic) gland in the anterior cardiac or fundic region of the stomach, and a gastric pit only in the posterior pyloric region. All genes tested were expressed in the mucosa of the three-spined stickleback stomach, with none expressed in the other layers. All eight genes, *atp4a*, *atp4b*, *pgc*, *pga2*, *slc26a9*, *kcne2*, *cldn18a*, and *vsig1*, were expressed in gastric gland cells (Fig. 7c, d), and three *pga2*, *cldn18a*, and *vsig1*, were expressed in the columnar mucous cells of the gastric pit (Fig. 7c, e) which had characteristic Periodic acid-Schiff (PAS)-positive mucous granules in the apical region (Fig. 7b). Hybridization using sense probes did not resulted in any labeling (Supplementary Fig. 9). In general, the gastric gland of fishes consists of only one secretory cell type (oxynticopeptic cells), whereas that of mammals is composed of chief cells for digestive-enzyme secretion and parietal cells for acid secretion^5^. In the gastric gland of the three-spined stickleback, most epithelial cells presented positive expressions for genes involved in acid secretion (*atp4a*, *atp4b*, *slc26a9*, *kcne2*) and digestive enzymes (*pgc*, *pga2*) (Fig. 7c, d), indicating that these eight genes are coexpressed in three-spined stickleback oxynticopeptic cells.

### Expression of wrasse *slc26a9*

Intact *slc26a9* was present in wrasses but not in the other agastric species (Figs. 2a and 3a). To confirm whether *slc26a9* is transcribed in organs other than the stomach, total RNA was extracted from various organs of a humphead wrasse and semi-quantitative RT-PCR was performed. In the humphead wrasse, *slc26a9* was expressed in the eyes, gills, fins, and skin (Fig. 6b).

### Rapid evolution of *cldn18b* in agastric fishes

Gastric teleosts have two orthologs for claudin 18, *cldn18a* and *cldn18b*, whereas agastric teleosts have a single or deleted claudin 18 gene. The paralogs are specifically present in Teleostei but not in tetrapods. For both *cldn18a* and *cldn18b* loci, the synteny of the neighboring genes, *hs2st1*/*hs2st1a* and *sox14*, are conserved (Fig. 2c, d). These results indicate that *cldn18a* and *cldn18b* are ohnologs that are generated by teleost-specific genome duplication (TGD)^35^. The presence of *cldn18a* is highly associated with the existence of a stomach, whereas the presence of *cldn18b* is only partially associated with the possession of this organ.

To compare the evolution of *cldn18* between animals with and without a stomach, mean rates for non-synonymous and synonymous substitutions, d_n_ and d_s_, respectively, were calculated for four groups: (i) *cldn18* of tetrapods/coelacanths, (ii) *cldn18a* of gastric fish, (iii) *cldn18b* of gastric fish, and (iv) *cldn18b* of agastric fish (zebrafish and Japanese pufferfish). Non-synonymous substitutions occurred ~4 times more frequently in the *cldn18b* of agastric fishes than in the other groups (*P* < 0.0001; Fisher’s exact probability test) (Fig. 6c–e). These results suggest that the loss of the stomach allows higher amino acid substitution rates on *cldn18b*, which is likely due to the relaxation of functional constraints.

### Pseudogenization of *vsig1* in platypus and loss of *kcne2* in echidna

**Fig. 8:**
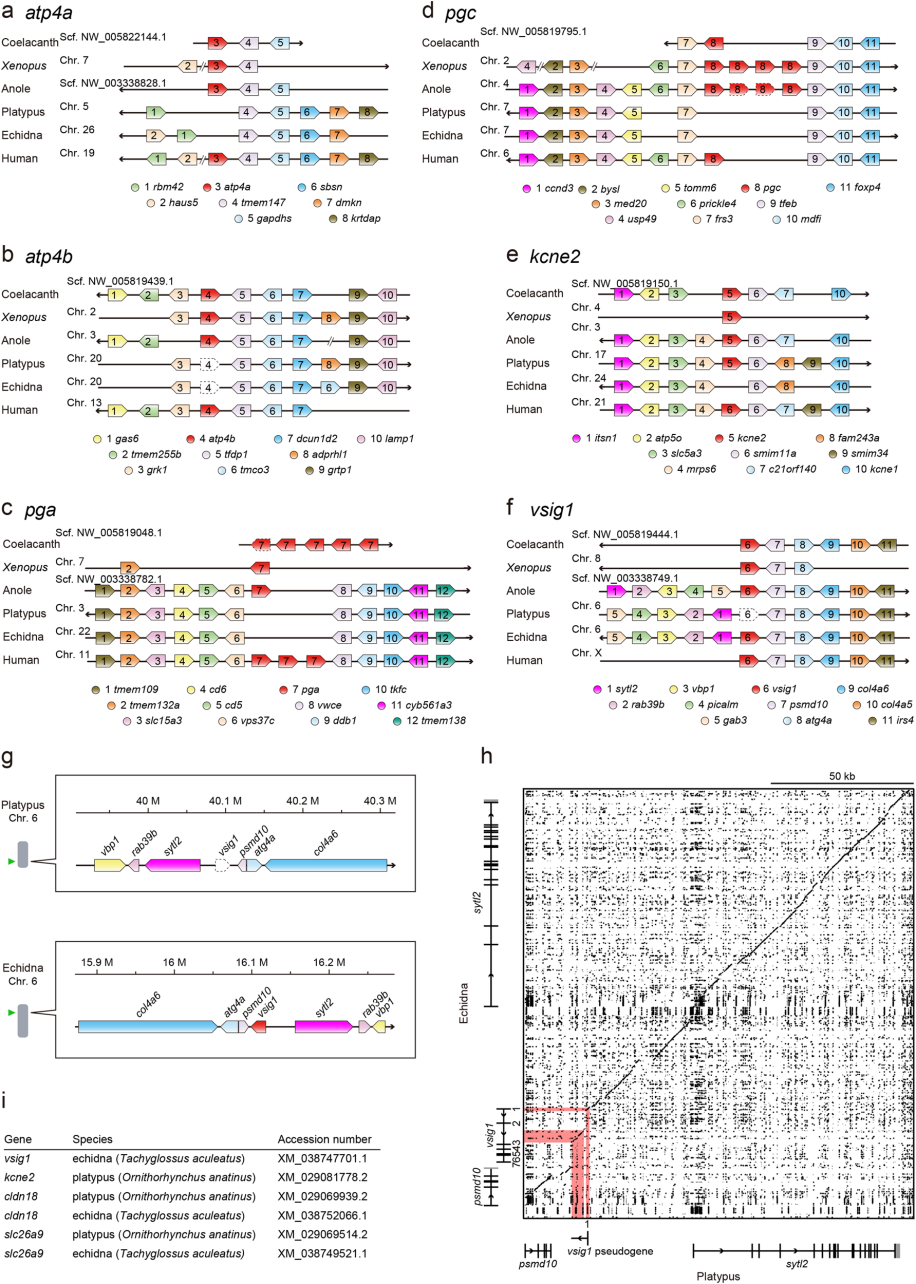
Loss of genes in the genomes of monotremes. The synteny analyses are shown. **a–d** Loss or pseudogenization of *atp4a*, *atp4b*, *pgc*, and *pga2* in platypus and echidna^16,25^. **e** Loss of *kcne2* in echidna but not in platypus. **f** Pseudogenization of *vsig1* in platypus but not in echidna. **a–f** Arrowheads represent the right and left orientations, respectively, of the genome sequences in the NCBI and ENSEMBLE databases. Arrow-shaped boxes indicate the orientation of each gene. Arrow-shaped dotted box indicates pseudogene. Chr., chromosome; Scf., scaffold, Ctg, contig. Accession numbers of each gene are shown in Supplementary Tables 11–16. **g** Chromosomal localization of echidna *vsig1* and platypus *vsig1* pseudogene. **h** Dot plot analysis of the echidna *vsig1* and their flanking regions in comparison with the corresponding genome regions of the platypus containing a *vsig1* pseudogene. Homologous regions were plotted with dotmatcher program (window size: 20; threshold: 70). **i** *vsig1*, *kcne2*, *cldn18*, and *slc26a9* in monotremes.

As reported previously^16,25^, we confirmed convergent gene losses of *atp4a*, *pgc*, and *pga* in the platypus and echidna (Fig. 8a–d). In both organisms, *atp4b* was annotated in the genome database (XM_039915013.1, and XM_038761646.1, respectively); however, the predicted amino acid sequences lacked the amino-terminal cytoplasmic and the transmembrane domains (Supplementary Fig. 10), which are encoded by the exons 1 and 2 of *atp4b* in other species. TBLASTN analysis of the whole-genome databases of platypus and echidna did not reveal regions encoding the cytoplasmic and transmembrane domains of Atp4b. Because Atp4b is a membrane protein with one transmembrane domain^36^, *atp4b* is considered to have lost its original function in the platypus and echidna and may be pseudogenized in these species (Fig. 8b).

The presence or absence of the four genes (*slc26a9*, *kcne2*, *cldn18*, and *vsig1*) was searched using the genome databases of the coelacanth^37^, *Xenopus*^38^, anole lizard^39^, platypus^40^, echidna^25^, and human^41^ by blast searches of their genome sequences. All genes were present in the genomes of the gastric species, coelacanth, *Xenopus*, anole lizard, and human. *cldn18* and *slc26a9* were retained in the genomes of both platypus and echidna (Fig. 8i). Convergent gene loss for *kcne2* was observed in the echidna, but not in the platypus (Fig. 8e). *vsig1* was pseudogenized in the platypus but not in the echidna (Fig. 8f), and dot plot analysis showed a pattern of deletion of *vsig1* in the platypus, with exons 2–7 deleted at the homologous locus of *vsig1* (Fig. 8g–h).

## Discussion

Genome projects of vertebrate species have allowed the clarification of the presence of lineage-specific gene losses during evolution^42–47^. In the present comparative genomic analysis, the deletion of four genes was shown to be associated with secondary stomach losses in Actinopterygii species. The four genes contain the Cl^−^ channel-transporter (*slc26a9*) and a regulatory subunit of the K^+^ channel (*kcne2*). These molecules are co-expressed with H^+^/K^+^-ATPase in gastric gland cells of the stomach and are involved in gastric acid (HCl) secretion. The four genes also contain cell-cell adhesion molecules that are involved in the paracellular barrier function against H^+^ (*cldn18*)^48^ and control the stomach development (*vsig1*)^49^. These results, along with those of other studies on the deletion of genes for H^+^/K^+^-ATPase (*atp4a* and *atp4b*) and pepsinogens (*pga*, *pgc*)^4^, we summarized the convergent losses of important functional genes in four major independent groups of agastric fishes, Cypriniformes (golden-line barbel, zebrafish, and fathead minnow), Beloniformes and Cyprinodontiformes (Japanese medaka, turquoise killifish, and platyfish), Tetraodontiformes (ocean sunfish, Japanese pufferfish and spotted green pufferfish), and Labriformes (humphead wrasse and ballan wrasse). *slc26a9* was present in wrasses and was expressed in organs other than the stomach, such as the gills and skin (Fig. 6b). This result suggests that an unidentified non-gastric function of *slc26a9* prevents its loss from wrasses.

Ocean sunfish (*Mola mola*) belongs to Tetraodontiformes and is closely related to pufferfishes. There is no histological analysis that clarify the presence or absence of gastric glands in the gut of ocean sunfish. In the digestive tract of ocean sunfish, a stomach-like organ is present^50^. However, the present analysis indicates that the genome of ocean sunfish has a similar pattern of gastric gene deletions as pufferfishes and other agastric fishes. This result suggests that the ocean sunfish may be an agastric fish. A stomach-like organ is also present in pufferfishes and is known as the abdominal pouch^51^. The abdominal pouch of pufferfishes is often called stomach and can temporarily store food, but the abdominal pouch does not have gastric glands nor the ability to digest food. In the case for ocean sunfish, further analysis is required to clarify the presence or absence of gastric glands in the stomach-like organ.

**Fig. 9:**
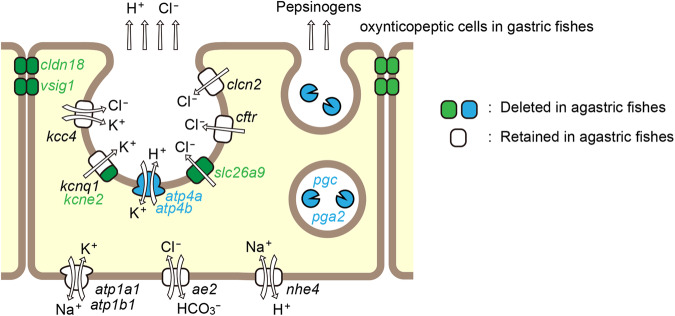
Schematic representation of functions of gastric proteins whose genes are deleted in agastric fishes. Epithelial model for the secretion of gastric acid and digestive enzymes in a fish oxynticopeptic cell is shown. Proteins whose genes were found to be lost in agastric fishes in the previous^4^ or present study are indicated by blue and green, respectively. Gastric proteins that are retained in agastric fishes are illustrated in white.

Gastric H^+^ secretion is mediated by apical (luminal) H^+^/K^+^-ATPase coupled with the K^+^ channel/transporter for K^+^ recycling and is also accompanied by Cl^−^ secretion mediated by the apical Cl^−^ channel/transporter. In mammals, the Cftr, Clc-2, and Slc26a9 Cl^−^ channels are proposed to mediate Cl^−^ secretion^52,53^. K^+^ is recycled by a K^+^ channel composed of Kcnq1 α and Kcne2 β subunits. In addition, the apical K^+^-Cl^−^ cotransporter (Kcc4) secretes K^+^ and Cl^−^ together. Among the apical components for gastric acid secretion, four genes, *atp4a*, *atp4b*, *slc26a9*, and *kcne2* are deleted in agastric fishes, suggesting that the function of those genes is closely associated with gastric acid (H^+^) secretion. The remaining genes were retained in agastric fishes, suggesting that they have important functions in non-gastric tissues of the agastric fishes. In non-gastric tissues, Cftr excretes Cl^−^ in the gills of marine teleosts and secretes intestinal Cl^− ^^54–56^. Kcc4 is involved in H^+^ secretion in the renal α-intercalated cells in mammals^57^, which may explain why these genes are retained. The lost genes code for some of the apical components but not the basolateral components such as Na^+^/K^+^-ATPase, anion exchanger 2 (Ae2), and Na^+^/H^+^ exchanger 4 (Nhe4) for gastric acid secretion (Fig. 9). In general, the basolateral membrane of epithelial cells faces the extracellular fluid with a stable ionic composition, whereas the apical membrane faces the luminal fluid with a variable composition. Therefore, functional proteins on the apical membrane tend to be tissue-specific, while those on basolateral membrane are shared among epithelia of various tissues. Our results suggest that the basolateral components for gastric acid secretion are common with those of other epithelial systems, thereby preventing the deletion of these genes, whereas some apical components are specific to the stomach, which are more prone to gene losses.

Our analysis revealed that the platypus genome contains *kcne2*, *slc26a9*, and *cldn18*, whereas the echidna genome contains *vsig1*, *slc26a9*, and *cldn18*. In mammals, *kcne2*^58,59^, *slc26a9* ^28,29,60,61^, and *cldn18*^62,63^ are expressed in the lung at high levels as well as in the stomach and their functions are related to both gastric and pulmonary systems. Slc26a9 is critical for respiratory function in terrestrial vertebrates as loss of *slc26a9* can create a cystic fibrosis-like phenotype^64–66^. In contrast in the three-spined stickleback, these genes are expressed in the stomach but not in the swim bladder or gill, which are related to respiratory function. These results suggest that *kcne2*, *slc26a9*, and *cldn18* are required mainly for gastric function in Actinopterygii, with the exception of wrasse slc26a9, which has non-gastric functions, whereas those are required for the gastric and pulmonary functions both in terrestrial vertebrates. Therefore, in platypus, the respiratory function of *slc26a9*, *kcne2*, and *cldn18* in the lung may prevent the loss of these genes. In echidnas, the respiratory function of *slc26a9* and *cldn18* in the lung may also prevent the loss of these genes. However, *kcne2* was lost in the echidna, suggesting that the respiratory function of *kcne2* was compensated for by another gene in this organism. RT-PCR analysis of sticklebacks showed that *kcne2*, *pga*, *pgc*, *pga*, *vsig1*, and *cldn18a* were expressed not only in the stomach but also in other organs. This result suggests that these genes function in organs other than the stomach of fish. However, in most agastric fish, these genes were deleted, probably because these functions were compensated for by another gene.

The loss of *vsig1* was observed in agastric fishes and platypus, but not in echidna. Vsig1 is a cell surface protein characterized by two extracellular immunoglobulin-like domains whose physiological function is still largely unknown. Vsig1 is also known as glycoprotein A34 (Gpa34) of tumor cells^67^, is expressed in low- or non-metastatic cancer cells^68^, and inhibits Yap/Taz signaling. Yap and Taz are transcriptional regulators and essential for cancer initiation or growth of most solid tumors^69^. As the Yap/Taz signaling is important for organogenesis^70^, the role of Vsig1 for normal stomach development could be via the TAP/TAZ signaling^49^. In human and mice, the *vsig1* gene is expressed in the stomach and testes^49,67^, while it was expressed in the stomach, intestine, and liver, but not in testes or other organs in the three-spined stickleback (Fig. 6a). Our result also indicated that Vsig1 is localized at the gastric gland and pit cells, which is identical to the case in mice^49^. The loss of *vsig1* could impair the development of stomach in platypus. Although the *vsig1* is an intact gene in echidna, their stomach is glandless. In this case, Vsig1 could be involved in the development of the stomach but some other factors control the development of the gastric gland.

Retention of *cldn18b*, a duplicated *cldn18* in teleosts, by some agastric fishes is a good example of how a gene evolves when the functional constraint is reduced. In the gastric epithelium, paracellular H^+^ leakage is prevented by the tight junctions and associated junctional complexes, e.g., claudins. Only one component, claudin-18, has been identified as the paracellular H^+^ barrier^48^. Complete deletion of both *cldn18a* and *cldn18b* in the genomes of Japanese medaka, turquoise killifish, platyfish, wrasses, ocean sunfish, and spotted green pufferfish indicates that the secondary stomach loss reduced the functional constraint of the *cldn18* genes. The *cldn18b* that is retained in some other agastric species (golden-line barbel, zebrafish, fathead minnow, and Japanese pufferfish) exhibited rapid non-synonymous substitution rates, which were higher than those of gastric species. Although *cldn18b* is retained in Japanese pufferfish, no tissues expressed the gene^71^. However, in zebrafish, *cldn18b* is also expressed in the kidney^72^. In mouse kidney, *cldn18* is expressed in the thick ascending limb of Henle’s loop (TAL), which additionally expresses *cldn10*, *cldn16*, and *cldn19*. The mouse TAL functions as a site for the reabsorption of Ca^2+^ and Mg^2+^ via the paracellular pathway. In the mouse TAL, claudin-10 (claudin-10a: anion permeability; claudin-10b: cation (Na^+^ > K^+^) permeability) and -18 may contribute to the maintenance of barrier function, and claudin-16 and -19 contribute to Ca^2+^ and Mg^2+^ ion selectivity^73–75^. Because zebrafish kidneys also expresses claudin-10b^72^, zebrafish claudin-18, together with claudin-10b and others, may contribute to the maintenance of tubular barrier function.

Many vertebrates have multiple *pga* gene paralogs. It is difficult to evaluate the evolutionary relationships of paralogs using the names of genes, as they are a mixture of those arising from old and new gene duplications. Castro et al. named *pga1*, *pga2*, and *pga3* as *pga* paralogs in three loci of the teleost genome^4^. Molecular phylogenetic analyses involving *pga* genes in cartilaginous fish, tetrapods, lobe-finned fish, and ray-finned fish have shown that teleost *pga1*, *pga2*, and *pga3* differ from *pga* paralogs in ancient ray-finned fishes, such as Polypterus, sturgeon, and gar. This confirmed that *pga1*, *pga2*, and *pga3* are teleost-specific paralogs, as reported by Castro et al.^4^. Interestingly, of the four *pga* paralogs in spotted gar (provisionally named *Locpga1*, *Locpga2*, *Locpga3*, and *Locpga4*), *Locpga1* belonged to the same branch as the *pga* paralogs of polypterus and sturgeons; *Locpga2*, *Locpga3*, and *Locpga4* belonged to the same branch as teleosts *pga1*, *pga2*, and *pga3*, which are paralogs that arose after the divergence of gar and teleosts. Synteny analysis suggested that *pga2* and *pga3* are present in loci generated by teleost-specific genome duplication (TGD); however, it remains unclear whether *pga2* and *pga3* are ohnologs or paralogs derived from pre-TGD tandem duplication. Species- and lineage-specific tandem duplications of *pga2* have been observed in various species (e.g., channel catfish, Mexican tetra, northern pike, and Atlantic cod). In the present analysis, *pga2* was the *pga* family member whose absence was most frequently associated with secondary loss of the stomach, whereas *pga1* and *pga3* were also observed in various gastric fishes. *pga1* synteny was conserved in many teleost species, although no synteny was observed with teleost *pga2*, *pga3*, or tetrapod *pga*. Given this, and the fact that *pga1* is a teleost-specific paralog, it is possible that *pga1* arose in the common ancestor of teleost fish via duplication through translocation. Among teleost fishes, *pga1* was present in most gastric fishes and some agastric fishes and was absent in some gastric fishes and many agastric fishes. In the agastric Japanese pufferfish, *pga1* is expressed in non-gastric tissues such as the skin^76^.

The physiological advantages of secondary stomach loss are still largely unknown^5^. In the treatment of human gastric cancer, gastrectomy alters physiological properties such as oxygen availability, pH, food transit time, intestinal motility, and hormonal conditions, and alters the overall microbiome community structure^77^. Gastrectomy-associated alterations in microbial functions, such as nutrient transport and biosynthesis of organic compounds, may be related to changes in post-gastrectomy metabolism. In gastric teleost species, the stomach has a variety of physiological functions, such as food digestion, temporal food storage, pathogen invasion defense, and hormonal secretion^5^. The differences in the physiological properties between gastric and agastric fish remain unclear. As the stomach kills microorganisms using gastric acid and provides increased uniformity in the population of gut microbes^78^, it is presumed that loss of the secondary stomach has some effect on the gut microbiome, and that the gut microbiome of agastric fish is more susceptible to environmental influences. Studies on the fish digestive tract microbiome indicate that fish harbor specialized gastrointestinal microbial communities like other vertebrates such as mammals^79–81^, and the gut microbiomes of wood-eating catfishes, zebrafish, guppies, and others are related to their diets^79,82–85^. Further studies are required to better understand the physiological advantages of losing the secondary stomach.

Our results raise the question of whether the gene deletions observed in this study caused the stomach loss, or whether the deletions occurred after the stomach loss. Despite stomach loss, our study did not show deletion of the genes for transcriptional or growth factors that regulate stomach development in agastric fishes^86–88^. Thus, it is conceivable that the lack of a stomach is associated with the malfunction of the cis-regulatory elements for stomach development, which cannot be identified using the current strategy. It is also possible that a deletion of one of the eight genes caused a depletion of stomach function in fishes for which this depletion was neutral or advantageous, and additional gene deletion followed, causing the stomach to be completely regressed in the gut of fishes.

In conclusion, we identified novel genes that were lost in agastric fishes among four major teleost lineages, which suggests a convergent evolution scenario in relation to stomach loss. These genes encode apical ion channels for gastric acid secretion, and the cell-cell adhesion molecule that forms the paracellular H^+^ barrier in the gastric epithelium (Fig. 9). These results indicate that a common cassette of gene losses occurred independently during or after stomach loss in the several agastric fish groups. Further studies are required to identify the causative genotype that triggered this stomach loss.

## Methods

### Screening of genes co-deleted in the genomes of agastric fishes

Lists of all annotated genes in the genome databases for zebrafish (*Danio rerio*)^17^, Atlantic cod (*Gadus morhua*)^23^, Nile tilapia (*Oreochromis niloticus*)^24^, Japanese medaka (*Oryzias latipes*)^18^, three-spined stickleback (*Gasterosteus aculeatus*)^22^, Japanese pufferfish (*Takifugu rubripes*)^19^, and spotted green pufferfish (*Tetraodon nigroviridis*)^20^ were downloaded from Ensembl (http://www.ensembl.org/index.html)^89^ using Ensembl BioMart tool^32^. After removing characters that indicated gene duplications, the presence or absence of all annotated three-spined stickleback genes in agastric fishes (zebrafish, Japanese medaka, spotted green pufferfish, and Japanese pufferfish) were determined through a text search using Excel software (Microsoft, Redmond, WA, USA). From this data, a list of three-spined stickleback genes that were commonly lacking in the gene lists of zebrafish, Japanese medaka, spotted green pufferfish, and Japanese pufferfish was prepared. To avoid the presence of annotated genes with different gene names or unannotated genes in the agastric genome data, the absence of the genes was confirmed using a BLAST search (TBLASTN)^90^ of zebrafish, Japanese medaka, spotted green pufferfish, and Japanese pufferfish with Ensembl, and gene names with one or more orthologs were removed from the list. The presence of the orthologs of the listed genes for jawed vertebrate species listed Table 1 were analyzed by text search or TBLASTN analyses using Ensembl and NCBI. The synteny of each gene in the list was compared among the above species using Ensembl and NCBI.

**Table 1:** Genome databases used for synteny analysis of gastric and agastric Actinopterygii species and the evolutionary analysis of *pga* in vertebrates

Category	Species	Genome database	Remarks
ray-finned fish	spotted gar (*Lepisosteus oculatus*)	GCF_000242695.1^100^	synteny analysis, evolution of *pga*
ray-finned fish	Asian arowana (*Scleropages formosus*)	GCF_900964775.1^101^	synteny analysis, evolution of *pga*
ray-finned fish	golden-line barbell (*Sinocyclocheilus grahami*)	GCF_001515645.1^102^	synteny analysis
ray-finned fish	zebrafish (*Danio rerio*)	GCF_000002035.6^17^	synteny analysis
ray-finned fish	fathead minnow (*Pimephales promelas*)	GCF_016745375.1^103^	synteny analysis
ray-finned fish	channel catfish (*Ictalurus punctatus*)	GCF_001660625.3^104^	synteny analysis, evolution of *pga*
ray-finned fish	Mexican tetra (*Astyanax mexicanus*)	GCF_023375975.1^105^	synteny analysis, evolution of *pga*
ray-finned fish	rainbow trout (*Oncorhynchus mykiss*)	GCF_013265735.2^106^	synteny analysis, evolution of *pga*
ray-finned fish	northern pike (*Esox lucius*)	GCF_011004845.1^107^	synteny analysis, evolution of *pga*
ray-finned fish	Atlantic cod (*Gadus morhua*)	GCF_902167405.1^23^	synteny analysis, evolution of *pga*
ray-finned fish	greater amberjack (*Seriola dumerili*)	GCF_002260705.1^108^	synteny analysis, evolution of *pga*
ray-finned fish	Japanese medaka (*Oryzias latipes*)	GCF_002234675.1^18^	synteny analysis
ray-finned fish	turquoise killifish (*Nothobranchius furzeri*)	GCF_027789165.1^109^	synteny analysis
ray-finned fish	platyfish (*Xiphophorus maculatus*)	GCF_002775205.1^110^	synteny analysis
ray-finned fish	Nile tilapia (*Oreochromis niloticu*s)	GCF_001858045.2^24^	synteny analysis, evolution of *pga*
ray-finned fish	clown anemonefish (*Amphiprion ocellaris*)	GCF_022539595.1^111^	synteny analysis, evolution of *pga*
ray-finned fish	humphead wrasse (*Cheilinus undulatus*)	GCF_018320785.1^112^	synteny analysis
ray-finned fish	ballan wrasse (*Labrus bergylta*)	GCF_900080235.1^21^	synteny analysis
ray-finned fish	gilthead seabream (*Sparus aurata*)	GCF_900880675.1^113^	synteny analysis, evolution of *pga*
ray-finned fish	three-spined stickleback (*Gasterosteus aculeatus*)	GCF_016920845.1^22^	synteny analysis, evolution of *pga*
ray-finned fish	ocean sunfish (*Mola mola*)	GCA_001698575.1^114^	synteny analysis, evolution of *pga*
ray-finned fish	Japanese pufferfish (*Takifugu rubripes*)	GCF_901000725.2^19^	synteny analysis, evolution of *pga*
ray-finned fish	spotted green pufferfish (*Tetraodon nigroviridis*)	GCA_000180735.1^20^	synteny analysis, evolution of *pga*
lobe-finned fish	coelacanth (*Latimeria chalumnae*)	GCF_000225785.1^37^	synteny analysis, evolution of *pga*
tetrapod	tropical clawed frog (*Xenopus tropicalis*)	GCF_000004195.4^38^	synteny analysis, evolution of *pga*
tetrapod	anole lizard (*Anolis carolinensis*)	GCF_000090745.2^39^	synteny analysis, evolution of *pga*
tetrapod	platypus (*Ornithorhynchus anatinus*)	GCF_004115215.2^40^	synteny analysis
tetrapod	echidna (*Tachyglossus aculeatus*)	GCF_015852505.1^25^	synteny analysis
tetrapod	human (Homo sapiens)	GCF_000001405.40^41^	synteny analysis, evolution of *pga*
ray-finned fish	reedfish (*Erpetoichthys calabaricus*)	GCF_900747795.2	evolution of *pga*
ray-finned fish	gray bichir (*Polypterus senegalus*)	GCF_016835505.1^115^	evolution of *pga*
ray-finned fish	sterlet (*Acipenser ruthenus*)	GCF_902713425.1^116^	evolution of *pga*
ray-finned fish	Mississippi paddlefish (*Polyodon spathula*)	GCF_017654505.1^117^	evolution of *pga*
ray-finned fish	*Brienomyrus brachyistius*	GCF_023856365.1^118^	evolution of *pga*
ray-finned fish	*Paramormyrops kingsleyae*	GCF_002872115.1^119^	evolution of *pga*
ray-finned fish	Indo-pacific tarpon (*Megalops cyprinoides*)	GCF_013368585.1	evolution of *pga*
ray-finned fish	European eel (*Anguilla anguilla*)	GCF_013347855.1^120^	evolution of *pga*
ray-finned fish	Atlantic herring (*Clupea harengus*)	GCF_900700415.2^121^	evolution of *pga*
tetrapod	cow (*Bos taurus*)	GCF_002263795.3^122^	evolution of *pga*
tetrapod	chicken (*Gallus gallus*)	GCF_016699485.2^123^	evolution of *pga*
tetrapod	Japanese gecko (*Gekko japonicus*)	GCF_001447785.1^124^	evolution of *pga*
tetrapod	Reeves’s turtle (*Mauremys reevesii*)	GCF_016161935.1^125^	evolution of *pga*
tetrapod	Congo dwarf clawed frog (*Hymenochirus boettgeri*)	GCA_019447015.1	evolution of *pga*
cartilaginous fish	great white shark (*Carcharodon carcharias*)	GCF_017639515.1^126^	evolution of *pga*
cartilaginous fish	whale shark (*Rhincodon typus*)	GCF_021869965.1^127^	evolution of *pga*
cartilaginous fish	zebra shark (*Stegostoma tigrinum*)	GCF_030684315.1^127^	evolution of *pga*
cartilaginous fish	whitespotted bambooshark (*Chiloscyllium plagiosum*)	GCF_004010195.1^128^	evolution of *pga*
cartilaginous fish	smaller spotted catshark (*Scyliorhinus canicula*)	GCF_902713615.1	evolution of *pga*
cartilaginous fish	thorny skate (*Amblyraja radiata*)	GCF_010909765.2	evolution of *pga*
cartilaginous fish	smalltooth sawfish (*Pristis pectinata*)	GCF_009764475.1	evolution of *pga*
cartilaginous fish	little skate (*Leucoraja erinacea*)	GCF_028641065.1^129^	evolution of *pga*

### Dot plot analysis

To analyze the pseudogenization or whole gene deletion of the eight genes *slc26a9*, *kcne2*, *vsig1*, *cldn18a*, *atp4a*, *atp4b*, *pga2*, and *pgc*, in the 11 agastric fish species, the coding region of each gene and its flanking regions of the gastric species, three-spined stickleback (*Gasterosteus aculeatus*), and channel catfish (*Ictalurus punctatus*) were compared with the corresponding genomic regions of the 11 agastric fish species listed in Fig. 1. Dot plot comparisons were performed using the EMBOSS dotmatcher program with a window size of 20 and threshold score of 70 (https://www.ebi.ac.uk/Tools/emboss/). To analyze the pseudogenization of platypus *vsig1*, a dot plot analysis was performed between echidna *vsig1* and its flanking regions and the corresponding genome regions of the platypus containing the *vsig1* pseudogene using the EMBOSS dotmatcher program with a window size of 20 and a threshold score of 70.

### Phylogenetic and synteny analyses of *pga*

*pga* orthologs were identified in the genome data of ray-finned fish, lobe-finned fish, tetrapods, and cartilaginous fish, as listed in Table 1. The deduced amino acid sequences were aligned using ClustalW software, and a phylogenetic tree was constructed using MEGA11^91^ using the maximum likelihood method. The synteny of *pga* was compared among the above species using the Ensembl and NCBI databases.

### Semi-quantitative reverse transcription (RT)-PCR

Three-spined sticklebacks (*Gasterosteus aculeatus*) and humphead wrasses (*Cheilinus undulatus*) captured in Japan in 2012 and 2023, respectively, were obtained from local dealers. The animal protocols were in accordance with a manual approved by the Institutional Animal Experiment Committee of the Tokyo Institute of Technology. We have complied with all relevant ethical regulations for animal use. The fishes were anesthetized by immersion in 0.1% ethyl m-aminobenzoate methanesulfonate (MS222; Sigma, St. Louis, MO, USA), which was neutralized to pH 7.4 with sodium bicarbonate prior to use, and then decapitated. The tissues for RNA preparation were removed with ophthalmic scissors and frozen in liquid nitrogen. Tissues other than ovary and testis were once pooled without distinguishing between males and females. Ovary and testis were obtained from females and males, respectively, and pooled. Total RNA was isolated from the three-spined stickleback and humphead wrasse tissues by acid guanidinium thiocyanate-phenol-chloroform extraction using Isogen reagent (Nippon Gene, Tokyo, Japan) according to the manufacturer’s manual. Owing to the small size of the three-spined sticklebacks, organs from three or more individuals were pooled for RNA extraction. Because only one 230-gram individual of humphead wrasse was available, RNA was extracted from organs derived from one individual. The RNA was dissolved in diethyl pyrocarbonate (DEPC)-treated water and its concentration was estimated by measuring the absorbance at 260 nm. mRNA preparations were reverse-transcribed into cDNA using the oligo(dT) primer and the SuperScript III First-Strand Synthesis System (Invitrogen). The cDNA (0.25 μL of the Super Script III reaction) was used as the template for PCRs, along with the specific primers shown in Supplementary Table 17. The PCR reactions were performed as follows^92^. Each reaction mixture (final volume, 12.5 μL) consisted of 0.25 μL cDNA (template), primers (individual final concentration, 0.25 μM), and 6.25 μL GoTaq Green Master Mix (2×; Promega, Madison, WI, USA). The PCR conditions were as follows: initial denaturation at 94 °C for 2 min, 28 or 33 cycles of 94 °C for 15 s (denaturation), 55 °C for 30 s (annealing), 72 °C for 1 min (extension), and a final extension at 72 °C for 7 min. PCR products from the three-spined sticklebacks were separated on agarose gels and visualized with ethidium bromide. The fluorescence images were analyzed with a Kodak Image Station 2000R system (Eastman Kodak, Rochester, NY, USA). The PCR products from the humphead wrasse were diluted and loaded onto a Microchip Electrophoresis system for DNA/RNA analysis (MCE-202 MultiNA; Shimadzu, Kyoto, Japan) using a DNA-12000 reagent kit (Shimadzu) following to the manufacturer’s instructions. Electrophoresis results were analyzed using the MultiNA Viewer software (Shimadzu). Images of the gels are shown in Supplementary Fig. 11.

### In situ hybridization histochemistry

In situ hybridization was performed as previously described in ref. ^93^. For tissue fixation, three-spined sticklebacks were anesthetized by immersion in 0.1% MS222, neutralized to pH 7.4, treated with sodium bicarbonate before use, and then decapitated. The stomach of three-spined sticklebacks was fixed in 4% paraformaldehyde in 0.1 M phosphate buffer at pH 7.4 for 1 d at 4 °C. Tissues were dehydrated, embedded in paraplast (Leica Microsystems, Wetzlar, Germany), and cut in 5 μm slices. For in situ hybridization, sections were deparaffinized in xylene, rehydrated by serial alcohol solutions, treated with proteinase K (5 μg/mL) for 10 min, and postfixed in 4% paraformaldehyde in 0.1 M phosphate buffer at pH 7.4. The sections were equilibrated in hybridization buffer (5× SSC and 50% formamide) at 58 °C for 2 h. A partial sequence of each target gene was cloned into the pGEM-T Easy vector (Promega) using the primers listed in Supplementary Table 17. Sense and antisense probes were prepared using a digoxigenin (DIG) RNA labeling kit (Roche Applied Science, Indianapolis, IN, USA), diluted in hybridization buffer containing calf thymus DNA (40 μg/mL), and denatured at 85 °C for 10 min. Denatured RNA probes were spread on the sections and incubated at 58 °C for >40 h depending on the expression level in a moist chamber saturated with hybridization buffer. Specific signals were developed using a DIG nucleic acid detection kit (Roche Applied Science), according to the manufacturer’s protocol. Some sections were stained with hematoxylin and eosin (H&E) or periodic acid–Schiff to determine the basic structure of epithelial cells. Images were obtained using a TOCO automatic virtual slide system (Path Imaging, Tokyo, Japan) and a microscope equipped with a digital CCD camera (AxioCam HRc; Carl Zeiss, Oberkochen, Germany), and processed using AxioVision 4.1 software (Carl Zeiss).

### Calculation of nucleotide substitution rates

Nucleotide sequences for claudin 18 were obtained from GenBank or Ensembl. We used three nucleotide sequences for tetrapod/coelacanth *cldn18* from human, tropical clawed frog, and coelacanth, three for each of gastric fish *cldn18a* and *cldn18b* from Atlantic cod, Nile tilapia, and three-spined stickleback, and two for agastric fish *cldn18b* from zebrafish and Japanese pufferfish. We used transcriptional sequences predicted from genome data when mRNA data was not available from the databases. The coding regions were aligned using ClustalW software^94^ and sites containing gaps were deleted manually without shifting the reading frame (Supplementary Fig. 12). Distance values for the non-synonymous substitutions per site (dn) and synonymous substitutions per site (ds) were calculated based on the Nei-Gojobori (NG) method^95^ using the alignment composed of 11 sequences and 522 positions and the MEGA6 software^96^. Standard errors were computed using the bootstrap method with 500 replicates. The number of non-synonymous differences (n), synonymous differences (s), non-synonymous sites (N), and synonymous sites (S) was calculated based on the Nei-Gojobori (NG) method using the MEGA6 software. Fisher’s exact test was used for the statistical analyses^97^.

### Synteny analysis of monotremes and related species

The presence or absence of *atp4a*, *atp4b*, *pga*, *pgc*, and *vsig1* was confirmed by BLAST search (TBLASTN) and synteny analysis using the genome databases of coelacanth^37^, *Xenopus*^38^, anole lizard^39^, platypus^40^, echidna^25^, and human^41^. Synteny analysis was performed manually using the Ensembl genome browser (https://www.ensembl.org)^98^ or the NCBI genome data viewer (https://www.ncbi.nlm.nih.gov/genome/gdv/)^99^.

### Statistics and reproducibility

All experiments using the three-spined stickleback and humphead wrasse were repeated at least twice, and reproducibility was confirmed using the same sample. For the statistical analyses of the of nucleotide substitution rates, we used three nucleotide sequences for tetrapod/coelacanth *cldn18*, three for each of gastric fish *cldn18a* and *cldn18b*, and two for agastric fish *cldn18b*. The numbers of sites for the statistical analyses are shown in Fig. 6d. Average numbers of non-synonymous differences (n) and unchanged non-synonymous sites (N-n) of gastric fish *cldn18b* were compared with those of agastric fishes, zebrafish, and Japanese pufferfish using by two-tailed Fisher’s exact test using GraphPad Prism (GraphPad, San Diego, CA, USA) (https://www.graphpad.com/quickcalcs/contingency1/). Average numbers of synonymous differences (s) and unchanged synonymous sites (S-s) were also analyzed similarly by two-tailed Fisher’s exact test.

### Reporting summary

Further information on research design is available in the Nature Portfolio Reporting Summary linked to this article.

### Supplementary information

Supplementary table and figures

Reporting summary

## Data Availability

All resources are available from the authors upon reasonable request.
