# Supplementary material for: Convergent gene losses and pseudogenizations in multiple lineages of stomachless fishes
Source: Commun Biol. 2024 Apr 3;7:408. doi: 10.1038/s42003-024-06103-x (PMC10991444; doi:10.1038/s42003-024-06103-x)
Supplement: Supplementary file 1 — Supplementary table and figures [file 42003_2024_6103_MOESM1_ESM.pdf]

Supplementary Table 1. Analysis of the synteny of *slc26a9* in the genomes of ray-finned fishes

Species	Chromosome, scaffold, etc.	direction	Annotated genes in each region													
Spotted gar<br>(*Lepisosteus oculatus*)	Linkage group 3	plus										slc26a9><br>XM_015342147.1				
Asian arowana<br>(*Scleropages formosus*)	Primary assembly 19	plus					tfcp2><br>XM_018760629.2	csrnp2><br>XM_018760311.2	itga5 ><br>XM_029246214.1	rapgef3<<br>XM_018760696.2	slc48a1><br>XM_018760202.2	slc26a9<<br>XM_018760766.2				
Golden-line barbell<br>(*Sinocyclocheilus grahami*)	LCYQ01S000013.1	plus					tfcp2><br>ENSSGRG00000019902	csrnp2><br>ENSSGRG00000019901	itga5><br>ENSSGRG00000019900	rapgef3<<br>ENSSGRG00000018515	slc48a1><br>ENSSGRG00000018509			mir205<<br>ENSSGRG00000018507	plxna2><br>ENSSGRG00000018505	
Zebrafish<br>(*Danio rerio*)	Chromosome 23	minus					tfcp2><br>XM_005162355.4	csrnp2><br>XM_001343376.8	itga5 ><br>NM_001004288.2	rapgef3<<br>XM_009297025.3	slc48a1><br>NM_001002424.2		hrct1><br>NM_001204373.1	tmdd1<<br>ENSDARG00000026864	mir205<<br>NR_029935.1	plxna2><br>XM_684688.8
Fathead minnow<br>(*Pimephales promelas*)	NW_024121654.1	plus				tfcp2><br>XM_039652438.1	csrnp2><br>XM_039652625.1	itga5 ><br>XM_039651644.1	~~~~~	rapgef3<<br>XM_039653116.1	slc48a1><br>XM_039652623.1		hrct1><br>XM_039652907.1	tmdd1<<br>XM_039653047.1	plxna2><br>XM_039652640.1	
Channel catfish<br>(*Ictalurus punctatus*)	Chromosome 15	plus					tfcp2><br>XM_017486584.3	csrnp2><br>XM_017486581.3	itga5 ><br>XM_017486560.3	rapgef3<<br>XM_017486561.3	slc48a1><br>XM_017486587.3	slc26a9><br>XM_017488141.3	hrct1><br>XM_017488137.3	tmdd1<<br>XM_053686288.1	plxna2><br>XM_047160545.2	
Mexican tetra<br>(*Astyanax mexicanus*)	Chromosome 13	minus					tfcp2><br>XM_007232570.4	csrnp2><br>XM_007232572.4	itga5 ><br>XM_022677267.2	rapgef3<<br>XM_022677261.2	slc48a1><br>XM_007258796.4	slc26a9><br>XM_049486477.1	hrct1><br>XM_022677256.2	tmdd1<<br>XM_049486480.1	plxna2><br>XM_049486476.1	
Rainbow trout<br>(*Oncorhynchus mykiss*)	Chromosome 16	minus					tfcp2><br>XM_021566116.2	csrnp2><br>XM_021566115.2	itga5 ><br>XM_036947020.1	rapgef3<<br>XM_021566108.2	slc48a1><br>XM_021566106.2	slc26a9><br>XM_021566104.2			plxna2><br>XM_036947019.1	
Northern pike<br>(*Esox lucius*)	Chromosome 12	plus					tfcp2><br>XM_010890547.4	csrnp2><br>XM_010890546.4	itga5 ><br>XM_010890544.3	rapgef3<<br>XM_010890540.4	slc48a1><br>XM_010890535.4	slc26a9><br>XM_010890537.5	tmdd1<<br>XM_020051527.2	mir205<<br>ENSELUG00000027729	plxna2><br>XM_034295893.1	
Atlantic cod<br>(*Gadus morhua*)	Chromosome 1	plus					tfcp2><br>XM_030353995.1	csrnp2><br>XM_030353984.1	itga5 ><br>XM_030353870.1	rapgef3<<br>XM_030353882.1	slc48a1><br>XM_030360250.1	slc26a9><br>XM_030360227.1	tmdd1<<br>XM_030364336.1	mir205<<br>ENSGMOG00000021542	plxna2><br>XM_030369078.1	
Greater amberjack<br>(*Seriola dumerili*)	NW_019174723.1	plus					tfcp2><br>XM_022763128.1	csrnp2><br>XM_022763063.1	itga5 ><br>XM_022763422.1	rapgef3<<br>XM_022763426.1	slc48a1><br>XM_022763305.1	slc26a9><br>XM_022763064.1	tmdd1<<br>ENSSDUG00000003143		plxna2><br>XM_022763109.1	
Japanese medaka<br>(*Oryzias latipes*)	Chromosome 7	plus					tfcp2><br>XM_004070893.4	csrnp2><br>XM_023956825.1	itga5 ><br>XM_023956830.1	rapgef3<<br>XM_023956833.1	slc48a1><br>XM_023956836.1		tmdd1<<br>XM_023956838.1	mir205<<br>NR_107117.2	plxna2><br>XM_004070981.4	
Turquoise killifish<br>(*Nothobranchius furzeri*)	NW_026539923.18	minus					tfcp2><br>XM_015967078.2	csrnp2><br>XM_054745719.1	itga5 ><br>XM_015967079.2	rapgef3<<br>XM_015967082.2	slc48a1><br>XM_015967085.2		tmdd1<<br>XM_015966952.2	~~~~~~~~~~~~~~	plxna2><br>XM_015967086.2	
Platyfish<br>(*Xiphophorus maculatus*)	Chromosome 1	plus					tfcp2><br>XM_023340295.1	csrnp2><br>XM_005798959.3	itga5 ><br>XM_023331342.1	rapgef3<<br>XM_023331356.1	slc48a1><br>XM_005798916.2		tmdd1<<br>XP_023186382.1		plxna2><br>XM_005798917.2	
Nile tilapia<br>(*Oreochromis niloticus*)	Linkage group 20	minus					tfcp2><br>XM_025901735.1	csrnp2><br>XM_005448442.3	itga5 ><br>XM_005448439.4	rapgef3<<br>XM_025901642.1	slc48a1><br>XM_003438892.4	slc26a9><br>XM_025901283.1	tmdd1<<br>XM_003439078.4	mir205<<br>ENSONIG00000021932	plxna2><br>XM_005448437.4	
Clown anemonefish<br>(*Amphiprion ocellaris*)	Chromosome 8	plus					tfcp2><br>XM_023289881.3	csrnp2><br>XM_023291104.3	itga5 ><br>XM_023289758.3	rapgef3<<br>XM_023289259.3	slc48a1><br>XM_023289456.3	slc26a9><br>XM_055012914.1			plxna2><br>XM_035958021.2	
Humphead wrasse<br>(*Cheilinus undulatus*)	Linkage Group 11	plus					tfcp2><br>XM_041799195.1	csrnp2><br>XM_041799638.1	itga5 ><br>XM_041798942.1	rapgef3<<br>XM_041798944.1	slc48a1><br>XM_041800394.1	slc26a9><br>XM_041800393.1	tmdd1<<br>XM_041799047.1		plxna2><br>XM_041799400.1	
Ballan wrasse<br>(*Labrus bergylta*)	NW_018115349.1	plus									slc48a1><br>XM_020657924.2	slc26a9><br>XM_029282163.1	tmdd1<<br>XM_029282164.1			
Gilthead seabream<br>(*Sparus aurata*)	Chromosome 7	plus					tfcp2><br>XM_030422482.1	csrnp2><br>XM_030423941.1	itga5 ><br>XM_030423142.1	rapgef3<<br>XM_030423144.1	slc48a1><br>XM_030424328.1	slc26a9><br>XM_030423641.1	tmdd1<<br>XM_030423677.1		plxna2><br>XM_030421747.1	
Three-spined stickleback<br>(*Gasterosteus aculeatus*)	Chromosome 12	minus					tfcp2><br>XM_040193529.1	csrnp2><br>XM_040193527.1	itga5 ><br>XM_040192481.1	rapgef3<<br>XM_040192483.1	slc48a1><br>XM_040192491.1	slc26a9><br>XM_041800393.1	tmdd1<<br>XM_041799047.1	mir205<<br>ENSGACG00000021334	plxna2><br>XM_040193258.1	
Ocean sunfish<br>(*Mola mola*)	KV751321.	minus					tfcp2><br>ENSMMOG00000017275	csrnp2><br>ENSMMOG00000017272	itga5 ><br>ENSMMOG00000017241	rapgef3<<br>ENSMMOG00000017224	slc48a1><br>ENSMMOG00000017219	slc26a9><br>ENSMMOG00000017215			plxna2><br>ENSMMOG00000017213	
Japanese pufferfish<br>(*Takifugu rubripes*)	Chromosome 3	minus	plxna2<<br>XM_029833484.1	mir205><br>NR_105312.1	tmdd1><br>XM_011619283.2	~~~~~~~~~~~~~~	tfcp2><br>XM_011602704.2	csrnp2><br>XM_003963516.3	itga5 ><br>XM_003963515.3	rapgef3<<br>XM_011602701.2	slc48a1><br>XM_003963459.3					
Spotted green pufferfish<br>(*Tetraodon nigroviridis*)	Chromosome 9	minus	plxna2<<br>ENSTNIG00000012467	mir205><br>ENSTNIG00000019948	tmdd1><br>ENSTNIG0000000108	~~~~~~~~~~~~~~	tfcp2><br>ENSTNIG00000012528	csrnp2><br>ENSTNIG00000012529	itga5 ><br>ENSTNIG00000012530	rapgef3<<br>ENSTNIG00000012531	slc48a1><br>ENSTNIG00000012532					

Supplementary Table 2. Analysis of the synteny of *kcne2* in the genomes of ray-finned fishes

Species	Chromosome, scaffold, etc.	direction	Annotated genes in each region										
Spotted gar<br>(*Lepisosteus oculatus*)	Linkage group 17	minus		<morc3<br>XM_015363815.1	<dopey2<br>XM_015363815.1	~~~~~~~~~~~	< atp5o<br>XM_006639040.2	kcne2><br>XM_015363720.1	~~~~~~~~~~~~~~~~~	>casq2<br>XM_015363888.1	<vangl1<br>XM_006639037.2	<nhlh2<br>XM_015363867.1	
Asian arowana<br>(*Scleropages formosus*)	Chromosome 12	plus		<morc3<br>XM_018757538.2<br>XM_018759204.2	<dop1b<br>XM_018759028.2	~~~~~~~~~~~	>slc5a3<br>XM_018757487.2	kcne2><br>XM_018757706.1	~~~~~~~~~~~~~~~~~		<vangl1<br>XM_018757500.2	<nhlh2<br>XM_018758210.2	
Golden-line barbell<br>(*Sinocyclocheilus grahami*)	LCYQ01S000209.1	plus			<dopey2<br>ENSSGRG00000052099	< atp5o<br>ENSSGRG00000052103	>slc5a3<br>ENSSGRG00000052104		>casq2<br>ENSSGRG00000052111	~~~~~~~~~~~~~~~~~	<vangl1<br>ENSSGRG00000052175		
Zebrafish<br>(*Danio rerio*)	Chromosome 9	minus			<dopey2<br>XM_017357831.2	< atp5o<br>NM_001003843.1	>slc5a3b<br>XM_005167791.4		>si:ch211-125e6.8<br>XM_021478580.1	>casq2<br>NM_001002682.1	~~~~~~~~~~~~~~~~~	<vangl1<br>XM_005167685.4	<nhlh2<br>NM_205669.1
Fathead minnow<br>(*Pimephales promelas*)	NW_024121654.1	minus	<nhlh2<br>XM_039653104.1<br>XM_017469583.2	~~~~~~~~~~~	<dop1b<br>XM_039653089.1	< atp5o<br>XM_039653101.1	>slc5a3b<br>XM_039653093.1		>casq2<br>XM_039653098.1	< ladderlectin-like<br>XM_039653102.1	< vangl1<br>XM_039653097.1		
Channel catfish<br>(*Ictalurus punctatus*)	Chromosome 6	minus	<morc3<br>XM_053680702.1	<dop1b<br>XM_017469588.3	>drc1<br>XM_017469196.3	< atp5o<br>XM_017470898.2	>slc5a3b<br>XM_017470590.3	>kcne2<br>XM_017470892.2	>casq2<br>XM_017470889.3	<vangl1<br>XM_017470876.3	<nhlh2<br>XM_017469350.3		
Mexican tetra<br>(*Astyanax mexicanus*)	Chromosome 11	plus		<morc3a<br>XM_022665631.2	<dop1b<br>XM_007227734.4	< atp5o<br>XM_007227738.3	>slc5a3b<br>XM_007227737.4	>kcne2<br>XM_049485171.1	>casq2<br>XM_007227741.4	<vangl1<br>XM_022665627.2	<nhlh2<br>XM_007227744.4		
Rainbow trout<br>(*Oncorhynchus mykiss*)	Chromosome 22	plus		<morc3<br>XM_036959588.1	<dopey2<br>XM_021579331.2	< atp5o<br>XM_021579335.2	>slc5a3<br>XM_021579332.2	>kcne2<br>XM_021579336.2	>casq2<br>NM_001160499.1	<vangl1<br>XM_021579339.2	<nhlh2<br>XM_021579340.2		
Northern pike<br>(*Esox lucius*)	Chromosome 16	minus		<morc3a<br>XM_029113516.2	<dop1b<br>XM_029113436.2	< atp5o<br>XM_010905505.2	>slc5a3b<br>XM_010905501.4	>kcne2<br>XM_010905502.5	>casq2<br>XM_010905500.4	<vangl1<br>XM_020054487.2	<nhlh2<br>XM_010869165.2	<nqo1<br>XM_029113598.2	
Atlantic cod<br>(*Gadus morhua*)	Chromosome 20	minus				<dop1b<br>XM_030343574.1	>slc5a3<br>XM_030344192.1	>kcne2<br>XM_030343996.1	>casq2<br>XM_030343995.1	< vangl1<br>XM_030344037.1	<nhlh1<br>XM_030344019.1	<nqo2<br>XM_030344326.1	
Greater amberjack<br>(*Seriola dumerili*)	NW_019174307.1	minus			<morc3<br>XM_022740659.1	<dopey2<br>XM_022740425.1	>slc5a3<br>XM_022740817.1	>kcne2<br>XM_022741389.1	>casq2<br>XM_022740470.1	<vangl1<br>XM_022741388.1	<nhlh2<br>XM_022741288.1	<nqo1<br>XM_022741100.1	
Japanese medaka<br>(*Oryzias latipes*)	Chromosome 21	minus			<morc3<br>XM_023950535.1	<dopey2<br>XM_023950572.1	>slc5a3<br>XM_004081962.4		>casq2<br>XM_004081796.4	<vangl1<br>XM_004081793.4		>nqo2<br>XM_020713178.2	
Turquoise killifish<br>(*Nothobranchius furzeri*)	Chromosome sgr14	plus			<morc3a<br>XM_015966324.2	<dop1b<br>XM_054746853.1	>slc5a3b<br>XM_015966328.2		>casq2<br>XM_054746857.1	<vangl1<br>XM_054746856.1	<nhlh1<br>XM_054746436.1	>nqo2<br>XM_015966331.2	
Platyfish<br>(*Xiphophorus maculatus*)	Chromosome 7	plus			<morc3a<br>XM_023337257.1	<dopey2<br>XM_023336859.1	>slc5a3b<br>XM_005799366.2		>casq2<br>XM_005799314.2	<vangl1<br>XM_023336249.1			
Nile tilapia<br>(*Oreochromis niloticus*)	Linkage group 16	minus			<morc3<br>XM_005460014.4	<dop1b<br>XM_013264244.3	>slc5a3b<br>XM_005460016.4	>kcne2<br>XM_003455440.5	>casq2<br>XM_003455449.5	<vangl1<br>XM_005460017.4	<nhlh1<br>XM_019352666.2		
Clown anemonefish<br>(*Amphiprion ocellaris*)	Chromosome 11	plus			<morc3a<br>XM_055015402.1	<dop1b<br>XM_023285909.3	>slc5a3b<br>XM_023285917.3	>kcne2<br>XM_023285899.3	>casq2<br>XM_023285928.3	<vangl1<br>XM_023285962.3	<nhlh2<br>XM_023285878.3	>nqo2<br>XM_023285968.2	
Humphead wrasse<br>(*Cheilinus undulatus*)	Linkage group 15	plus			<morc3a<br>XM_041807808.1	<dop1b<br>XM_041807739.1	>slc5a3b<br>XM_041807742.1		>casq2<br>XM_041807800.1	<vangl1<br>XM_041807887.1		>nqo1<br>XM_041807377.1	
Ballan wrasse<br>(*Labrus bergylta*)	NW_018115239.1	plus			<morc3<br>XM_020657119.2	<dopey2<br>XM_029281964.1	>slc5a3<br>XM_020657120.2		>casq2<br>XM_020657117.2	<vangl1<br>XM_020657117.2			
Gilthead seabream<br>(*Sparus aurata*)	Chromosome 9	plus			<morc3<br>XM_030429339.1	<dopey2<br>XM_030428296.1	>slc5a3<br>XM_030427748.1	>kcne1<br>XM_030427756.1	>casq2<br>XM_030427288.1	<vangl1<br>XM_030429313.1	<nhlh2<br>XM_030428978.1	>nqo2<br>XM_030427506.1	
Three-spined stickleback<br>(*Gasterosteus aculeatus*)	Chromosome 16	plus			<morc3a<br>XM_040201698.1	<dop1b<br>XM_040200801.1	>slc5a3b<br>XM_040200806.1	>kcne2<br>XM_040202413.1	>casq2<br>XM_040202412.1	<vangl1<br>XM_040201974.1	<nhlh2<br>XM_040201971.1	>nqo2<br>XM_040200610.1	
Ocean sunfish<br>(*Mola mola*)	KV751321.1	plus				<dopey2<br>ENSMMOG00000020033<br>ENSMMOG00000020029	>slc5a3<br>ENSMMOG00000020035		>casq2<br>ENSMMOG00000020036	<vangl1<br>ENSMMOG00000020037		>nqo<br>ENSMMOG00000020038	
Japanese pufferfish<br>(*Takifugu rubripes*)	Chromosome 1	plus			<morc3<br>XM_029834956.1	<dop1b<br>XM_011608937.2	>slc5a3b<br>XM_003961854.3		>casq2<br>XM_029834556.1	<vangl1<br>XM_029834535.1		>nqo1<br>XM_011608975.2	
Spotted green pufferfish<br>(*Tetraodon nigroviridis*)	Chromosome 2	plus			<morc3a<br>ENSTNIG00000013941	<dop1b<br>ENSTNIG00000013942	>slc5a3b<br>ENSTNIG00000013943		>casq2<br>ENSTNIG00000013944	<vangl1<br>ENSTNIG00000013945			

Supplementary Table 3. Analysis of the synteny of *cldn18a* in the genomes of ray-finned fishes

Species	Chromosome, scaffold, etc.	direction	Annotated genes in each region									
Spotted gar (*Lepisosteus oculatus*)	Linkage group 14	plus				dzip1l><br>XM_015361098.1	cldn18<<br>XM_015361319.1	sox14><br>XM_006637666.2	~~~~~~~~~~~	hdlbpa<<br>XM_015361245.1		
Asian arowana (*Scleropages formosus*)	Chromosome 3	plus				med8><br>XM_018738571.1	cldn18<<br>XM_018738412.1	sox14><br>XM_029250646.1			ephexin-1><br>XM_029250396.1	farp2><br>XM_018738699.1
Golden-line barbell (*Sinocyclocheilus grahami*)	LCYQ01S000166.1	plus		lmo4b<<br>ENSSGRG00000048438	hs2st1b<<br>ENSSGRG00000048440	dzip1l><br>ENSSGRG00000048441		sox14<<br>ENSSGRG00000048443		hdlbpa<<br>ENSSGRG00000048464		farp2><br>ENSSGRG00000049963
Zebrafish (*Danio rerio*)	Chromosome 6	plus		lmo4b<<br>NM_212689.2	hs2st1b<<br>XM_009302420.3	dzip1l><br>NM_001037227.1		sox14<<br>NM_001037680.1		hdlbpa<<br>XM_005165938.4	ephexin-1><br>XM_021477114.1	farp2><br>XM_009302421.2
Fathead minnow (*Pimephales promelas*)	Chromosome Un	plus	lmo4b<<br>XM_039691635.1	~~~~~~~~~~~	hs2st1b<<br>XM_039691659.1	dzip1l><br>XM_039691413.1		sox14<<br>XM_039691403.1		hdlbpa<<br>XM_039691408.1		farp2><br>XM_039691516.1
Channel catfish (*Ictalurus punctatus*)	Chromosome 11	plus		lmo4b<<br>XM_047158505.1	hs2st1b<<br>XM_017479168.2	dzip1l><br>XM_017479170.2	cldn18a<<br>XM_017479160.2	sox14<<br>XM_017479107.2		hdlbpa<<br>XM_017479848.2	ephexin-1><br>XM_017479114.2	farp2><br>XM_017479653.2
Mexican tetra (*Astyanax mexicanus*)	Chromosome 5	minus		lmo4b<<br>XM_007235863.4	hs2st1b<<br>XM_049479321.1	dzip1l><br>XM_049479320.1	cldn18<<br>XM_007253651.3	sox14<<br>XM_007253652.4		hdlbpa<<br>XM_049479081.1	ephexin-1><br>XM_022675440.2	farp2><br>XM_022675446.2
Rainbow trout (*Oncorhynchus mykiss*)	Chromosome 5	plus		lmo4b<<br>XM_036978903.1	hs2st1b<<br>XM_036978904.1	dzip1l><br>XM_036978906.1	cldn18a<<br>XM_021604809.2	sox14<<br>XM_021604810.2	~~~~~~~~~~~	hdlbpa<<br>XM_036978909.1	ephexin-1><br>XM_036978915.1	farp2><br>XM_036978910.1
Northern pike (*Esox lucius*)	Chromosome 8	plus			lmo4b<<br>XM_029121433.2	~~~~~~~~~~~	cldn18a<<br>XM_020044587.3	sox14<<br>XM_010863674.3	~~~~~~~~~~~	hdlbpa<<br>XM_010865313.5		farp2><br>XM_020044319.2
Atlantic cod (*Gadus morhua*)	Chromosome 12	minus			lmo4b<<br>XM_030372422.1	hs2st1b<<br>XM_030372420.1	cldn18a<<br>XM_030372424.1	sox14<<br>XM_030372421.1		hdlbpa<<br>XM_030372276.1		farp2><br>XM_030372531.1
Greater amberjack (*Seriola dumerili*)	Chromosome Un	minus			lmo4b<<br>XM_022759409.1	hs2st1b<<br>XM_022759388.1	cldn18a<<br>XM_022759175.1	sox14<<br>XM_020630941.2		hdlbpa<<br>XM_020631166.2		farp2><br>XM_020631207.2
Japanese medaka (*Oryzias latipes*)	Chromosome 4	minus			lmo4b<<br>XM_023954029.1	hs2st1b<<br>XM_023954027.1		sox14<<br>NM_001164872.1		hdlbpa<<br>XM_011473777.3		farp2><br>XM_011473774.3
Turquoise killifish (*Nothobranchius furzeri*)	Chromosome sgr12	minus			lmo4b<<br>XM_015962340.1	hs2st1b<<br>XM_015962342.1		sox14<<br>XM_015962343.1		hdlbpa<<br>XM_015962344.1		farp2><br>XM_015962349.1
Platyfish (*Xiphophorus maculatus*)	Chromosome 9	minus			lmo4b<<br>XM_005813177.3	hs2st1b<<br>XM_005813178.3		sox14<<br>XM_023340038.1		hdlbpa<<br>XM_014473429.2		farp2><br>XM_023339459.1
Nile tilapia (*Oreochromis niloticus*)	Linkage group 23	plus			lmo4b<<br>XM_005451175.4	hs2st1b<<br>XM_003444265.5	cldn18a<<br>XM_003444205.5	sox14<<br>XM_005451176.4		hdlbpa<<br>XM_003444207.5		farp2><br>XM_005451179.4
Clown anemonefish (*Amphiprion ocellaris*)	Chromosome Un	plus					cldn18a<<br>XM_023281455.2	sox14<<br>XM_023281451.2		hdlbpa<<br>XM_023281444.2		farp2><br>XM_035953439.1
Humphead wrasse (*Cheilinus undulatus*)	Linkage group 7	plus			lmo4b<<br>XM_041790587.1	hs2st1b<<br>XM_041791882.1		sox14<<br>XM_041791513.1		hdlbpa<<br>XM_041791224.1		farp2><br>XM_041790846.1
Ballan wrasse (*Labrus bergylta*)	Chromosome Un	minus			lmo4b<<br>XM_020630767.2	hs2st1b<<br>XM_020633174.1		sox14<<br>XM_020630941.2		hdlbpa<<br>XM_020631166.2		farp2><br>XM_020631207.2
Gilthead seabream (*Sparus aurata*)	Chromosome 11	plus			lmo4b<<br>XM_030433408.1	hs2st1b<<br>XM_030433407.1	cldn18a<<br>XM_030432476.1	sox14<<br>XM_030434600.1		hdlbpa<<br>XM_030433015.1		farp2><br>XM_030433125.1
Three-spined stickleback (*Gasterosteus aculeatus*)	Chromosome 8	plus			lmo4b<<br>XM_040184881.1	hs2st1b<<br>XM_040184702.1	cldn18a<<br>XM_040183297.1	sox14<<br>XM_040185113.1				
Ocean sunfish (*Mola mola*)	KV751319.1	minus			lmo4b<<br>ENSMMOG00000006889	hs2st1b<<br>ENSMMOG00000006886		sox14<<br>ENSMMOG00000006884		hdlbpa<<br>ENSMMOG00000006880		farp2><br>ENSMMOG00000006872
Japanese pufferfish (*Takifugu rubripes*)	Chromosome 20	minus			lmo4b<<br>XM_011614452.2	hs2st1b<<br>XM_003973818.3		sox14<<br>XM_011614450.2		hdlbpa<<br>XM_003973786.3		farp2><br>XM_011614448.2
Spotted green pufferfish (*Tetraodon nigroviridis*)	Chromosome Un	minus		hs2st1b><br>ENSTNIG00000005881	hs2st1b<<br>ENSTNIG00000005880							

Supplementary Table 4. Analysis of the synteny of *cldn18b* in the genomes of ray-finned fishes

Species	Chromosome, scaffold, etc.	direction	Annotated genes in each region													
Spotted gar<br>(*Lepisosteus oculatus*)	Linkage group 10	plus					gft2b><br>XM_006634711.2	pkn2a <<br>XM_015355239.1								
Asian arowana<br>(*Scleropages formosus*)	Chromosome 25	minus	cldn18b<<br>XM_018731102.2	sox14<<br>XM_018731062.2	~~~~~~~~~~~~~	selenof<<br>XM_018730784.2	hs2st1a><br>XM_018731412.2	~~~~~~~~~~~~~	pkn2 ><br>XM_018731340.2	gft2b<<br>XM_018730825.1	lrrc8c><br>XM_018730747.2	lcc8d><br>XM_018731328.2	znf326><br>XM_018730813.2	barhl2<<br>XM_029249416.1	znf644a<<br>XM_018730832.2	hfm1><br>XM_018730780.2
Golden-line barbell<br>(*Sinocyclocheilus grahami*)	LCYQ01S000121.1	plus		selenof<<br>ENSSGRG00000051310	hs2st1a><br>ENSSGRG00000051311	~~~~~~~~~~~~~	pkn2a ><br>ENSSGRG00000051335	gft2b<<br>ENSSGRG00000051338	lrrc8da><br>ENSSGRG00000051404	znf644a<<br>ENSSGRG00000051405	cldn18b<<br>ENSSGRG00000051407	sox14<<br>ENSSGRG00000051408	septin2><br>ENSSGRG00000051409	stk25b<<br>ENSSGRG00000051411		
Zebrafish<br>(*Danio rerio*)	Chromosome 2	plus	selenof<<br>NM_178294.5	hs2st1a<<br>NM_001080670.1	~~~~~~~~~~~~~	pkn2a ><br>XM_695612.9	gft2b<<br>NM_199697.1	lrrc8da><br>XM_002660814.6	znf644a<<br>NM_001278254.1	hfm1><br>XM_021467172.1	cldn18b<<br>XM_002660816.5	sox14<<br>XM_009298505.2	septin2><br>XM_009298508.3	stk25b<<br>XM_005171203.4		
Fathead minnow<br>(*Pimephales promelas*)	Chromosome Un	minus	selenof<<br>XM_039678066.1	hs2st1a><br>XM_039677677.1	~~~~~~~~~~~~~	pkn2a ><br>XM_039676620.1	gft2b<<br>XM_039676621.1	lrrc8da><br>XM_039675765.1	znf644a<<br>XM_039675765.1	hfm1><br>XM_039678213.1	cldn18b<<br>XM_039678153.1	sox14<<br>XM_039675783.1	septin2><br>XM_039675779.1	stk25b<<br>XM_039675777.1		
Channel catfish<br>(*Ictalurus punctatus*)	Chromosome 17	plus				pkn2a ><br>XM_017491286.2	gft2b<<br>XM_017491336.2	lrrc8da><br>XM_017491291.2	znf644a<<br>XM_017491283.2	hfm1><br>XM_017491276.2	cldn18b<<br>NM_001329288.1		septin2><br>NM_001329322.1	stk25b<<br>XM_017491324.2		
Mexican tetra<br>(*Astyanax mexicanus*)	Chromosome 18	minus	selenof<<br>XM_007245089.4	hs2st1a><br>XM_007245087.4	~~~~~~~~~~~~~	pkn2a ><br>XM_022670309.2	gft2b<<br>XM_049466958.1	lrrc8da><br>XM_007245085.4	znf644a<<br>XM_015604784.3	hfm1><br>XM_049467114.1	cldn18b<<br>XM_007245171.3	sox14<<br>XM_007245170.4	septin2><br>XM_039675779.1	stk25b<<br>XM_022671210.2		
Rainbow trout<br>(*Oncorhynchus mykiss*)	Primary assembly 8	minus	hfm1<<br>ENSOMYG00000010165	znf644><br>ENSOMYG00000064160<br>ENSOMYG00000010108			gft2b><br>ENSOMYG00000010047	pkn2 <<br>ENSOMYG00000009735	hs2st1a<<br>ENSOMYG00000009479		cldn18b<<br>ENSOMYG00000050404	~~~~~~~~~~~~~	septin2b><br>ENSOMYG00000009064			
Northern pike<br>(*Esox lucius*)	Chromosome 3	minus	hfm1<<br>XM_029118606.2	znf644a><br>XM_010893507.4	znf326<<br>XM_020044435.2	lrrc8da<<br>XM_010893475.5	gft2b><br>XM_010893462.4	pkn2a <<br>XM_010893449.5	hs2st1a<<br>XM_010893392.3	selenof><br>XM_010893420.3	cldn18b<<br>XM_010893371.4	sox14<<br>XM_010893359.3	septin2><br>XM_010893338.5	stk25b<<br>XM_010893318.5		
Atlantic cod<br>(*Gadus morhua*)	Chromosome 8	plus	hfm1<<br>XM_030363683.1	znf644a><br>XM_030363683.1	znf326<<br>XM_030364946.1	lrrc8da<<br>XM_030364918.1	gft2b><br>XM_030364945.1	pkn2a <<br>XM_030364916.1	hs2st1a<<br>XM_030364941.1	selenof><br>XM_030364961.1	cldn18b<<br>XM_030364954.1	sox14<<br>XM_030364953.1	septin2><br>XM_030364936.1	stk25b<<br>XM_030364934.1		
Greater amberjack<br>(*Seriola dumerili*)	Chromosome Un	minus	hfm1<<br>XM_022755024.1	znf644a><br>XM_022754893.1	znf326<<br>XM_022755023.1	lrrc8da<<br>XM_022754898.1	gft2b><br>XM_022754919.1	pkn2<<br>XM_022754895.1	hs2st1a<<br>XM_022754917.1	selenof><br>XM_022754927.1	cldn18b<<br>XM_022754926.1	sox14<<br>XM_022755021.1	septin2><br>XM_022754915.1	stk25<<br>XM_022754914.1		
Japanese medaka<br>(*Oryzias latipes*)	Chromosome 17	minus	hfm1<<br>XM_023964812.1	znf644a><br>XM_011486123.3	znf326<<br>XM_023964827.1	lrrc8da<<br>XM_004078644.3	gft2b><br>XM_004078643.4	pkn2 <<br>XM_020710772.2	hs2st1a<<br>XM_004078642.4	selenof><br>XM_030364961.1		sox14<<br>XM_023964836.1	septin2><br>XM_011486111.3	stk25<<br>XM_004078639.4		
Turquoise killifish<br>(*Nothobranchius furzeri*)	Chromosome Un	plus								selenof><br>XM_015941034.1		sox14<<br>XM_015941035.1				
Platyfish<br>(*Xiphophorus maculatus*)	Chromosome 6	plus	hfm1<<br>XM_023334962.1	znf644a><br>XM_023334799.1		lrrc8da<<br>XM_023335953.1	gft2b><br>XM_023335954.1	pkn2a <<br>XM_023336144.1	hs2st1a<<br>XM_005802359.2	selenof><br>XM_005802360.2		sox14<<br>XM_005817153.2	septin2><br>XM_014470013.2	stk25b<<br>XM_005802361.3		
Nile tilapia<br>(*Oreochromis niloticus*)	Chromosome LG18	minus	hfm1<<br>XM_019348099.2	znf644a><br>XM_003438025.4	znf326<<br>XM_005476031.4	lrrc8da<<br>XM_003437786.5	gft2b><br>XM_019348123.2	pkn2<<br>XM_019348122.2	hs2st1a<<br>XM_003437788.5	selenof><br>XM_005476019.4	cldn18b<<br>XM_025900128.1	sox14<<br>XM_003438028.5	septin2><br>XM_005476016.4	stk25<<br>XM_003437789.5		
Clown anemonefish<br>(*Amphiprion ocellaris*)	Chromosome Un	plus					gft2b><br>XM_023270576.2	pkn2<<br>XM_035948328.1	hs2st1a<<br>XM_023270578.2	selenof><br>XM_023270579.1	cldn18b<<br>XM_035948329.1	sox14<<br>XM_023270577.2				
Humphead wrasse<br>(*Cheilinus undulatus*)	Linkage group 13	plus				lrrc8da<<br>XM_041803528.1	gtf2b><br>XM_041802438.1	pkn2a<<br>XM_041802437.1	hs2st1a<<br>XM_041802347.1	selenof><br>XM_041803769.1		sox14<<br>XM_041804024.1	sept2><br>XM_041803431.1	stk25b<<br>XM_041804245.1		
Ballan wrasse<br>(*Labrus bergylta*)	Chromosome Un	plus	hfm1<<br>XM_029280380.1	znf644a><br>XM_029280718.1	znf326<<br>XM_020649787.2		gft2b><br>XM_020650071.2	pkn2a<<br>XM_020649281.2	hs2st1a<<br>XM_020651956.2	selenof><br>XM_020650332.2		sox14<<br>XM_020650243.2	septin2><br>XM_020649908.2	stk25b<<br>XM_020649749.2		
Gilthead seabream<br>(*Sparus aurata*)	Chromosome 21	minus	hfm1<<br>XM_030402790.1	znf644a><br>XM_030402797.1	znf326<<br>XM_030402799.1	lrrc8d<<br>XM_030403788.1	gft2b><br>XM_030403806.1	pkn2a<<br>XM_030403785.1	hs2st1a<<br>XM_030403803.1	selenof><br>XM_030403816.1	cldn18b<<br>XM_030403811.1	sox14<<br>XM_030403814.1	septin2><br>XM_030403799.1	stk25b<<br>XM_030403797.1		
Three-spined stickleback<br>(*Gasterosteus aculeatus*)	Chromosome 3	plus	hfm1<<br>XM_040171691.1	znf644a><br>XM_040171700.1	~~~~~~~~~~~~~	lrrc8da<<br>XM_040171707.1	gft2b><br>XM_040171741.1	pkn2a <<br>XM_040171706.1	hs2st1a<<br>XM_040171740.1	selenof><br>XM_040171755.1	cldn18b<<br>XM_040171752.1	sox14<<br>XM_040171753.1	septin2><br>XM_040171738.1	stk25b<<br>XM_040171734.1		
Ocean sunfish<br>(*Mola mola*)	KV751338.1	minus	hfm1<<br>ENSMMOG00000006148	znf644a><br>ENSMMOG00000006142<br>ENSMMOG00000006138		lrrc8da<<br>ENSMMOG00000006135	gft2b><br>ENSMMOG00000006132	pkn2a<<br>ENSMMOG00000006128	hs2st1a<<br>ENSMMOG00000006127	selenof><br>ENSMMOG00000006125		sox14<<br>ENSMMOG00000006124			tmem125b><br>ENSMMOG00000006123	
Japanese pufferfish<br>(*Takifugu rubripes*)	Chromosome 22	plus	hfm1<<br>XM_029830792.1	znf644a><br>XM_011616550.2	znf326<<br>XM_011616549.2	lrrc8da<<br>XM_029830798.1	gft2b><br>XM_003975720.3	pkn2<<br>XM_011616547.2	hs2st1a<<br>XM_003975719.3	selenof><br>XM_003975767.3	cldn18b<<br>XM_011616659.2	sox14<<br>XM_029830557.1			tmem125-like><br>XM_003975825.2	
Spotted green pufferfish<br>(*Tetraodon nigroviridis*)	Chromosome 15	minus	hfm1<<br>ENSTNIG00000006962	znf644a><br>ENSTNIG00000006961		lrrc8da<<br>ENSTNIG00000001498	gft2b><br>ENSTNIG00000006960	pkn2<<br>ENSTNIG00000006959	hs2st1a<<br>ENSTNIG00000006958						tmem125b><br>ENSTNIG00000006956	

Supplementary Table 5. Analysis of the synteny of *vsig1* in the genomes of ray-finned fishes

Species	Chromosome, scaffold, etc.	direction	Annotated genes in each region								
Spotted gar<br>(*Lepisosteus oculatus*)	Linkage group 7	minus		brcc3><br>XM_006632616.2	~~~~~~~~~~~~~	gabra3<<br>XM_015351059.1	gabrb4><br>XM_006632615.2	vsig1><br>XM_015351107.1	~~~~~~~~~~~~~	gpr34l<<br>XM_015351102.1	mpp1><br>XM_006632614.2
Asian arowana<br>(*Scleropages formosus*)	Chromosome 4	minus			brcc3><br>XM_018752297.2		gabrb4><br>XM_018751752.1	vsig1><br>XM_029251115.1		gpr34l<<br>XM_018751876.1	
Golden-line barbell<br>(*Sinocyclocheilus grahami*)	LCYQ01S000039.1	plus			brcc3><br>ENSSGRG00000041751	gabra3<<br>ENSSGRG00000041764	gabrb4><br>ENSSGRG00000041802			gpr34l<<br>ENSSGRG00000041855	mpp1><br>ENSSGRG00000041859
Zebrafish<br>(*Danio rerio*)	Chromosome 21	minus			prrg3><br>XM_686654.7	~~~~~~~~~~~~~	gabrb4><br>XM_017353011.2			gpr34l<<br>NM_001007217.1	mpp1><br>NM_214692.1
Fathead minnow<br>(*Pimephales promelas*)	Chromosome Un	plus				gabra3<<br>XM_039650228.1	gabrb4><br>XM_039650222.1			gpr34l<<br>XM_039650209.1	mpp1><br>XM_039650221.1
Channel catfish<br>(*Ictalurus punctatus*)	Chromosome 18	minus			brcc3><br>XM_017493127.2	gabra3<<br>XM_017491857.2	gabrb4><br>XM_017491942.2	vsig1><br>XM_017492329.2		gpr34l<<br>XM_017492328.2	mpp1><br>XM_017492321.2
Mexican tetra<br>(*Astyanax mexicanus*)	Chromosome 17	minus			brcc3><br>XM_049466437.1	gabra3<<br>XM_049466513.1	gabrb4><br>XM_022676236.2	vsig1><br>XM_049466763.1		gpr34l<<br>XM_007231694.4	mpp1><br>XM_007231695.4
Rainbow trout<br>(*Oncorhynchus mykiss*)	Chromosome Y	minus				gabra3<<br>XM_036967506.1	gabrb4><br>XM_036967859.1	vsig1><br>XM_021591161.2		gpr34<<br>XM_021590272.2	mpp1><br>XM_021591158.2
Northern pike<br>(*Esox lucius*)	Chromosome 7	plus			brcc3><br>XM_010900551.4	gabra3<<br>XM_020048670.2	gabrb4><br>XM_010894615.3	vsig1><br>XM_010894616.5		gpr34l<<br>XM_010894617.4	mpp1><br>XM_010894620.5
Atlantic cod<br>(*Gadus morhua*)	Chromosome 7	minus	ammecr1><br>XM_030361293.1	tmem164<<br>XM_030361294.1	~~~~~~~~~~~~~	gabra3<<br>XM_030361292.1	gabrb4><br>XM_030361266.1	vsig1><br>XM_030361295.1	tmlhe<<br>XM_030361278.1		mpp1><br>XM_030361276.1
Greater amberjack<br>(*Seriola dumerili*)	Chromosome Un	minus	ammecr1><br>XM_022745768.1	tmem164<<br>XM_022745869.1		gabra3<<br>XM_022746088.1	gabrb4><br>XM_022745861.1	vsig1><br>XM_022746087.1	tmlhe<<br>XM_022746086.1		mpp1><br>XM_022745808.1
Japanese medaka<br>(*Oryzias latipes*)	Chromosome 14	minus	ammecr1><br>XM_023962852.1	tmem164<<br>XM_004076579.4		gabra3<<br>XM_023962376.1	gabrb4><br>XM_023962349.1		tmlhe<<br>XM_023962789.1		mpp1><br>XM_004076576.3
Turquoise killifish<br>(*Nothobranchius furzeri*)	Chromosome sgr11	minus				gabra3<<br>XM_015961151.1	gabrb4><br>XM_015961149.1		tmlhe<<br>XM_015961147.1		mpp1><br>XM_015961145.1
Platyfish<br>(*Xiphophorus maculatus*)	Chromosome 11	minus	ammecr1><br>XM_023342220.1	tmem164<<br>XM_005800755.3		gabra3<<br>XM_023342212.1	gabrb4><br>XM_005800753.3		tmlhe<<br>XM_005800752.3		mpp1><br>XM_023342215.1
Nile tilapia<br>(*Oreochromis niloticus*)	Linkage group 10	minus	ammecr1><br>XM_003450479.5	tmem164<<br>XM_005471946.4		gabra3<<br>XM_019364101.2	gabrb4><br>XM_003450447.5	vsig1><br>XM_003450448.5	tmlhe<<br>XM_019364093.2	~~~~~~~~~~~~~	mpp1><br>XM_003450482.5
Clown anemonefish<br>(*Amphiprion ocellaris*)	Chromosome Un	plus	ammecr1><br>XM_023275413.2	tmem164<<br>XM_023275432.2		gabra3<<br>XM_035950591.1	gabrb4><br>XM_023275410.2	vsig1><br>XM_035950595.1	tmlhe<<br>XM_023275436.2		mpp1><br>XM_023275416.2
Humphead wrasse<br>(*Cheilinus undulatus*)	Linkage group 12	plus	ammecr1><br>XM_041800781.1	tmem164<<br>XM_041802168.1		gabrb3<<br>XM_041801835.1	gabrb4><br>XM_041801574.1		<tmlhe<br>XM_041800708.1		>mpp1<br>XM_041801509.1
Ballan wrasse<br>(*Labrus bergylta*)	Chromosome Un	minus	ammecr1><br>XM_020643350.2	tmem164<<br>XM_020643298.2		gabra3<<br>XM_029279224.1	gabrb4><br>XM_020643332.2		tmlhe<<br>XM_029279222.1		mpp1><br>XM_020643347.1
Gilthead seabream<br>(*Sparus aurata*)	Chromosome 13	plus	ammecr1><br>XM_030437616.1	tmem164<<br>XM_030438498.1		gabra3<<br>XM_030438827.1	gabrb4><br>XM_030438052.1	vsig1><br>XM_030436953.1	tmlhe<<br>XM_030436951.1		mpp1><br>XM_030438783.1
Three-spined stickleback<br>(*Gasterosteus aculeatus*)	Chromosome 7	plus	ammecr1><br>XM_040183064.1	tmem164<<br>XM_040183066.1		gabra3<<br>XM_040181782.1	gabrb4><br>XM_040181320.1	vsig1><br>XM_040182500.1	tmlhe<<br>XM_040182498.1		mpp1><br>XM_040182495.1
Ocean sunfish<br>(*Mola mola*)	KV751332.1	plus	ammecr1><br>ENSMMOG00000006683	tmem164<<br>ENSMMOG00000006687		gabra3<<br>ENSMMOG00000006696	gabrb4><br>ENSMMOG00000007817		tmlhe<<br>ENSMMOG00000007820		mpp1><br>ENSMMOG00000007825
Japanese pufferfish<br>(*Takifugu rubripes*)	Chromosome 15	plus	ammecr1><br>XM_029848511.1	tmem164<<br>XM_011611454.2		gabra3<<br>XM_029848706.1	gabrb4><br>XM_029848978.1		tmlhe<<br>XM_003971179.3		mpp1><br>XM_003971089.3
Spotted green pufferfish<br>(*Tetraodon nigroviridis*)	Chromosome 7	minus	ammecr1><br>ENSTNIG00000010785			gabra3<<br>ENSTNIG00000010784	gabrb4><br>ENSTNIG00000010783		tmlhe<<br>ENSTNIG00000010782		mpp1><br>ENSTNIG00000010781

Supplementary Table 6. Analysis of the synteny of *atp4a* in the genomes of ray-finned fishes

Species	Chromosome, scaffold, etc.	direction	Annotated genes in each region								
Spotted gar (*Lepisosteus oculatus*)	Linkage group 24	minus	ncam3< XM_015336471.1	~~~~~~~~~~~~~	sult2st3< XM_006641564.2	~~~~~~~~~~~~~	atp4a> XM_015336490.1	gapdhs> XM_006641527.2	tmem147> XM_006641526.2		
Asian arowana (*Scleropages formosus*)	Chromosome 18	plus			hpn> ENSSFOT00015029597.2		atp4a> ENSSFOT00015029720.2	gapdhs> ENSSFOT00015053639.1	tmem147> ENSSFOT00015029759.2	sult2st3> ENSSFOT00015077427.1	ncam3> ENSSFOT00015029811.2
Golden-line barbell (*Sinocyclocheilus grahami*)	LCYQ01S000585.1	minus						gapdhs> ENSSGRT00000114895.1	tmem147> ENSSGRT00000113073.1	sult2st3> ENSSGRT00000113057.1	ncam3> ENSSGRT00000113055.1
Zebrafish (*Danio rerio*)	Chromosome 16	minus			hpn> ENSDART00000044097.6	fxyd1> ENSDART00000158855.2		gapdhs> ENSDART00000058384.6	tmem147> ENSDART00000058383.5	sult2st3> ENSDART00000037797.7	ncam3> ENSDART00000156921.2
Fathead minnow (*Pimephales promelas*)	Chromosome Un	minus						gapdhs> XM_039650853.1	tmem147> XM_039650849.1	sult2st3> XM_039650848.1	ncam3> XM_039650799.1
Channel catfish (*Ictalurus punctatus*)	Chromosome 23	plus			hpn> ENSIPUT00000010695.1	fxyd1> ENSIPUT00000010780.1	atp4a> ENSIPUT00000010940.1	gapdhs> ENSIPUT00000011051.1			
Mexican tetra (*Astyanax mexicanus*)	APWO02000037.1	plus			hpn> ENSAMXT00000025330.2	fxyd1> ENSAMXT00000057453.1	atp4a> ENSAMXT00000017099.2	gapdhs> ENSAMXT00000017074.2	tmem147> ENSAMXT00000050691.1		
Rainbow trout (*Oncorhynchus mykiss*)	Chromosome 2	plus					atp4a> ENSOMYT00000095336.2	gapdhs> ENSOMYT00000063190.2	tmem147> ENSOMYT00000063286.2	sult2st3> ENSOMYT00000092135.2	ncam3> ENSOMYT00000092085.2
Northern pike (*Esox lucius*)	Linkage group 20	**plus**			hpn> ENSELUT00000034141.2	~~~~~~~~~~~~~	atp4a> ENSELUT00000056849.1	gapdhs> ENSELUT00000086851.1	tmem147> ENSELUT00000042680.2	sult2st3> ENSELUT00000034727.2	ncam3> ENSELUT00000042740.2
Atlantic cod (*Gadus morhua*)	Chromosome 11	minus					atp4a> ENSGMOG00000015889	dennd3< ENSGMOT00000054173.1	slc45a4b< ENSGMOT00000025452.1	gpr20< ENSGMOT00000017588.2	dgat1b< ENSGMOT00000052612.1
Greater amberjack (*Seriola dumerili*)	BDQW01000723.1	plus	steap4< ENSSDUT00000010072.1	glipr2l> ENSSDUT00000010102.1	nacad< ENSSDUT00000010118.1	tbrg4< ENSSDUT00000010141.1	atp4a> ENSSDUT00000010226.1	dennd3< ENSSDUT00000010476.1	slc45a4b< ENSSDUT00000010631.1	gpr20< ENSSDUT00000010665.1	dgat1b< ENSSDUT00000010727.1
Japanese medaka (*Oryzias latipes*)	Chromosome 16	plus	steap4< ENSORLT00000010656.2	glipr2l> ENSORLT00000010668.2	nacad< NSORLT00000034684.1	tbrg4< ENSORLT00000010746.2		dennd3< ENSORLG00000008599	slc45a4b< ENSORLT00000034268.1	gpr20< ENSORLT00000042644.1	dgat1b< ENSORLT00000010827.2
Turquoise killifish (*Nothobranchius furzeri*)	LN600831.1	plus				tbrg4< ENSNFUT00015034056.1			slc45a4b< ENSNFUT00015034189.1		dgat1< ENSNFUT00015036810.1
Platyfish (*Xiphophorus maculatus*)	Chromosome 3	plus	steap4< ENSXMAG00000024455	glipr2l> ENSXMAT00000014467.2	nacad< ENSXMAT00000024795.1	tbrg4< ENSXMAT00000014487.2		dennd3< ENSXMAT00000014496.2	slc45a4b< ENSXMAT00000021069.1	gpr20< ENSXMAT00000014522.2	dgat1b< ENSXMAT00000014535.2
Nile tilapia (*Oreochromis niloticus*)	Linkage group 11	plus	steap4< ENSONIT00000073712.1	glipr2l> ENSONIT00000007516.2	nacad< ENSONIT00000042929.1	tbrg4< ENSONIT00000007524.2	atp4a> ENSONIT00000061964.1	dennd3< ENSONIT00000007554.2	slc45a4b< ENSONIT00000007559.2	gpr20< ENSONIT00000007563.2	dgat1b< ENSONIT00000007567.2
Clown anemonefish (*Amphiprion ocellaris*)	NXFZ01002900.1	plus	steap4< ENSAOCT00000013775.1	glipr2l> ENSAOCT00000013805.1	nacad< ENSAOCT00000013821.1	tbrg4< ENSAOCT00000013832.1	atp4a> ENSAOCT00000013860.1	dennd3< ENSAOCT00000013881.1	slc45a4b< ENSAOCT00000013922.1	gpr20< ENSAOCT00000013936.1	dgat1b< ENSAOCT00000013969.1
Humphead wrasse (*Cheilinus undulatus*)	Linkage group 8	minus	steap4< XM_041792594.1	glipr2l> XM_041794221.1	nacad< XM_041794479.1	tbrg4< XM_041794380.1		dennd3< XM_041792547.1	slc45a4b< XM_041794415.1	gpr20< XM_041793340.1	dgat1b< XM_041794092.1
Ballan wrasse (*Labrus bergylta*)	NW_018114726.1	minus	steap4< XM_020648536.2	glipr2l> XM_020648540.2	nacad< XM_020648539.2	tbrg4< XM_020648494.2		dennd3< XM_020648543.2	slc45a4< ENSLBEG00000023627	gpr20< ENSLBEG00000023607	dgat1b< XM_020648492.2
Gilthead seabream (*Sparus aurata*)	Chromosome 17	plus	steap4< ENSSAUT00010005477.1	glipr2l> ENSSAUT00010005841.1	nacad< ENSSAUT00010005851.1	tbrg4< ENSSAUT00010005870.1	atp4a> ENSSAUT00010010276.1	dennd3< ENSSAUT00010010467.1	slc45a4b< ENSSAUT00010010520.1	gpr20< ENSSAUT00010010556.1	dgat1b< ENSSAUT00010010622.1
Three-spined stickleback (*Gasterosteus aculeatus*)	Group XX	plus	steap4< ENSGACT00000011732.1	glipr2l> ENSGACT00000011757.1	nacad< ENSGACT00000011774.1	tbrg4< ENSGACT00000011784.1	atp4a> ENSGACT00000011799.1	dennd3< ENSGACT00000011815.1	slc45a4b< ENSGACT00000011827.1	gpr20< ENSGACT00000011831.1	dgat1b< ENSGACT00000011865.1
Ocean sunfish (*Mola mola*)	KV751355.1	plus	steap4< ENSMMOG00000002988	glipr2l> ENSMMOG00000003002	nacad< ENSMMOT00000004141.1	tbrg4< ENSMMOG00000003259		dennd3< ENSMMOG00000003273	slc45a4b< ENSMMOG00000003289	gpr20< ENSMMOG00000003298	dgat1b< ENSMMOG00000003317
Japanese pufferfish (*Takifugu rubripes*)	Chromosome 7	plus	steap4< ENSTRUT00000031691.3	glipr2l> ENSTRUT00000054061.2	nacad< ENSTRUT00000049628.2	tbrg4< ENSTRUT00000031164.3		dennd3< ENSTRUT00000072358.1	slc45a4b< ENSTRUT00000030304.3	gpr20< ENSTRUT00000030065.3	dgat1b< ENSTRUT00000081404.1
Spotted green pufferfish (*Tetraodon nigroviridis*)	Chromosome 8	plus	steap4< ENSTNIT00000021796.1	glipr2l> ENSTNIT00000021795.1		tbrg4< ENSTNIT00000021793.1		dennd3< ENSTNIT00000021792.1	slc45a4b< ENSTNIT00000021791.1	gpr20< ENSTNIT00000021790.1	dgat1b< ENSTNIT00000021789.1

Supplementary Table 7. Analysis of the synteny of *atp4b* in the genomes of ray-finned fishes

Species	Chromosome, scaffold, etc.	direction	Annotated genes in each region										
Spotted gar (*Lepisosteus oculatus*)	Linkage group 17	plus			pros1> XM_015364081.1	gas6> XM_006639305.2	tmem255b< XM_015364063.1	grk1a< XM_006639197.2	atp4b> XM_015364080.1	tfdp1b< XM_015364370.1	tmco3< XM_006639302.2	dcun1d2b> XM_006639195.2	adprhl1> XM_015364464.1
Asian arowana (*Scleropages formosus*)	Chromosome 14	minus							atp4b> ENSSFOG00015025471	tfdp1< ENSSFOG00015000387			
Golden-line barbell (*Sinocyclocheilus grahami*)	LCYQ01S001678.1	plus			pros1> ENSSGRT00000051939.1	gas6> ENSSGRT00000051940.1	tmem255b< ENSSGRT00000052034.1	grk1a< ENSSGRT00000052126.1				dcun1d2b> ENSSGRT00000052198.1	adprhl1> ENSSGRT00000052255.1
Zebrafish (*Danio rerio*)	Chromosome 1	minus						grk1a< ENSDART00000081743.6		tfdp1b< ENSDART00000003317.11	tmco3< ENSDART00000109529.4	dcun1d2b> ENSDART00000060971.5	adprhl1> ENSDART00000060968.7
Fathead minnow (*Pimephales promelas*)	Chromosome Un	plus	gas6< XM_039646989.1	pros1< XM_039646987.1	~~~~~~~~~~~~~	tmem255b< XM_039647002.1	~~~~~~~~~~~~~	grk1a< XM_039646990.1		tfdp1b< XM_039646998.1	tmco3< XM_039646985.1	dcun1d2b> XM_039647005.1	adprhl1> XM_039647001.1
Channel catfish (*Ictalurus punctatus*)	Chromosome 26	minus		pros1> XM_017458120.2	gas6> XM_017458107.2	tmem255b< XM_017457018.2	~~~~~~~~~~~~~	grk1a< XM_017456889.2	atp4b> XM_017458202.2	tfdp1b< XM_017458199.2	tmco3< XM_017457316.2	dcun1d2b> XM_047151179.1	adprhl1> XM_017457317.2
Mexican tetra (*Astyanax mexicanus*)	Chromosome 12	minus		pros1> ENSAMXT00000009778.2	gas6> ENSAMXG00000038597	tmem255b< ENSAMXG00000009521	~~~~~~~~~~~~~	grk1a< ENSAMXT00000049362.1	atp4b> ENSAMXT00000016709.2	tfdp1b< ENSAMXT00000045452.1	tmco3< ENSAMXT00000016678.2	adprhl1> ENSAMXT00000041279.1	
Rainbow trout (*Oncorhynchus mykiss*)	Chromosome 18	plus				gas6> ENSOMYG00000055340	tmem255b< ENSOMYT00000164974.1	grk1a< ENSOMYT00000126670.1	atp4b> ENSOMYT00000131416.1	tfdp1< ENSOMYT00000019712.2	tmco3< ENSOMYT00000140488.1	dcun1d2b ENSOMYT00000159352.1	adprhl1> ENSOMYT00000124276.1
Northern pike (*Esox lucius*)	Linkage group 22	minus				gas6> ENSELUT00000036990.2	tmem255b< ENSELUT00000037134.2 ENSELUT00000037160.2	grk1a< ENSELUT00000043576.2	atp4b> ENSELUT00000037191.2	tfdp1< ENSELUT00000043601.2	tmco3< ENSELUT00000037239.2	dcun1d2b> ENSELUT00000043637.2	adprhl1> ENSELUG00000024413
Atlantic cod (*Gadus morhua*)	Chromosome 14	minus				gas6< ENSGMOT00000069138.1		~~~~~~~~~~~~~	atp4b> ENSGMOG00000000187>				
Greater amberjack (*Seriola dumerili*)	BDQW01001299.1	minus			pros1> ENSSDUT00000008337.1	gas6> ENSSDUG00000005990	tmem255b< ENSSDUG00000005950	grk1a< ENSSDUT00000008242.1	atp4b> ENSSDUT00000008198.1		tmco3< ENSSDUT00000008088.1	dcun1d2b> ENSSDUT00000007914.1	adprhl1> ENSSDUT00000007558.1
Japanese medaka (*Oryzias latipes*)	Chromosome 3	minus			pros1> ENSORLT00000006078.2	gas6> ENSORLT00000005988.2	tmem255b< ENSORLT00000005964.2	grk1< ENSORLT00000005943.2			tmco3< ENSORLT00000005931.2	dcun1d2b> ENSORLT00000005914.2	adprhl1> ENSORLT00000005910.2
Turquoise killifish (*Nothobranchius furzeri*)	Chromosome sgr07	minus			pros1> ENSNFUT00015045065.1	gas6> ENSNFUT00015044682.1	tmem255b< ENSNFUT00015044521.1	grk1a< ENSNFUT00015044515.1			tmco3< ENSNFUT00015044465.1	dcun1d2b> ENSNFUG00015020369	adprhl1> ENSNFUT00015044389.1
Platyfish (*Xiphophorus maculatus*)	Chromosome 4	minus			pros1> XM_005806752.3	gas6> XM_005806753.3	tmem255b< XM_023332024.1	grk1a< XM_005806754.2			tmco3< XM_023332602.1	dcun1d2b> XM_023332605.1	adprhl1> XM_023331872.1
Nile tilapia (*Oreochromis niloticus*)	Linkage group 1	minus			pros1> ENSONIT00000081598.1	gas6> ENSONIT00000003964.2	tmem255b< ENSONIG00000003170	grk1a< ENSONIT00000003978.2	atp4b> ENSONIT00000003987.2		tmco3< ENSONIT00000003996.2	dcun1d2b> ENSONIT00000004005.2	adprhl1> ENSONIT00000004007.2
Clown anemonefish (*Amphiprion ocellaris*)	NXFZ01000019.1	minus			pros1> ENSAOCT00000029184.1	gas6> ENSAOCG00000024677	tmem255b< ENSAOCG00000024680	grk1a< ENSAOCT00000029191.1	atp4b> ENSAOCT00000033597.1		tmco3< ENSAOCT00000033602.1	dcun1d2b> ENSAOCT00000029226.1	adprhl1> ENSAOCT00000029230.1
Humphead wrasse (*Cheilinus undulatus*)	Linkage group 1	minus			pros1> XM_041787191.1	gas6> XM_041784646.1	tmem255b< XM_041784660.1	grk1a< XM_041789897.1			tmco3< XM_041785813.1	dcun1d2b> XM_041785859.1	adprhl1> XM_041792895.1
Ballan wrasse (*Labrus bergylta*)	Chromosome Un	minus			pros1> XM_020635922.2	gas6> XM_020635896.2	tmem255b< XM_029277744.1	grk1a< XM_020635913.2			tmco3< XM_029277746.1	dcun1d2b> XM_020635866.2	adprhl1> XM_020635923.2
Gilthead seabream (*Sparus aurata*)	Chromosome 4	minus			pros1> ENSSAUT00010001584.1	gas6> ENSSAUT00010001385.1	tmem255b< ENSSAUG00010000568	grk1a< ENSSAUT00010001079.1	atp4b> ENSSAUT00010001071.1		tmco3< ENSSAUT00010001048.1	dcun1d2b> ENSSAUT00010000972.1	adprhl1> ENSSAUT00010000932.1
Three-spined stickleback (*Gasterosteus aculeatus*)	Group II	plus			pros1> ENSGACT00000020225.1	gas6> ENSGACT00000020234.1	tmem255b< ENSGACT00000020242.1	grk1a< ENSGACT00000020249.1	atp4b> ENSGACT00000020258.1		tmco3< ENSGACT00000020265.1	dcun1d2b> ENSGACT00000020267.1	adprhl1> ENSGACT00000020271.1
Ocean sunfish (*Mola mola*)	KV751315.1	minus			pros1> ENSMMOG00000018080	gas6> ENSMMOG00000018076	tmem255b< ENSMMOG00000018070	grk1a< ENSMMOG00000017962			tmco3< ENSMMOG00000017954	dcun1d2b> ENSMMOG00000017949	adprhl1> ENSMMOG00000017939
Japanese pufferfish (*Takifugu rubripes*)	Chromosome 13	minus			pros1> ENSTRUT00000044270.3	gas6> ENSTRUT00000088266.1	tmem255b< ENSTRUT00000044267.3	grk1a< ENSTRUT00000044264.3			tmco3< ENSTRUT00000080273.1	dcun1d2b> ENSTRUT00000072896.1	adprhl1> ENSTRUT00000044261.3
Spotted green pufferfish (*Tetraodon nigroviridis*)	Chromosome 5	plus			pros1> ENSTNIT00000003330.1	gas6> ENSTNIG00000009449	tmem255b< ENSTNIG00000009450	grk1a< ENSTNIT00000012514.1			tmco3< ENSTNIT00000012515.1	dcun1d2b> ENSTNIT00000012516.1	adprhl1> ENSTNIT00000003735.1

Supplementary Table 8. Analysis of the synteny of *pgc* in the genomes of ray-finned fishes

Species	Chromosome, scaffold, etc.	direction	Annotated genes in each region											
Spotted gar<br>(*Lepisosteus oculatus*)	Linkage group 3	plus						pgc><br>ENSLOCG00000012718							
Asian arowana<br>(*Scleropages formosus*)	Primary assembly 24	plus	rabl2><br>ENSSFOT00015032790.2	shank3a<<br>ENSSFOT00015032816.2		arsa><br>ENSSFOT00015051403.1	mapk8ip2<<br>ENSSFOT00015032950.2	pgc><br>ENSSFOT00015033168.2	pgc><br>ENSSFOT00015045425.1	pgc><br>ENSSFOT00015033319.2	pgc><br>ENSSFOT00015033344.2	chkb><br>ENSSFOT00015033469.2	Kbtbd11-like><br>ENSSFOG00015021147	cpt1b><br>ENSSFOT00015041780.1	sephs1<<br>ENSSFOT00015000233.2
Golden-line barbell<br>(*Sinocyclocheilus grahami*)	LCYQ01S000022.1	minus	rabl2><br>ENSSGRT00000045087.1	SHANK3<<br>ENSSGRT00000045084.1		arsa><br>ENSSGRT00000045081.1	mapk8ip2<<br>ENSSGRT00000045067.1					chkb><br>ENSSGRT00000044918.1		cpt1b><br>ENSSGRT00000042298.1	sephs1<<br>ENSSGRT00000032804.1
Zebrafish<br>(*Danio rerio*)	Primary assembly 18	plus	rabl2><br>ENSDART00000092416.6	shank3a<<br>ENSDART00000091805.6		arsa><br>ENSDART00000140193.2	mapk8ip2<<br>ENSDART00000092033.6					chkb><br>ENSDART00000092053.6	Kbtbd13-like><br>ENSDARG00000113137	cpt1b><br>ENSDART00000081132.6	sephs1<<br>ENSDART00000081143.6
Fathead minnow<br>(*Pimephales promelas*)	NW_024121599.1	plus		shank3a<<br>XM_039650534.1		arsa><br>XM_039650538.1	mapk8ip2<<br>XM_039650509.1					chkb><br>XM_039650542.1	kelch-like protein diablo><br>XM_039650535.1	cpt1b><br>XP_039506465.1	sephs1<<br>XM_039650543.1
Channel catfish<br>(*Ictalurus punctatus*)	Primary assembly 4	minus	rabl2><br>ENSIPUT00000012526.1	shank3a<<br>ENSIPUT00000012318.1		arsa><br>ENSIPUT00000012089.1	mapk8ip2<<br>ENSIPUT00000011941.1	pgc><br>ENSIPUT00000011987.1				chkb><br>ENSIPUT00000011866.1	kelch-like protein 32><br>ENSIPUG00000007695	cpt1b><br>ENSIPUT00000011682.1	sephs1<<br>ENSIPUT00000011578.1
Mexican tetra<br>(*Astyanax mexicanus*)	Primary assembly 18	plus				arsa><br>ENSAMXT00000013333.2	mapk8ip2<br>ENSAMXT00000013317.2	pgc><br>ENSAMXG00000012956				chkb><br>ENSAMXT00000034789.1	kelch-like protein 32><br>ENSAMXG00000040181	cpt1b><br>ENSAMXT00000013276.2	sephs1<<br>ENSAMXT00000013229.2
Rainbow trout<br>(*Oncorhynchus mykiss*)	Primary assembly 2	minus	rabl2><br>ENSOMYT00000075675.2	shank3a<<br>ENSOMYT00000157193.1		arsa><br>ENSOMYT00000075507.2	mapk8ip2<<br>ENSOMYT00000075455.2	pgc><br>ENSOMYT00000075432.2		zp3-like<<br>ENSOMYT00000075330.2	zp4-like><br>ENSOMYT00000075298.2	chkb><br>ENSOMYT00000124524.1		cpt1b><br>ENSOMYT00000074047.2	sephs1<<br>ENSOMYT00000070616.2
Northern pike<br>(*Esox lucius*)	Linkage group 19	plus	rabl2><br>ENSELUT00000035234.2	shank3a<<br>ENSELUT00000035039.2	pgc><br>ENSELUT00000034941.2	arsa><br>ENSELUT00000064842.1	mapk8ip2<<br>ENSELUT00000034886.2	pgc><br>ENSELUT00000034872.2		zp3<<br>ENSELUG00000022451		chkb><br>ENSELUT00000034769.2		cpt1b><br>ENSELUT00000042715.2	sephs1<<br>ENSELUT00000034643.2
Atlantic cod<br>(*Gadus morhua*)	Primary assembly 9	plus				arsa><br>ENSGMOG00000012264	mapk8ip2<<br>ENSGMOG00000012254	~~~~~~~~~~~~~	pgc><br>ENSGMOG00000012250	zp3<<br>ENSGMOG00000012244	zp4-like><br>ENSGMOG00000012239	chkb><br>ENSGMOG00000012235	kelch-like protein42><br>ENSGMOG00000026995	cpt1b><br>ENSGMOG00000012229	sephs1<<br>ENSGMOG00000012224
Greater amberjack<br>(*Seriola dumerili*)	BDQW01000219.1	plus	rabl2><br>ENSSDUT00000020665.1	shank3a<<br>ENSSDUT00000020896.1		arsa><br>ENSSDUT00000020965.1	mapk8ip2<<br>ENSSDUT00000021019.1	pgc><br>ENSSDUG00000015047		zp3<<br>ENSSDUG00000015083	zp4-like><br>ENSSDUG00000015118	chkb><br>ENSSDUT00000021331.1	actin-binding protein IPP-like><br>ENSSDUG00000015268	cpt1b><br>ENSSDUT00000021434.1	sephs1<<br>ENSSDUT00000021502.1
Japanese medaka<br>(*Oryzias latipes*)	Primary assembly 6	plus	rabl2><br>ENSORLT00000012389.2	shank3a<<br>ENSORLT00000046372.1		arsa><br>ENSORLT00000012547.2	mapk8ip2<<br>ENSORLT00000034690.1			zp3-like<<br>ENSORLG00000010134	zp4-like><br>ENSORLG00000010086	chkb><br>ENSORLT00000012739.2		cpt1b><br>ENSORLT00000012821.2	sephs1<<br>ENSORLT00000012854.2
Turquoise killifish<br>(*Nothobranchius furzeri*)	Chromosome sgr13	plus	rabl2><br>ENSNFUT00015040492.1	shank3a<<br>ENSNFUT00015040501.1		arsa><br>ENSNFUT00015040513.1	MAPK8IP2<<br>ENSNFUT00015040561.1			zp3-like<<br>ENSNFUG00015018748	zp4-like><br>ENSNFUG00015018751	chkb><br>ENSNFUT00015040635.1		cpt1b><br>ENSNFUT00015040942.1	sephs1<<br>ENSNFUT00015040950.1
Platyfish<br>(*Xiphophorus maculatus*)	Primary assembly 2	plus	rabl2><br>ENSXMAT00000004584.2	shank3a<<br>ENSXMAT00000037697.1		arsa><br>ENSXMAT00000004635.2	mapk8ip2<<br>ENSXMAT00000004650.2			zp3.2<<br>ENSXMAT00000004657.2		chkb><br>ENSXMAT00000033161.1		cpt1b><br>ENSXMAT00000004684.2	sephs1<<br>ENSXMAT00000004697.2
Nile tilapia<br>(*Oreochromis niloticus*)	Linkage group 7	plus	rabl2><br>ENSONIT00000018753.2	shank3a<<br>ENSONIT00000057196.1		arsa><br>ENSONIT00000018746.2	mapk8ip2<<br>ENSONIT00000092349.1	pgc><br>ENSONIG00000031098		zp3<<br>ENSONIG00000014877	zp4-like><br>ENSONIT00000054322.1	chkb><br>ENSONIT00000018739.2	kelch-like protein 42><br>ENSONIT00000093589.1	cpt1b><br>ENSONIT00000087621.1	sephs1<<br>ENSONIT00000018726.2
Clown anemonefish<br>(*Amphiprion ocellaris*)	Chromosome 3	plus	rabl2<<br>XM_023281333.3	~~~~~~~~~~~~~	shank3a<<br>XM_055009172.1	arsa><br>XM_023293179.3	mapk8ip2<<br>XM_023293177.3	pgc><br>XM_023292203.3		zp3-like<<br>XM_023292194.2	zp4-like><br>XM_023292193.3	chkb><br>XM_023292198.3	kelch-like protein 38><br>XM_023292191.3	cpt1b><br>XM_023292207.3	sephs1<<br>XM_023292195.3
Humphead wrasse<br>(*Cheilinus undulatus*)	Linkage group 9	minus		shank3a<<br>XM_041794579.1		arsa><br>XM_041795570.1	mapk8ip2<<br>XM_041795569.1			zp3-like<<br>XM_041795381.1	zp4-like><br>XM_041794698.1	chkb><br>XM_041796526.1	kelch-like protein 42><br>XM_041796262.1	cpt1b><br>XM_041796262.1	sephs1<<br>XM_041796263.1
Ballan wrasse<br>(*Labrus bergylta*)	NW_018115502.1<br>NW_018115321.1	minus				arsa><br>XM_020658657.2	mapk8ip2<<br>XM_020658655.2	~~~~~~~~~~~~~		zp3-like<<br>XM_020657774.2		chkb><br>XM_020657773.2	A-kelch domain-containing protein 7A-like><br>XM_029282125.1	cpt1b><br>XM_020657770.2	sephs1<<br>XM_029282124.1
Gilthead seabream<br>(*Sparus aurata*)	Primary assembly 8	minus	rabl2><br>ENSSAUG00010024193	shank3a<<br>ENSSAUT00010062414.1		arsa><br>ENSSAUT00010062391.1	mapk8ip2<<br>ENSSAUT00010062281.1	pgc><br>ENSSAUG00010024135		zp3-like<<br>ENSSAUT00010062268.1	zp4-like><br>ENSSAUT00010062254.1	chkb><br>ENSSAUT00010062236.1	kelch-like protein 42><br>ENSSAUG00010024107	cpt1b><br>ENSSAUT00010061903.1	sephs1<<br>ENSSAUT00010061686.1
Three-spined stickleback<br>(*Gasterosteus aculeatus*)	Group XIX	minus	rabl2><br>ENSGACG00000012474	shank3a<<br>ENSGACG00000012458	~~~~~~~~~~~~~	arsa><br>ENSGACG00000012412	mapk8ip2<<br>ENSGACG00000012408	pgc><br>ENSGACT00000016428.1		zp3-like<<br>ENSGACT00000016394.1	zp4-like><br>ENSGACT00000016383.1	chkb><br>ENSGACT00000016368.1<br>Exons>		cpt1b><br>ENSGACT00000016348.1	sephs1<<br>ENSGACT00000016294.1
Ocean sunfish<br>(*Mola mola*)	KV751367.1	plus				arsa><br>ENSMMOG00000012978	mapk8ip2<<br>ENSMMOG00000012984			zp3-like<<br>ENSMMOG00000012990	zp4-like><br>ENSMMOT00000017332.1	chkb><br>ENSMMOG00000012997		cpt1b><br>ENSMMOG00000013001	sephs1<<br>ENSMMOG00000013005
Japanese pufferfish<br>(*Takifugu rubripes*)	Primary assembly 9	plus	rabl2><br>ENSTRUT00000064723.1	shank3a<<br>ENSTRUT00000089753.1		arsa><br>ENSTRUT00000031028.3	mapk8ip2<<br>ENSTRUT00000030601.3			zp3a.2<<br>ENSTRUT00000088003.1	zp4-like><br>ENSTRUG00000011883	chkb><br>ENSTRUT00000054726.2		cpt1b><br>ENSTRUT00000029412.3	sephs1<<br>ENSTRUT00000048952.2
Spotted green pufferfish<br>(*Tetraodon nigroviridis*)	Chromosome 13	plus	rabl2><br>ENSTNIT00000021346.1	shank3a<<br>ENSTNIT00000021344.1		arsa><br>ENSTNIT00000021343.1	mapk8ip2<<br>ENSTNIT00000021342.1			zp3.2<<br>ENSTNIT00000021341.1	zp4-like><br>ENSTNIT00000000148.1	chkb><br>ENSTNIT00000021340.1		cpt1b><br>ENSTNIT00000021339.1	sephs1<<br>ENSTNIT00000021338.1

Supplementary Table 9. Analysis of the synteny of *pga1* in the genomes of ray-finned fishes

Species	Chromosome, scaffold, etc.	direction	Annotated genes in each region											
Spotted gar (*Lepisosteus oculatus*)	Linkage group 10	minus				pkd1b> ENSLOCT00000013178.1				spata20> ENSLOCT00000013157.1	cacna1g> ENSLOCT00000013133.1			
Asian arowana (*Scleropages formosus*)	Primary assembly 20	plus		galk1< ENSSFOT00015063206.1		pkd1b> ENSSFOT00015002459.2	pga1> ENSSFOT00015003890.2			spata20> ENSSFOT00015073101.1	cacna1g> ENSSFOT00015069606.1	~~~~~~~~~~~~~	urotensin-2 receptor> ENSSFOG00015002675	
Golden-line barbell (*Sinocyclocheilus grahami*)	LCYQ01S000573.1	minus	dhx8< ENSSGRG00000033886							spata20> ENSSGRG00000033917	cacna1g> ENSSGRG00000033956 ENSSGRG00000033959			
Zebrafish (*Danio rerio*)	Chromosome 12	plus	dhx8< ENSDART00000066282.5							spata20> ENSDART00000066288.6	cacna1g> ENSDART00000153256.3		urotensin-2 receptor> ENSDARG00000009624	urotensin-2 receptor> ENSDARG00000115189
Fathead minnow (*Pimephales promelas*)	Chromosome Un	plus	dhx8< XM_039664876.1							spata20> XM_039664877.1	cacna1g> XM_039664889.1		urotensin-2 receptor> XM_039664891.1	
Channel catfish (*Ictalurus punctatus*)	Primary assembly 13	minus				pkd1b> ENSIPUT00000005616.1	pga1> ENSIPUG00000003636	pga1> ENSIPUG00000003625						
Mexican tetra (*Astyanax mexicanus*)	Primary assembly 4	minus	dhx8< ENSAMXT00000047527.1							spata20> ENSAMXT00000009728.2	cacna1g> ENSAMXG00000029856	~~~~~~~~~~~~~	urotensin-2 receptor> ENSAMXG00000035321	urotensin-2 receptor> ENSAMXG00000025203
Rainbow trout (*Oncorhynchus mykiss*)	Primary assembly 16	minus		galk1< ENSOMYT00000146450.1			pga1> ENSOMYT00000053273.2	pga1> ENSOMYT00000119326.1			cacna1g> ENSOMYT00000160756.1		urotensin-2 receptor> ENSOMYT00000053164.2	urotensin-2 receptor> ENSOMYT00000053097.2
Northern pike (*Esox lucius*)	Linkage group 05	plus		galk1< ENSELUT00000036809.2		pkd1b> ENSELUT00000036659.2	pga1> ENSELUG00000023745			spata20> ENSELUT00000036631.2	cacna1g> ENSELUT00000036440.2		urotensin-2 receptor> ENSELUG00000023587	
Atlantic cod (*Gadus morhua*)	Primary assembly 18	plus					pga1> ENSGMOG00000013247>	PI-PLC X domain-containing protein 1-like ENSGMOG00000036083>		spata20> ENSGMOG00000004610				
Greater amberjack (*Seriola dumerili*)	BDQW01000068.1	minus			asb12a< ENSSDUT00000013993.1	~~~~~~~~~~~~~	pga1> ENSSDUG00000009527			spata20> ENSSDUT00000013228.1	cacna1g> ENSSDUT00000013196.1			
Japanese medaka (*Oryzias latipes*)	Primary assembly 19	plus											urotensin-2 receptor> ENSORLG00000020371	urotensin-2 receptor> ENSORLG00000029308
Turquoise killifish (*Nothobranchius furzeri*)	Chromosome sgr12	plus			asb12a< ENSNFUT00015034136.1	pkd1b> ENSNFUT00015034162.1		PI-PLC X domain-containing protein 1-like> ENSNFUG00015016041	~~~~~~~~~~~~~	spata20> ENSNFUT00015036228.1	cacna1g> ENSNFUT00015044733.1	~~~~~~~~~~~~~	urotensin-2 receptor> ENSNFUG00015021587	
Platyfish (*Xiphophorus maculatus*)	Primary assembly 10	minus			asb12a< ENSXMAT00000017256.2			PI-PLC X domain-containing protein 1-like> ENSXMAG00000023157		spata20> ENSXMAT00000017240.2	cacna1g> ENSXMAT00000037354.1	~~~~~~~~~~~~~	urotensin-2 receptor> ENSXMAG00000025211	urotensin-2 receptor< ENSXMAG00000012752
Nile tilapia (*Oreochromis niloticus*)	Linkage group 8	minus		asb12a< ENSONIT00000024753.2	pkd1> ENSONIT00000088162.1	~~~~~~~~~~~~~	pga1> ENSONIT00000024759.2	pga1> ENSONIG00000038250	PI-PLC X domain-containing protein 1> ENSONIG00000019653	spata20> ENSONIT00000054232.1	cacna1g> ENSONIT00000039496.1		urotensin-2 receptor< ENSONIG00000021202	urotensin-2 receptor< ENSONIT00000038127.1
Clown anemonefish (*Amphiprion ocellaris*)	Chromosome 18	plus			asb12a< XM_023274876.3	pkd1b> XM_055004469.1		PI-PLC X domain-containing protein 1-like> ENSAOCG00000016748		spata20> ENSAOCT00000030605.1	cacna1g> XM_055004159.1			
Humphead wrasse (*Cheilinus undulatus*)	Linkage group 20	minus			asb12-like< XM_041815887.1	pkd1b> XM_041816038.1				spata20> XM_041814853.1	cacna1g> XM_041814823.1		urotensin-2 receptor> XM_041815576.1	urotensin-2 receptor> XM_041816416.1
Ballan wrasse (*Labrus bergylta*)	FKLU01000357.1	plus			asb12< ENSLBET00000000789.1	pkd1b< ENSLBET00000001147.1		PI-PLC X domain-containing protein 1-like> ENSLBEG00000001009		spata20> ENSLBET00000001554.1	cacna1g> ENSLBEG00000001196			
Gilthead seabream (*Sparus aurata*)	Primary assembly 20	minus			asb12a< ENSSAUT00010062305.1	pkd1b> ENSSAUT00010062303.1	pga1> ENSSAUG00010024124	pga1< ENSSAUG00010024121	pga1> ENSSAUG00010024080	spata20> ENSSAUT00010061894.1	cacna1g> ENSSAUT00010060036.1		urotensin-2 receptor> ENSSAUG00010020830	
Three-spined stickleback (*Gasterosteus aculeatus*)	Group V	minus			asb12a< ENSGACT00000007219.1	~~~~~~~~~~~~~	pga1> ENSGACT00000007199.1	PI-PLC X domain-containing protein 1-like> ENSGACT00000066168.1		spata20> ENSGACT00000007101.1	cacna1g> ENSGACT00000007069.2		urotensin-2 receptor> ENSGACT00000007048.1	
Ocean sunfish (*Mola mola*)	KV751339.1	plus			asb12a< ENSMMOG00000004779	~~~~~~~~~~~~~	pga1> ENSMMOG00000004783	PI-PLC X domain-containing protein 1-like> ENSMMOG00000004786		spata20> ENSMMOG00000004797	cacna1g> ENSMMOG00000004805			urotensin-2 receptor< ENSMMOG00000004812
Japanese pufferfish (*Takifugu rubripes*)	Primary assembly 1	minus			asb12a< ENSTRUT00000083415.1	pkd1b> ENSTRUG00000024317	pga1> ENSTRUT00000044156.3	PI-PLC X domain-containing protein 1-like> ENSTRUG00000029217		spata20> ENSTRUT00000044169.3	cacna1g> ENSTRUT00000062651.1			
Spotted green pufferfish (*Tetraodon nigroviridis*)	Chromosome 2	minus			asb12a< ENSTNIT00000007255.1	pkd1b> ENSTNIT00000007254.1	pga1> ENSTNIG00000004456			spata20> ENSTNIT00000007252.1	cacna1g> ENSTNIT00000009834.1			

Supplementary Table 10. Analysis of the synteny of *pga2* and *pga3* in the genomes of ray-finned fishes

Species	Chromosome, scaffold, etc.	direction	Annotated genes in each region																											
Spotted gar (*Lepisosteus oculatus*)	Linkage group 3	plus						slc6a17<<br>ENSLOCT00000014396.1		kcnc4<<br>ENSLOCG00000011699		rbm15><br>ENSLOCT00000022082.1	slc16a4<<br>ENSLOCT00000014417.1		lamtor5<<br>ENSLOCT00000014426.1	prok1><br>ENSLOCT00000014432.1	pga>>>><br>ENSLOCG00000011725<br>ENSLOCG00000011734<br>ENSLOCG00000011744<br>ENSLOCG00000011751		~~~~~~~~~~~~~~								amigo1<<br>XM_015342558.1	>gpr61<br>XM_015342426.1	~~~~~~~~~~~~~~	ampd2><br>XM_015342656.1
Asian arowana (*Scleropages formosus*)	Chromosome 25	plus						kcnc4><br>XM_018741802.2				rbm15><br>XM_029249388.1	slc16a4<<br>XM_018741811.2	~~~~~~~~~~~~~~	lamtor5<<br>XM_018741939.2															
Asian arowana (*Scleropages formosus*)	Primary assembly 16	plus								slc6a17<<br>ENSSFOT00015030453.2							pga3>>><br>ENSSFOG00015026879<br>ENSSFOG00015031053<br>ENSSFOG00015030882								~~~~~~~~~~~~~~	amigo1><br>ENSSFOT00015031479.2		~~~~~~~~~~~~~~	ampd2<<br>ENSSFOG00015019994
Golden-line barbell (*Sinocyclocheilus grahami*)	LCYQ01S000399.1	minus	ampd2b<<br>ENSSGRG00000007068	~~~~~~~~~~~~~~	gpr61<<br>ENSSGRT00000010448.1	amigo1><br>ENSSGRT00000010444.1	~~~~~~~~~~~~~~	kcnc4><br>ENSSGRT00000003486.1		slc6a17<<br>ENSSGRT00000003284.1	~~~~~~~~~~~~~~	rbm15><br>ENSSGRT00000002129.1	slc16a4<<br>ENSSGRT00000107667.1		lamtor5<<br>ENSSGRT00000107666.1	prok1><br>ENSSGRT00000107664.1			znf335<<br>ENSSGRG00000050358	mmp9<<br>ENSSGRT00000107589.1	~~~~~~~~~~~~~~	adora1b><br>ENSSGRT00000107500.1	dclre1b><br>ENSSGRT00000107499.1							
Zebrafish (*Danio rerio*)	Chromosome 8	minus	ampd2b<<br>XM_021478444.1	~~~~~~~~~~~~~~	gpr61<<br>XM_005167113.4	amigo1><br>XM_001923988.7	~~~~~~~~~~~~~~	kcnc4><br>NM_001195196.1		slc6a17<<br>ENSDART00000156570.4	~~~~~~~~~~~~~~	rbm15><br>ENSDART00000111617.3	slc16a4<<br>ENSDART00000078656.4		lamtor5<<br>ENSDART00000149746.2	prok1><br>ENSDART00000113197.3			znf335<<br>ENSDART00000172002.2	mmp9<<br>ENSDART00000062845.5	~~~~~~~~~~~~~~	adora1b><br>ENSDART00000112926.4	dclre1b><br>ENSDART00000099692.4							
Fathead minnow (*Pimephales promelas*)	Chromosome Un	minus	ampd2b<<br>XM_039657836.1	~~~~~~~~~~~~~~	gpr61<<br>XM_039657881.1	amigo1><br>XM_039657878.1	~~~~~~~~~~~~~~	kcnc4><br>XM_039657871.1		slc6a17<<br>XM_039658117.1	~~~~~~~~~~~~~~	rbm15><br>XM_039657830.1	slc16a4<<br>XM_039658006.1		lamtor5<<br>XM_039658008.1	prok1><br>XM_039658007.1			znf335<<br>XM_039657804.1	mmp9<<br>XM_039658047.1	~~~~~~~~~~~~~~	adora1b><br>XM_039658005.1	dclre1b><br>XM_039658002.1							
Channel catfish (*Ictalurus punctatus*)	Primary assembly 5	minus	ampd2b<<br>XM_017468391.3	~~~~~~~~~~~~~~	gpr61<<br>XM_017467201.2	amigo1><br>XM_017467064.3	~~~~~~~~~~~~~~	kcnc4><br>XM_017467045.3		slc6a17<<br>XM_047155668.2	~~~~~~~~~~~~~~	rbm15><br>ENSIPUT00000006635.1	slc16a4<<br>ENSIPUT00000006646.1	~~~~~~~~~~~~~~	lamtor5<<br>ENSIPUT00000006704.1	prok1><br>ENSIPUT00000006742.1	pga2>>><br>ENSIPUT00000006788.1<br>ENSIPUG00000004526<br>ENSIPUT00000006856.1		znf335<<br>ENSIPUT00000006874.1	mmp9<<br>ENSIPUT00000007028.1										
Mexican tetra (*Astyanax mexicanus*)	Primary assembly 6	plus	ampd2b<<br>XM_049485478.1	~~~~~~~~~~~~~~	gpr61<<br>XM_049485947.1	amigo1><br>XM_022685673.2	~~~~~~~~~~~~~~	kcnc4><br>XM_007232973.4		slc6a17<<br>XM_007232972.4	~~~~~~~~~~~~~~	rbm15><br>ENSAMXT00000026829.2	slc16a4<<br>ENSAMXT00000007965.2		lamtor5<<br>ENSAMXT00000007961.2	prok1><br>ENSAMXT00000046409.1	pga2>><br>ENSAMXT00000007956.2<br>ENSAMXT00000007955.2		znf335<<br>ENSAMXT00000007954.2	mmp9<<<br>ENSAMXT00000050403.1<br>ENSAMXT00000007936.2	~~~~~~~~~~~~~~	adora1b><br>ENSAMXT00000007930.2	dclre1b><br>ENSAMXT00000007928.2							
Rainbow trout (*Oncorhynchus mykiss*)	Primary assembly 9	minus	ampd2b<<br>ENSOMYG00000009071	~~~~~~~~~~~~~~	gpr61<<br>ENSOMYT00000020013.2		~~~~~~~~~~~~~~	kcnc4><br>XM_036988924.1	~~~~~~~~~~~~~~	slc6a17<<br>ENSOMYT00000023749.2	~~~~~~~~~~~~~~	rbm15><br>ENSOMYT00000018985.2	slc16a4<<br>ENSOMYT00000018971.2		lamtor5<<br>ENSOMYT00000018854.2	prok1><br>ENSOMYT00000142282.1	pga2><br>ENSOMYT00000018762.2		znf335<<br>ENSOMYG00000008004	mmp9<<br>ENSOMYT00000154708.1										
Rainbow trout (*Oncorhynchus mykiss*)	Primary assembly 7	plus						kcnc4><br>ENSOMYG00000003774		slc6a17<<br>ENSOMYG00000003804							pga3><br>ENSOMYG00000003845										amigo1<<br>ENSOMYT00000008368.2	gpr61><br>ENSOMYT00000008406.2	~~~~~~~~~~~~~~	ampd2b><br>ENSOMYG00000004549
Northern pike (*Esox lucius*)	Chromosome 12	minus	ampd2b<<br>XM_029123869.2	~~~~~~~~~~~~~~	gpr61<<br>XM_020051444.2	amigo1<<br>XM_020051457.2	~~~~~~~~~~~~~~	kcnc4><br>ENSELUG00000016204		slc6a17<<br>ENSELUT00000081850.1	~~~~~~~~~~~~~~	rbm15><br>ENSELUT00000025572.2	slc16a4<<br>ENSELUT00000025537.2		lamtor5<<br>ENSELUT00000025496.2	prok1><br>ENSELUT00000086554.1	pga2>><><br>ENSELUG00000015905<br>ENSELUG00000016001<br>ENSELUG00000015670<br>ENSELUT00000023845.2		znf335<<br>ENSELUT00000023805.2	mmp9<<br>ENSELUT00000023147.2		adora1b<<br>ENSELUT00000023029.2	dclre1b><br>ENSELUT00000022976.2							
Atlantic cod (*Gadus morhua*)	Primary assembly 1	minus	ampd2b<<br>XM_030368407.1	~~~~~~~~~~~~~~	gpr61<<br>XM_030368613.1	amigo1><br>XM_030368592.1	~~~~~~~~~~~~~~	kcnc4><br>XM_030368542.1		slc6a17<<br>ENSGMOT00000001391.2	~~~~~~~~~~~~~~	rbm15><br>ENSGMOT00000021946.2	slc16a4<<br>ENSGMOT00000001416.2		lamtor5<<br>ENSGMOT00000060012.1	prok1><br>ENSGMOT00000024685.1	pga2>>><br>ENSGMOG00000027348<br>ENSGMOG00000001326<br>ENSGMOG00000027348		znf335<<br>ENSGMOG00000001344	dclre1b><br>ENSGMOT00000039429.1	mmp9<<br>ENSGMOT00000047630.1	adora1b<<br>ENSGMOG00000030635								
Atlantic cod (*Gadus morhua*)	Chromosome 13	minus								slc6a17<<br>XM_030374831.1	~~~~~~~~~~~~~~						pga3><br>XM_030374835.1										amigo1 <<br>XM_030375860.1	gpr61><br>XM_030373632.1		ampd2b><br>XP_030229778.1
Greater amberjack (*Seriola dumerili*)	BDQW01000056.1	plus	ampd2b<<br>ENSSDUG00000008395	~~~~~~~~~~~~~~	gpr61<<br>ENSSDUG00000008484	amigo1><br>ENSSDUG00000008496	~~~~~~~~~~~~~~	kcnc4><br>ENSSDUT00000012176.1		slc6a17<<br>ENSSDUT00000012210.1	~~~~~~~~~~~~~~	rbm15><br>ENSSDUT00000012268.1	slc16a4<<br>ENSSDUT00000012279.1		lamtor5<<br>ENSSDUT00000012392.1	prok1><br>ENSSDUT00000012418.1	pga2><br>ENSSDUG00000008954		znf335<<br>ENSSDUT00000013961.1	mmp9<<br>ENSSDUT00000014058.1		adora1<<br>ENSSDUT00000014094.1	dclre1b><br>ENSSDUT00000014110.1							
Greater amberjack (*Seriola dumerili*)	GCF_002260705.1	plus								slc6a17<<br>XM_022745678.1							pga3><br>XM_022745658.1										amigo1 <<br>XM_022745699.1	gpr61><br>XM_022745696.1		ampd2b><br>XM_022745625.1
Japanese medaka (*Oryzias latipes*)	Primary assembly 7	plus	ampd2b<<br>XM_011477008.3	~~~~~~~~~~~~~~	gpr61<<br>XM_011477012.3	amigo1><br>XM_004070675.4	~~~~~~~~~~~~~~	kcnc4><br>ENSORLG00000006154		slc6a17<<br>ENSORLT00000007777.2	~~~~~~~~~~~~~~	rbm15><br>ENSORLT00000007793.2	slc16a4<<br>ENSORLT00000007854.2		lamtor5<<br>ENSORLT00000007863.2	prok1><br>ENSORLT00000036139.1			znf335<<br>ENSORLT00000027498.1	mmp9<<br>ENSORLT00000007968.2		adora1<<br>ENSORLG00000006349	dclre1b><br>ENSORLT00000007986.2							
Turquoise killifish (*Nothobranchius furzeri*)	Primary assembly sgr15	plus	ampd2b<<br>XM_054745848.1	~~~~~~~~~~~~~~	gpr61<<br>XM_054745854.1	amigo1><br>XM_015967720.2	~~~~~~~~~~~~~~	kcnc4><br>ENSNFUG00015022655		slc6a17<<br>ENSNFUT00015050173.1	~~~~~~~~~~~~~~	rbm15><br>ENSNFUT00015050198.1	slc16a4<<br>ENSNFUT00015050206.1		lamtor5<<br>ENSNFUT00015050215.1	prok1><br>ENSNFUT00015050224.1		~~~~~~~~~~~~~~	znf335<<br>ENSNFUT00015050473.1	mmp9<<br>ENSNFUT00015050816.1		adora1<<br>ENSNFUT00015050899.1	~~~~~~~~~~~~~~	dclre1b><br>ENSNFUT00015050907.1						
Platyfish (*Xiphophorus maculatus*)	Primary assembly 1	plus	ampd2b<<br>XM_005801331.3	~~~~~~~~~~~~~~	gpr61<<br>XM_023352643.1	amigo1><br>XM_005801335.2	~~~~~~~~~~~~~~	kcnc4><br>XM_014469650.2		slc6a17<<br>ENSXMAT00000010146.2	~~~~~~~~~~~~~~	rbm15><br>ENSXMAT00000020267.2	slc16a4<<br>ENSXMAT00000010157.2		lamtor5<<br>ENSXMAT00000021214.1	prok1><br>ENSXMAT00000040901.1			znf335<<br>ENSXMAT00000033392.1	mmp9<<br>ENSXMAT00000024157.1		adora1<<br>ENSXMAT00000010234.2	dclre1b><br>ENSXMAT00000041264.1							
Nile tilapia (*Oreochromis niloticus*)	Linkage group 20	minus	ampd2b<<br>XM_003444921.5	~~~~~~~~~~~~~~	gpr61<<br>XM_005478164.3	amigo1><br>XM_003444923.5	~~~~~~~~~~~~~~	kcnc4><br>ENSONIT00000003159.2		slc6a17><br>ENSONIT00000077835.1	~~~~~~~~~~~~~~	rbm15><br>ENSONIT00000003171.2	slc16a4<<br>ENSONIT00000003172.2		lamtor5<<br>ENSONIT00000003180.2	prok1><br>ENSONIT00000063504.1	pga2><br>ENSONIG00000002541		znf335<<br>ENSONIT00000041704.1	mmp9<<br>ENSONIT00000054292.1		adora1<<br>ENSONIT00000065616.1	dclre1b><br>ENSONIT00000036162.1							
Clown anemonefish (*Amphiprion ocellaris*)	Chromosome 8	plus								slc6a17<<br>XM_023280221.3	~~~~~~~~~~~~~~	rbm15><br>XM_023292512.3	slc16a4<<br>XM_023292517.3		lamtor5<<br>XM_023292514.3	prok1><br>XM_023292521.3	pga2><br>XM_023292508.3	~~~~~~~~~~~~~~	znf335<<br>XM_023292509.3	mmp9<<br>XM_023292507.3		adora1<<br>XM_023292513.3	dclre1b><br>XM_023285846.3	~~~~~~~~~~~~~~	kcnc4><br>XM_023289725.3	~~~~~~~~~~~~~~	amigo1<<br>XM_023289721.3	gpr61><br>XM_055012973.1	~~~~~~~~~~~~~~	ampd2b><br>XM_035954281.2
Clown anemonefish (*Amphiprion ocellaris*)	NXFZ01003055.1	minus															pga3><br>ENSAOCG00000000130										amigo1<<br>ENSAOCT00000013694.1	gpr61><br>ENSAOCT00000013684.1		ampd2a><br>ENSAOCT00000013638.1
Humphead wrasse (*Cheilinus undulatus*)	Chromosome 11	plus	ampd2b<<br>XM_041799459.1	~~~~~~~~~~~~~~	gpr61<<br>XM_041800381.1	amigo1><br>XM_041798557.1	~~~~~~~~~~~~~~	kcnc4><br>XM_041799357.1		slc16a7<<br>XM_041800119.1	~~~~~~~~~~~~~~	rbm15><br>XM_041798819.1	slc16a4<<br>XM_041798821.1		lamtor5<<br>XM_041799562.1	prok1><br>M_041800363.1			znf335<<br>XM_041800065.1	mmp9<<br>XM_041800336.1		adora1b<<br>XM_041800035.1	dclre1b><br>XM_041798432.1							
Ballan wrasse (*Labrus bergylta*)	FKLU01000823.1	minus								slc6a17<<br>ENSLBET00000011343.1	~~~~~~~~~~~~~~	rbm15><br>ENSLBET00000011276.1	slc16a4<<br>ENSLBET00000011188.1		lamtor5<<br>ENSLBET00000011164.1	prok1><br>ENSLBET00000011131.1			znf335<<br>ENSLBET00000011024.1											
Gilthead seabream (*Sparus aurata*)	Primary assembly 7	minus	ampd2b<<br>XM_030424448.1	~~~~~~~~~~~~~~	gpr61<<br>XM_030421865.1	amigo1><br>XM_030423468.1	~~~~~~~~~~~~~~	kcnc4><br>ENSSAUT00010069888.1		slc6a17<<br>ENSSAUT00010069887.1	~~~~~~~~~~~~~~	rbm15><br>ENSSAUT00010069884.1	slc16a4<<br>ENSSAUT00010069883.1		lamtor5<<br>ENSSAUT00010069879.1	prok1><br>ENSSAUT00010069878.1	pga2><br>ENSSAUG00010026579		znf335<<br>ENSSAUT00010069875.1	mmp9<<br>ENSSAUT00010069781.1		adora1<<br>ENSSAUT00010069670.1	dclre1b><br>ENSSAUT00010069669.1							
Gilthead seabream (*Sparus aurata*)	Chromosome 6	plus								slc6a17<<br>XM_030420908.1							pga3><br>XM_030419593.1										amigo1<<br>XM_030419591.1	gpr61><br>XM_030418581.1		ampd2a><br>XM_030419506.1
Three-spined stickleback (*Gasterosteus aculeatus*)	Group XII	plus	ampd2b<<br>ENSGACT00000010229.1	~~~~~~~~~~~~~~	gpr61<<br>ENSGACT00000010367.1	amigo1><br>ENSGACT00000010372.1	~~~~~~~~~~~~~~	kcnc4><br>ENSGACT00000010500.1		slc6a17<<br>ENSGACT00000010522.1		rbm15><br>ENSGACT00000010526.1	slc16a4<<br>ENSGACT00000010535.1			prok1><br>ENSGACT00000010544.1	pga2><br>ENSGACT00000010638.1		znf335<<br>ENSGACT00000010651.1	mmp9<<br>ENSGACT00000010686.1		adora1<<br>ENSGACT00000010709.1	dclre1b><br>ENSGACT00000010714.1							
Three-spined stickleback (*Gasterosteus aculeatus*)	Scaffold 27	minus								slc6a17<<br>ENSGACT00000001331.1		rbm15><br>ENSGACT00000001329.1					pga3><br>ENSGACG00000001016										amigo1<<br>ENSGACT00000001315.1	gpr61><br>ENSGACT00000001313.1		ampd2a><br>ENSGACT00000001309.1
Ocean sunfish (*Mola mola*)	KV751364.1	plus	ampd2b<<br>ENSMMOG00000000383	~~~~~~~~~~~~~~	gpr61<<br>ENSMMOG00000000693	amigo1><br>ENSMMOG00000000698	~~~~~~~~~~~~~~	kcnc4><br>ENSMMOG00000001520>		slc6a17<<br>ENSMMOG00000002349	~~~~~~~~~~~~~~	rbm15><br>ENSMMOG00000002357	slc16a4<<br>ENSMMOG00000002360		lamtor5<<br>ENSMMOG00000002366	prok1><br>ENSMMOG00000002370	pga-ps><br>ENSMMOG00000002372<br>ENSMMOG00000002376		znf335<<br>ENSMMOG00000002386	mmp9<<br>ENSMMOG00000002393		adora1b<<br>ENSMMOG00000002398	dclre1b><br>ENSMMOG00000002401							
Japanese pufferfish (Takifugu rubripes)	Chromosome 3	plus	ampd2b<<br>XM_029833593.1	~~~~~~~~~~~~~~	gpr61<<br>XM_011621952.2	amigo1><br>XM_011621951.2	~~~~~~~~~~~~~~	kcnc4><br>ENSTRUT00000008903.3		slc6a17<<br>ENSTRUT00000007256.3	~~~~~~~~~~~~~~	rbm15><br>ENSTRUT00000006858.3	slc16a4<<br>ENSTRUT00000004089.3		lamtor5<<br>ENSTRUT00000057686.2	prok1><br>ENSTRUT00000003885.3			znf335<<br>ENSTRUT00000003060.3	mmp9<<br>ENSTRUT00000002003.3		adora1<<br>ENSTRUT00000041931.3	dclre1b><br>ENSTRUT00000049513.2							
Spotted green pufferfish (Tetraodon nigroviridis)	Chromosome 9	plus	ampd2b<<br>ENSTNIT00000018424.1	~~~~~~~~~~~~~~	gpr61<<br>ENSTNIT00000004126.1	amigo1><br>ENSTNIT00000018418.1	~~~~~~~~~~~~~~	kcnc4><br>ENSTNIT00000018411.1		slc6a17<<br>ENSTNIT00000018410.1	~~~~~~~~~~~~~~	rbm15><br>ENSTNIT00000018408.1	slc16a4<<br>ENSTNIT00000008929.1		lamtor5<<br>ENSTNIT00000018406.1	prok1><br>ENSTNIT00000018405.1			znf335<<br>ENSTNIT00000018404.1	mmp9<<br>ENSTNIT00000018403.1		adora1<<br>ENSTNIT00000018402.1	dclre1b><br>ENSTNIT00000018401.1							

Supplementary Table 11. Analysis of the synteny of *atp4a* in the genomes of monotremes and related species of tetrapods

Species	Chromosome, scaffold, etc	Direction	Annotated genes in each region								
Coelacanth (Latimeria chalumnae)	NW_005822144.1	plus				>ATP4A<br>ENSLACG00000002884	<TMEM147<br>ENSLACG00000004245	<GAPDHS<br>ENSLACG00000004825			
Tropical clawed frog (Xenopus tropicalis)	Primary_assembly 7	plus		<HAUS5<br>ENSXETG00000031041	....	>ATP4A<br>ENSXETG00000006825	<TMEM147<br>ENSXETG00000044204				
Anole lizard (Anolis carolinensis)	NW_003338828.1	minus				>ATP4A<br>ENSACAG00000010158	<TMEM147<br>ENSACAG00000037129	<GAPDHS<br>ENSACAG00000010085			
Platypus (*Ornithorhynchus anatinus*)	Primary_assembly 5	minus			>RBM42<br>ENSOANG00000048897		>TMEM147<br>ENSOANG00000037599	<GAPDHS<br>ENSOANG00000008109	>SBSN<br>XM_038767622.1	>DMKN<br>XM_029065299.2	>KRTDAP<br>XM_029065350.2
Echidna (Tachyglossus aculeatus)	Chromosome 26	minus		>HAUS5<br>XM_038766874.1	>RBM42<br>XM_038766884.1		>TMEM147<br>XM_038766898.1	<GAPDHS<br>XM_038766890.1	>SBSN<br>XM_038767622.1	>DMKN<br>XM_038766894.1	
Human (Homo sapiens)	Chromosome 19	minus	RBM42<br><ENSG00000126254	HAUS5<br><ENSG00000249115	....	>ATP4A<br>ENSG00000105675	>TMEM147<br>ENSG00000105677	<GAPDHS<br>ENSG00000105679	>SBSN<br>ENSG00000189001	>DMKN<br>ENSG00000161249	>KRTDAP<br>ENSG00000188508

Supplementary Table 12. Analysis of the synteny of *atp4b* in the genomes of monotremes and related species of tetrapods

Species	Chromosome, scaffold, etc	Direction	Annotated genes in each region									
Coelacanth (Latimeria chalumnae)	NW_005819439.1	minus	>GAS6<br>ENSLACG00000014211	<TMEM255B<br>ENSLACG00000013997	<GRK1<br>ENSLACG00000012291	>ATP4B<br>ENSLACG00000012034	<TFDP1<br>ENSLACG00000011116	<TMCO3<br>ENSLACG00000010362	>DCUN1D2<br>ENSLACG00000009664		>GRTP1<br>ENSLACG00000007224	<LAMP1<br>ENSLACG00000005998
Tropical clawed frog (Xenopus tropicalis)	Primary_assembly 2	plus			<GRK1<br>XM_002936972.5	>ATP4B<br>NM_001005806.1	<TFDP1<br>XM_031895955.1	<TMCO3<br>ENSXETG00000017774	>DCUN1D2<br>XM_012956438.3	>ADPRHL1<br>XM_002944826.5	>GRTP1<br>XP_012812169.1	< LAMP1<br>XP_012812038.1
Anole lizard (Anolis carolinensis)	Primary_assembly 3	minus	>GAS6<br>ENSACAG00000000689	<TMEM255B<br>ENSACAG00000000417		>ATP4B<br>ENSACAG00000039908	<TFDP1<br>ENSACAG00000000428	<TMCO3<br>ENSACAG00000000367	>DCUN1D2<br>ENSACAG00000000309	....	>GRTP1<br>ENSACAG00000000265	< LAMP1<br>ENSACAG00000000264
Platypus (*Ornithorhynchus anatinus*)	Primary_assembly 20	plus			<GRK1<br>ENSOANG00000015626	>ATP4B-PS<br>XM_039915013.1	<TFDP1<br>ENSOANG00000015627	<TMCO3<br>ENSOANG00000015628	>DCUN1D2<br>ENSOANG00000043642	>ADPRHL1<br>ENSOANG00000015632<br>XP_039770897.1	>GRTP1<br>ENSOANG00000015634	<LAMP1<br>ENSOANG00000015635
Echidna (Tachyglossus aculeatus)	Chromosome 20	plus			<GRK1<br>XM_038762090.1	>ATP4B-PS<br>XM_038761646.1	<TFDP1<br>XM_038762040.1	<TMCO3<br>XM_038761582.1	]>DCUN1D2<br>XM_038761586.1	>TMCO3<br>XM_038761580.1	>GRTP1<br>XM_038761859.1	<LAMP1<br>ENSOANG00000015635<br>XM_038761899.1<br>XP_038617827.1
Human (Homo sapiens)	Chromosome 13	minus	>GAS6<br>ENSG00000183087	<TMEM255B<br>ENSG00000184497	<GRK1<br>ENSG00000185974	>ATP4B<br>ENSG00000186009	<TFDP1<br>ENSG00000198176	<TMCO3<br>ENSG00000150403	]>DCUN1D2<br>ENSG00000150401			

Supplementary Table 13. Analysis of the synteny of *pga* in the genomes of monotremes and related species of tetrapods

Species	Chromosome, scaffold, etc	Direction	Annotated genes in each region													
Coelacanth (Latimeria chalumnae)	NW_005819048.1	plus						<PGA-PS<br>LOC102362369	<PGA<br>XM_005989231.1	<PGA<br>XM_005989232.1	<PGA<br>XM_005989233.1	<PGA<br>XM_014484601.1				
Tropical clawed frog (Xenopus tropicalis)	chromosome: 7	plus		>TMEM132A<br>XM_031905620.1					<PGA<br>XM_002942112.4							
Anole lizard (Anolis carolinensis)	NW_003338782.1	plus	>TMEM109<br>XM_016995560.1	>TMEM132A<br>XM_008115841.2	<SLC15A3<br>XM_003224127.3	>CD6<br>XM_008115843.2	>CD5<br>XM_008115847.2	<VPS37C<br>XM_003224128.3	>PGA<br>XM_003224113.3			<VWCE<br>XM_008115852.2	<DDB1<br>XM_003224115.3	>TKFC<br>XM_008115855.2	<CYB561A3<br>XM_003224116.2	>TMEM138<br>XM_008115857.2
Platypus (*Ornithorhynchus anatinus*)	Chromosome 3	minus	>TMEM109<br>XM_029058954.1	>TMEM132A<br>XM_029060926.2	<SLC15A3<br>XM_029060927.2	>CD6<br>XM_029061047.2	>CD5<br>XM_029059497.1	<VPS37C<br>XM_029060930.2				<VWCE<br>XM_029061107.2	<DDB1<br>XM_029060812.2	>TKFC<br>XM_029060813.1	<CYB561A3<br>XM_029060829.2	>TMEM138<br>XM_029060833.1
Echidna (Tachyglossus aculeatus)	Chromosome 22	plus	>TMEM109<br>XM_038764482.1	>TMEM132A<br>XM_038764750.1	<SLC15A3<br>XM_038764751.1	>CD6<br>XM_038765338.1	>CD5<br>XM_038765340.1	<VPS37C<br>XM_038764345.1				<VWCE<br>XM_038764389.1	<DDB1<br>XM_038764359.1	>TKFC<br>XM_038764395.1	<CYB561A3<br>XM_038764398.1	>TMEM138<br>XM_038764228.1
Human (Homo sapiens)	Chromosome 11	plus	>TMEM109<br>ENSG00000110108	>TMEM132A<br>ENSG00000006118	<SLC15A3<br>ENSG00000110446	>CD6<br>ENSG00000013725	>CD5<br>ENSG00000110448	<VPS37C<br>ENSG00000167987	>PGA3<br>ENSG00000229859	>PGA4<br>ENSG00000229183	>PGA5<br>ENSG00000256713	<VWCE<br>ENSG00000167992	<DDB1<br>ENSG00000167986	>TKFC<br>ENSG00000149476	<CYB561A3<br>ENSG00000162144	>TMEM138<br>ENSG00000149483

Supplementary Table 14. Analysis of the synteny of *pgc* in the genomes of monotremes and related species of tetrapods

Species	Chromosome, scaffold, etc	Direction	Annotated genes in each region															
Coelacanth (Latimeria chalumnae)	NW_005819795.1	minus									>FRS3<br>XM_014492193.1	<PGC<br>ENSLACG00000013732<br>XM_014492192.1				>TFEB<br>XM_014492188.1	<MDFI<br>XM_006002281.2	<FOXP4<br>XM_014492191.1
Tropical clawed frog (Xenopus tropicalis)	Primary_assembly 2	plus	>TOMM6<br>ENSXETG00000037184	<USP49<br>ENSXETG00000011843	………	<BYSL<br>ENSXETG00000011846	>MED20<br>ENSXETG00000011847		………	<PRICKLE4<br>ENSXETG00000033725	>FRS3<br>ENSXETG00000011850	>PGC<br>ENSXETG00000025732<br>XM_002932979.3	>PGC<br>XM_002932982.3	>PGC<br>NM_001030432.1	>PGC<br>NM_001015682.1	>TFEB<br>ENSXETG00000011854	<MDFI<br>ENSXETG00000048687	<FOXP4<br>ENSXETG00000011856
Anole lizard (Anolis carolinensis)	Primary_assembly 4	plus			>CCND3<br>XM_003220325.3	<BYSL<br>ENSACAG00000003079<br>XM_003220326.2	>MED20<br>XM_003220327.3	>USP49<br>ENSACAG00000002993<br>XM_016993283.1	<TOMM6<br>ENSACAG00000040820<br>XM_003220379.3	<PRICKLE4<br>XM_008109687.2	>FRS3<br>ENSACAG00000002874	>PGC<br>ENSACAG00000024788<br>XM_003220329.3	>PGC-PS<br>XM_016993287.1	>PGC-PS<br>XM_016993288.1	>PGC<br>ENSACAG00000002730<br>XM_003220331.3	>TFEB<br>ENSACAG00000002664<br>XM_008109690.2	<MDFI<br>ENSACAG00000002662<br>XM_008109691.2	<FOXP4<br>ENSACAG00000002366
Platypus (*Ornithorhynchus anatinus*)	Primary_assembly 7	minus			>CCND3<br>ENSOANG00000039929	<BYSL<br>ENSOANG00000038456	>MED20<br>ENSOANG00000038647	>USP49<br>ENSOANG00000046566	<TOMM6<br>XM_029069660.1		>FRS3<br>XM_029068355.1					>TFEB<br>ENSOANG00000015438	<MDFI<br>XM_029069363.1	<FOXP4<br>ENSOANG00000037557
Echidna (Tachyglossus aculeatus)	Chromosome 7	plus			>CCND3<br>XM_038749561.1	<BYSL<br>XM_038749560.1	>MED20<br>ENSG00000266494<br>XM_038749223.1	>USP49<br>XM_038749777.1	<TOMM6<br>ENSG00000112561<br>XM_038749786.1		>FRS3<br>XM_038749782.1					>TFEB<br>XM_038748956.1	<MDFI<br>XM_038749339.1	<FOXP4<br>XM_038749078.1
Human (Homo sapiens)	Chromosome 6	minus			>CCND3<br>ENSG00000112576	<BYSL<br>ENSG00000112578	>MED20<br>ENSG00000124641	>USP49<br>ENSG00000164663	<TOMM6<br>ENSG00000214736	<PRICKLE4<br>ENSG00000278224	>FRS3<br>ENSG00000137218	>PGC<br>ENSG00000096088				>TFEB<br>ENSG00000112561	<MDFI<br>ENSG00000112559	<FOXP4<br>ENSG00000137166

Supplementary Table 15. Analysis of the synteny of *kcne2* in the genomes of monotremes and related species of tetrapods

Species	Chromosome, scaffold, etc	Direction	Annotated genes in each region									
Coelacanth (Latimeria chalumnae)	NW_005819150.1	plus	>ITSN1<br>XM_014486558.1	<ATP5O<br>XM_005992832.2	>SLC5A3<br>XM_014486570.1		>KCNE2<br>XM_005992834.1	>SMIM11A<br>XM_005992837.2	<C21ORF140<br>XM_005992838.2			>KCNE1<br>XM_005992846.1
Tropical clawed frog (Xenopus tropicalis)	Chromosome 4	plus					>KCNE2<br>XM_018093231.2					
Anole lizard (Anolis carolinensis)	Chromosome 3	minus	>ITSN1<br>XM_008107551.2	<ATP5O<br>XM_003219003.3	>SLC5A3<br>XM_008107547.2	>MRPS6<br>XM_008107548.2	>KCNE2<br>XM_008107544.2	>SMIM11A<br>XM_008107543.2	< C3H21ORF140<br>XM_008107542.2			<KCNE1<br>XM_016992477.1
Platypus (*Ornithorhynchus anatinus*)	Chromosome 17	plus	>ITSN1<br>XM_029082331.2	<ATP5PO<br>XM_001513704.5	>SLC5A3<br>XM_029081967.2	>MRPS6<br>XM_029081971.1	>KCNE2<br>XM_029081778.2	>SMIM11A<br>XM_007669740.3		<FAM243A<br>XM_007669739.4	<SMIM34A<br>XM_039914407.1	<KCNE1<br>XM_001513365.3
Echidna (Tachyglossus aculeatus)	Chromosome 24	minus	>ITSN1<br>XM_038766277.1	<ATP5PO<br>XM_038766236.1	>SLC5A3<br>XM_038766097.1	>MRPS6<br>XM_038766099.1		> SMIM11A<br>XM_038766363.1		<FAM243A<br>XM_038766359.1		<KCNE1<br>XM_038766360.1
Human (Homo sapiens)	Chromosome 21	plus	> ITSN1<br>XM_017028428.2	<ATP5PO<br>NM_001697.3	> SLC5A3<br>NM_006933.7	>MRPS6<br>NM_032476.4	>KCNE2<br>NM_172201.2	>SMIM11<br>NM_001376899.1	< C21ORF140<br>NM_001282537.2		<SMIM34<br>NM_001367348.2	< KCNE1<br>XM_047440764.1

Supplementary Table 16. Analysis of the synteny of *vsig1* in the genomes of monotremes and related species of tetrapods

Species	Chromosome, scaffold, etc	Direction	Annotated genes in each region											
Coelacanth (Latimeria chalumnae)	NW_005819444.1	minus							>VSIG1<br>ENSLACG00000012909	<PSMD10<br>ENSLACG00000012845	>ATG4A<br>ENSLACG00000012655	<COL4A6<br>ENSLACG00000010637	>COL4A5<br>ENSLACG00000007854	<IRS4<br>ENSLACG00000006985
Tropical clawed frog (Xenopus tropicalis)	Chromosome 8	minus							>VSIG1<br>ENSXETG00000012851	<PSMD10<br>ENSXETG00000012853	>ATG4A<br>ENSXETG00000012854			
Anole lizard (Anolis carolinensis)	NW_003338749.1	plus	>SYTL2<br>ENSACAG0000001162<br>XP_016850543.1	>RAB39B<br>ENSACAG00000035673<br>XP_008112901.1	<VBP1<br>ENSACAG00000011753<br>XP_003223445.1		<PICALM<br>ENSACAG00000011799<br>XP_008112880.1	<GAB3<br>ENSACAG00000011922<br>XP_008112902.1	>VSIG1<br>ENSACAG00000034933	<PSMD10<br>ENSACAG00000043756<br>XP_003223415.1	>ATG4A<br>ENSACAG00000012035	<COL4A6<br>ENSACAG00000044407<br>XP_008112887.1	>COL4A5<br>ENSACAG00000012321	<IRS4<br>ENSACAG00000012623
Platypus (*Ornithorhynchus anatinus*)	Primary_assembly 6	plus	>GAB3<br>XM_029066959.2	>PICALM<br>XM_029067122.2	>VBP1<br>XM_029068015.2	<RAB39B<br>XM_029068020.1	<SYTL2<br>XM_039912425.1			<PSMD10<br>ENSOANG00000045915	>ATG4A<br>ENSOANG00000009658	<COL4A6<br>ENSOANG00000001272	>COL4A5<br>ENSOANG00000001275	<IRS4<br>ENSOANG00000038508<br>XP_039768307.1
Echidna (Tachyglossus aculeatus)	Chromosome 6	minus	>GAB3<br>XM_038747702.1	>PICALM<br>XM_038748598.1	>VBP1<br>XM_038747843.1	<RAB39B<br>XM_038747842.1	<SYTL2<br>XM_038748445.1		>VSIG1<br>XM_038747701.1	<PSMD10<br>XM_038748337.1	>ATG4A<br>XM_038748518.1	<COL4A6<br>XM_038748142.1	>COL4A5<br>XM_038747869.1	<IRS4<br>XM_038747682.1
Human (Homo sapiens)	Chromosome X	plus							>VSIG1<br>ENSG00000101842	<PSMD10<br>ENSG00000101843	>ATG4A<br>ENSG00000101844	<COL4A6<br>ENSG00000197565	>COL4A5<br>ENSG00000188153	<IRS4<br>ENSG00000133124<br>XP_039768307.1

Supplementary Table 17. Primers used in this study

Species	Gene	Accession	Remarks	Direction	Sequence (5' to 3')
Three-spined stickleback (*Gasterosteus aculeatus*)	*slc26a9*	XM_040192488	RT-PCR	Fw	tctctactcctctttctttcccctca
				Rv	atgtccatgtgatagagctcaggaag
			ISH	Fw	caacggtctctactcctctttc
				Rv	tacgaccagcatcacacatag
	kcne2	XM_040202413	RT-PCR	Fw	gcatctgatttgtccaacgtgaccctt
				Rv	gtatctgagccgtccagtcctcctt
			ISH	Fw	tccagtgctttaggacgttac
				Rv	caacatcgccttgttgtatctg
	vsig1	XM_040182500	RT-PCR	Fw	agattcacaactttcctgatgtggtc
				Rv	cgaactcaaactgggatatgtttctg
			ISH	Fw	ccatccagtgggactttgtt
				Rv	gagcacctcttcatctcattgg
	cldn18a	XM_040183298	RT-PCR	Fw	gacaacatcaaagccacaatgactct
				Rv	aatcaaatttctggttggacatcctt
			ISH	Fw	tcaggtgatgggcttcattc
				Rv	gggtcggtcctgtagatagt
	atp4a	XM_040164246	RT-PCR	Fw	gggagagagagtactgggtttctgtc
				Rv	caaaagactgaatggctcctatctga
			ISH	Fw	gctatcgtggtggcttatgt
				Rv	cgcaatgtcttccactgtttc
	atp4b	XM_040163626	RT-PCR	Fw	ggaaagtacttcatccaggacacctt
				Rv	ttttcgtagctgatgattttggagac
			ISH	Fw	actccgacgaagggaagtta
				Rv	tgtgccagtcttccgtagta
	pgc	XM_040162074	RT-PCR	Fw	atggacaacatgatcagtcaaaacct
				Rv	ctctctaaggaacacatctcccatga
			ISH	Fw	ctgagggacaaagggattgag
				Rv	ggtctgagtgatgtaagcagag
	pga2	XM_040194364	RT-PCR	Fw	gtttgggatcagcgagacagaagctc
				Rv	aggccaacggtctgattctccgtatc
			ISH	Fw	cgatgctgacttgtcctactac
				Rv	gacgtagaactgcctgatgaa
	actb	XM_040169876	RT-PCR	Fw	gggacgacatggagaagatctggca
				Rv	tgctgatccacatctgctggaaggt
humphead wrasse (*Cheilinus undulatus*)	*slc26a9*	XM_041800393	RT-PCR	Fw	aggcgtacagcaaggtgaagtccttac
				Rv	gaggaagagattactgctggcaatgttcc
	actb	XM_041786882.1	RT-PCR	Fw	tcgtaccacaggtatcgtgcttgactc
				Rv	aggctggtgatctctttctgcatacgg

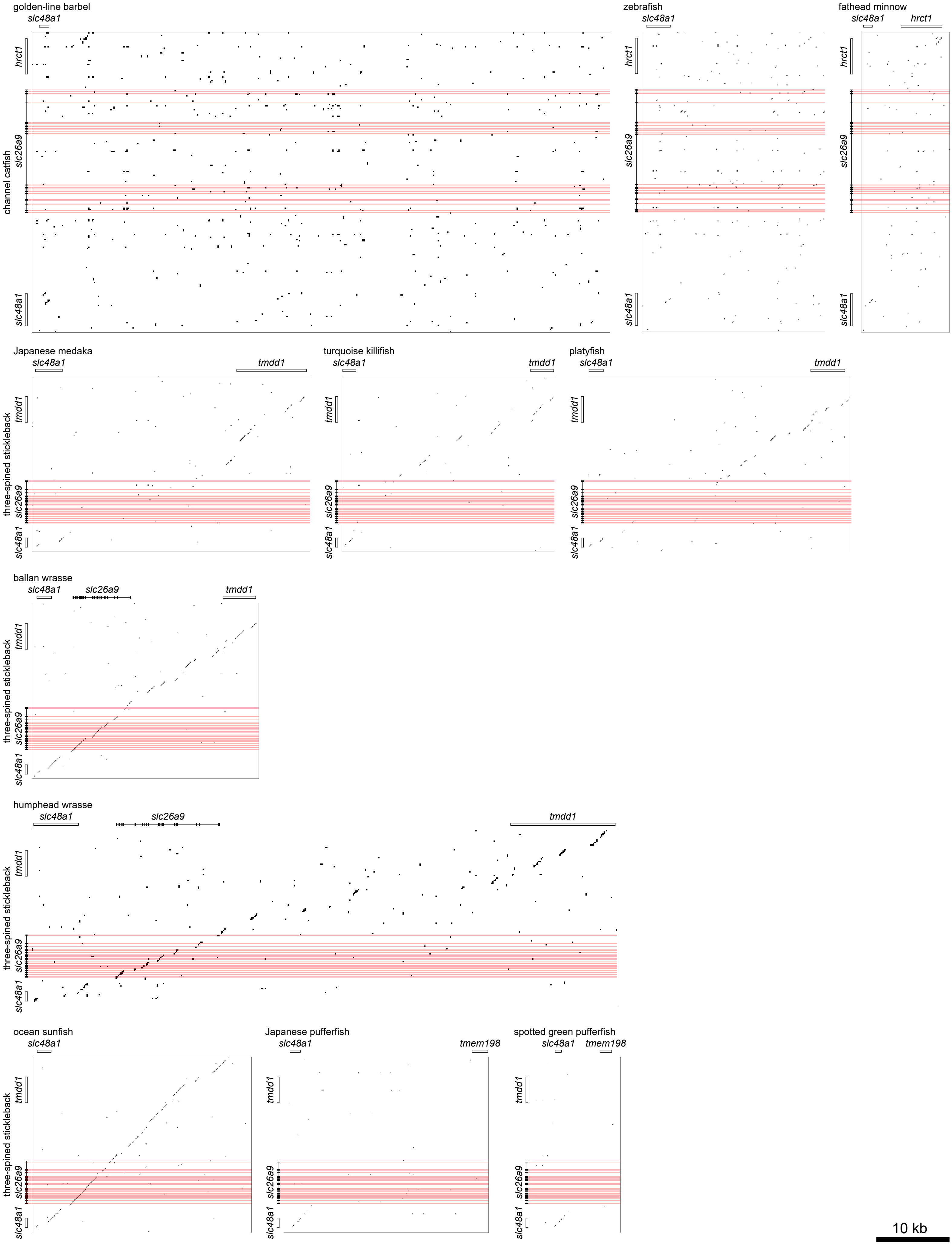

Supplementary Fig. 1. Dot plot analyses of *slc26a9*

The *slc26a9* gene and its flanking regions of three-spined stickleback (*Gasterosteus aculeatus*) or channel catfish (*Ictalurus punctatus*) in comparison with the corresponding genome regions of various agastric fish species are shown. Homologous regions were plotted with dotmatcher program (window size: 20; threshold: 70).

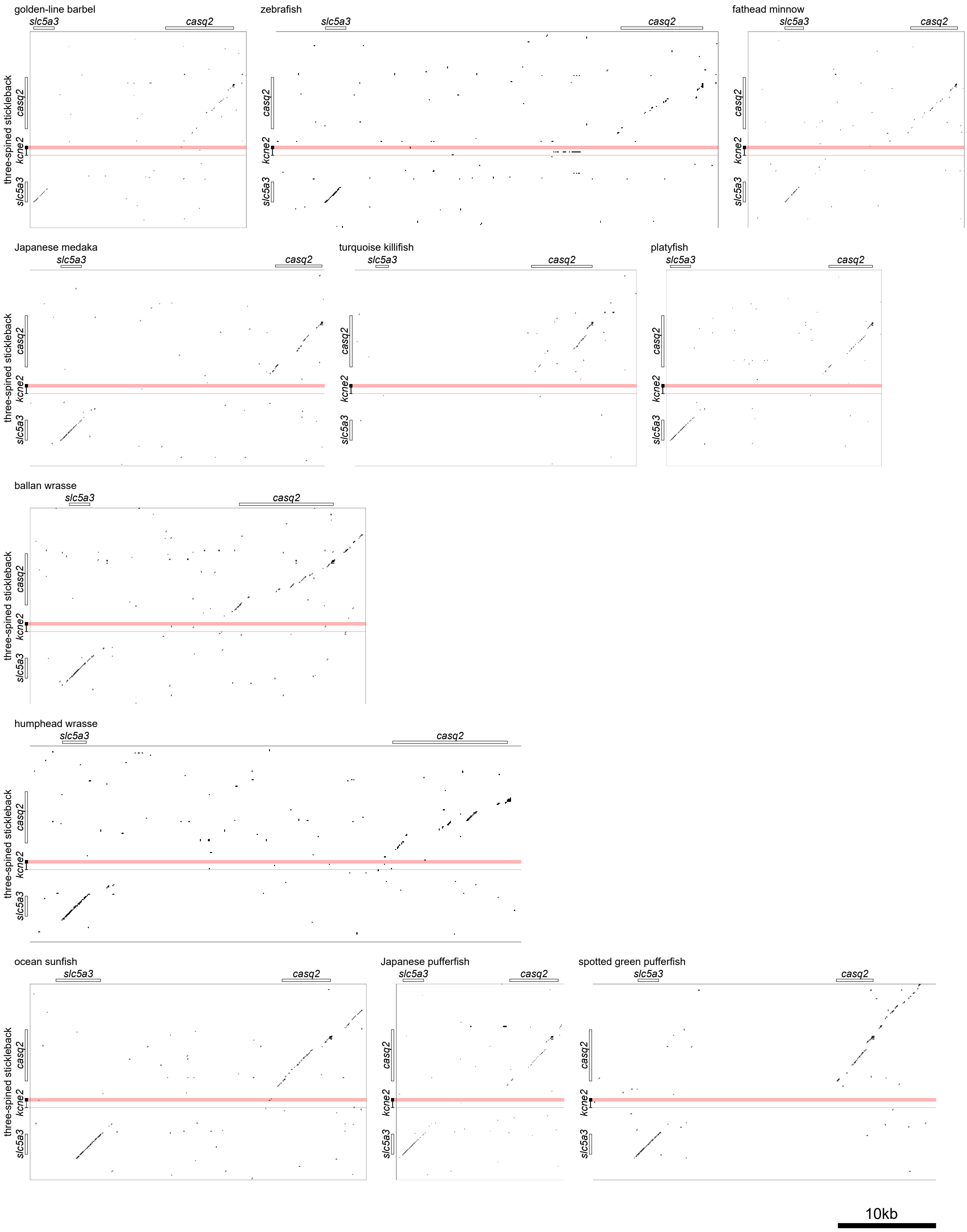

Supplementary Fig. 2. Dot plot analyses of *kcne2*

The *kcne2* gene and its flanking regions of three-spined stickleback (*Gasterosteus aculeatus*) or channel catfish (*Ictalurus punctatus*) in comparison with the corresponding genome regions of various agastric fish species are shown. Homologous regions were plotted with dotmatcher program (window size: 20; threshold: 70).

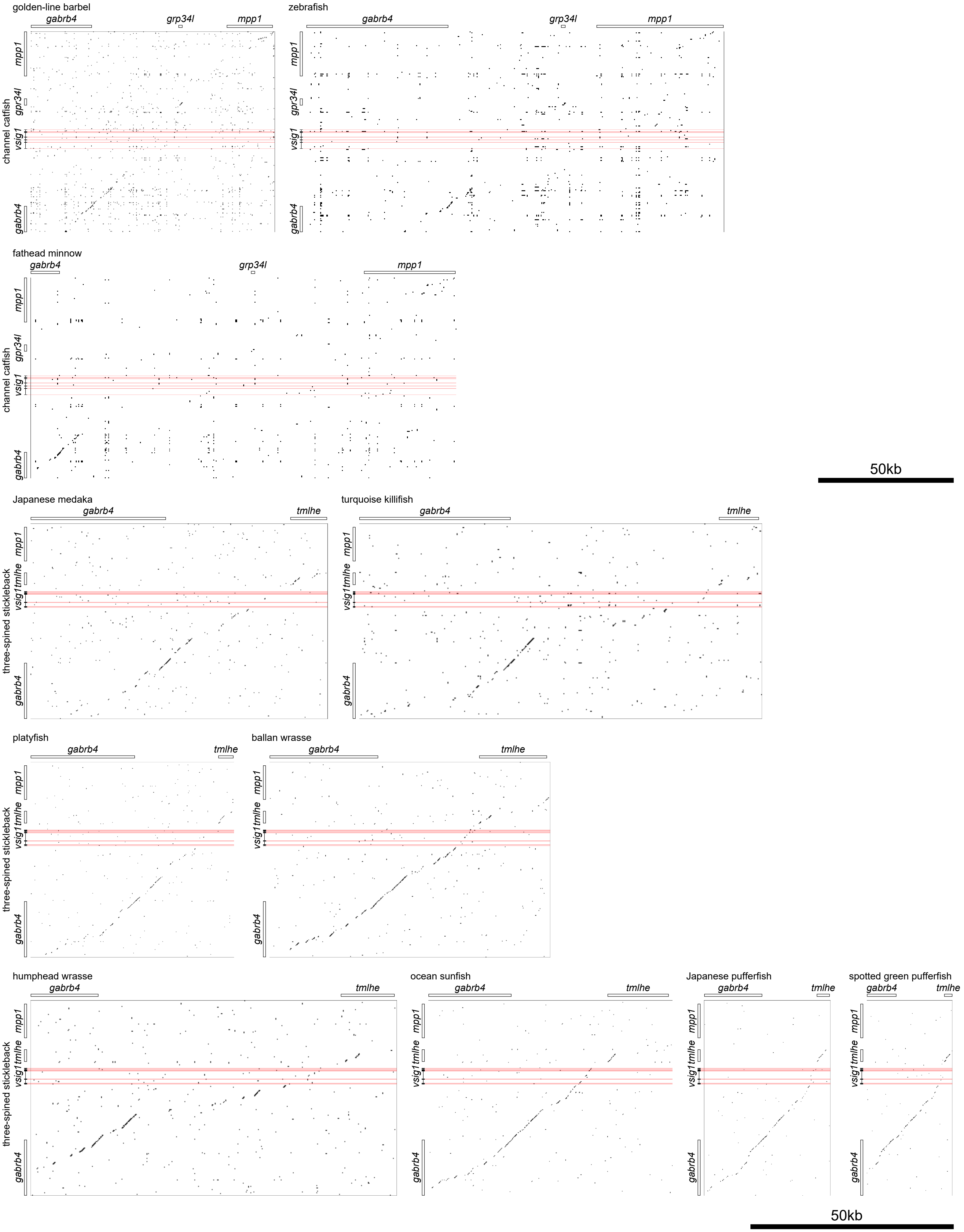

Supplementary Fig. 3. Dot plot analyses of *vsig1*

The *vsig1* gene and its flanking regions of three-spined stickleback (*Gasterosteus aculeatus*) or channel catfish (*Ictalurus punctatus*) in comparison with the corresponding genome regions of various agastric fish species are shown. Homologous regions were plotted with dotmatcher program (window size: 20; threshold: 70).

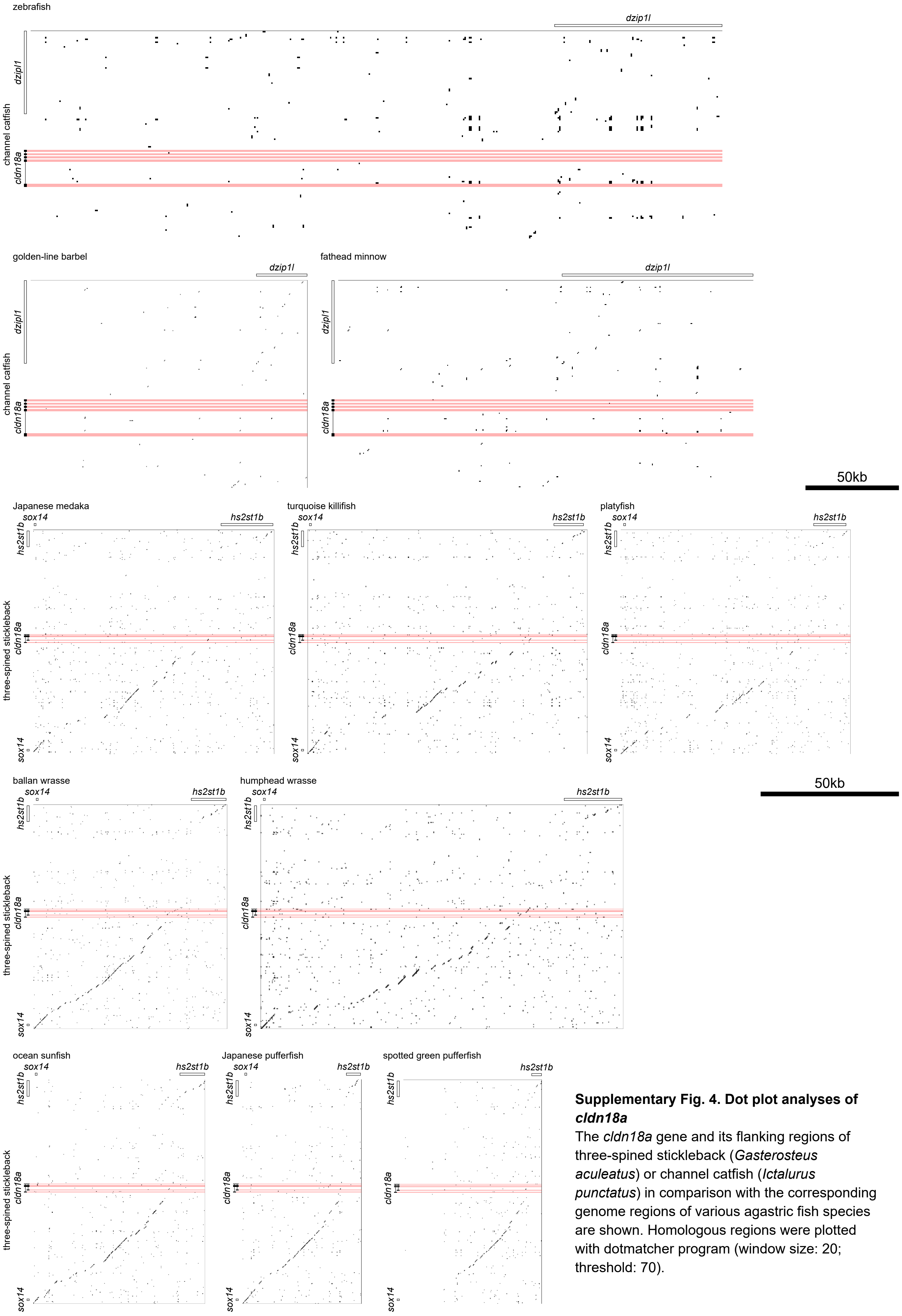

Supplementary Fig. 4. Dot plot analyses of *cldn18a*

The *cldn18a* gene and its flanking regions of three-spined stickleback (*Gasterosteus aculeatus*) or channel catfish (*Ictalurus punctatus*) in comparison with the corresponding genome regions of various agastric fish species are shown. Homologous regions were plotted with dotmatcher program (window size: 20; threshold: 70).

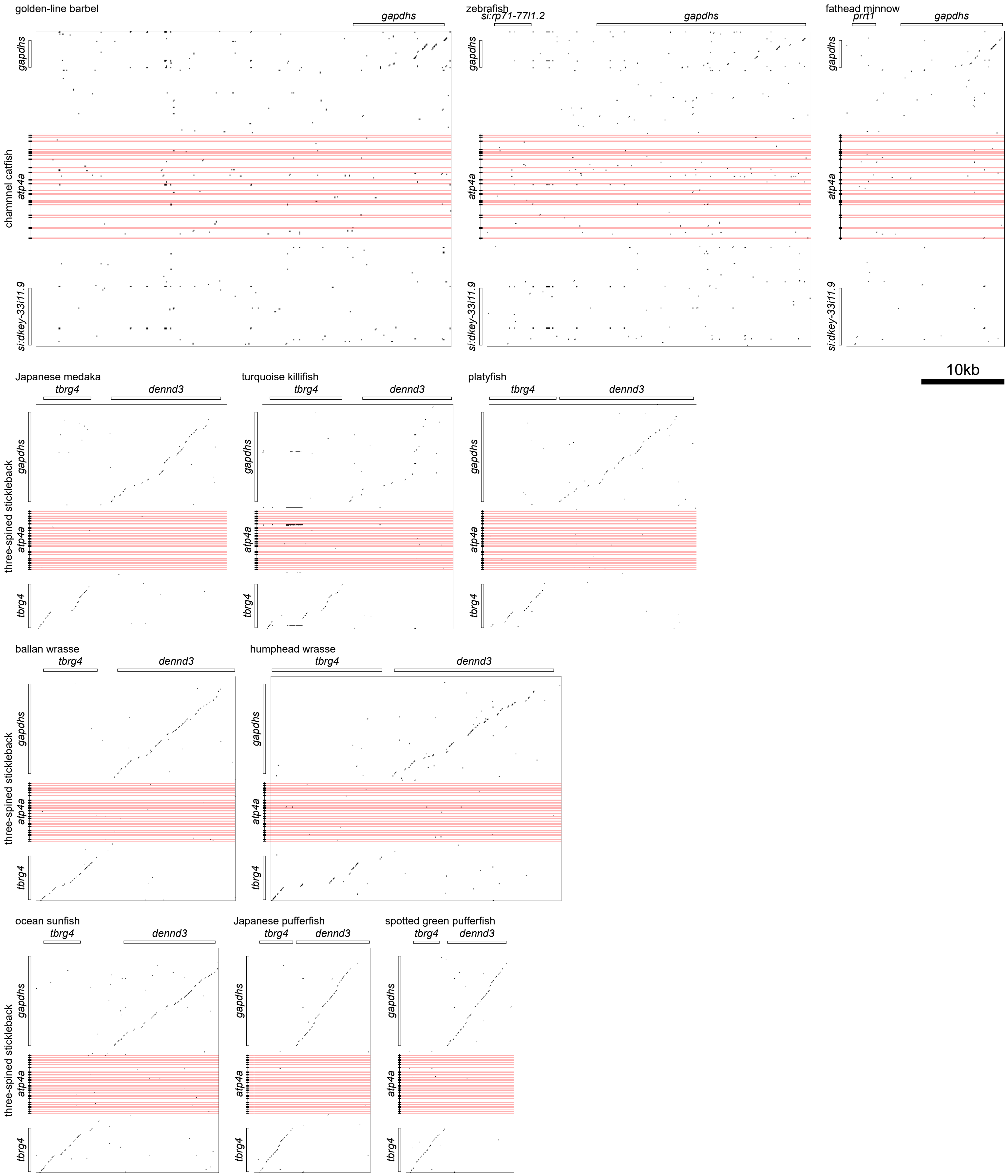

Supplementary Fig. 5. Dot plot analyses of *atp4a*

The *atp4a* gene and its flanking regions of three-spined stickleback (*Gasterosteus aculeatus*) or channel catfish (*Ictalurus punctatus*) in comparison with the corresponding genome regions of various agastric fish species are shown. Homologous regions were plotted with dotmatcher program (window size: 20; threshold: 70).

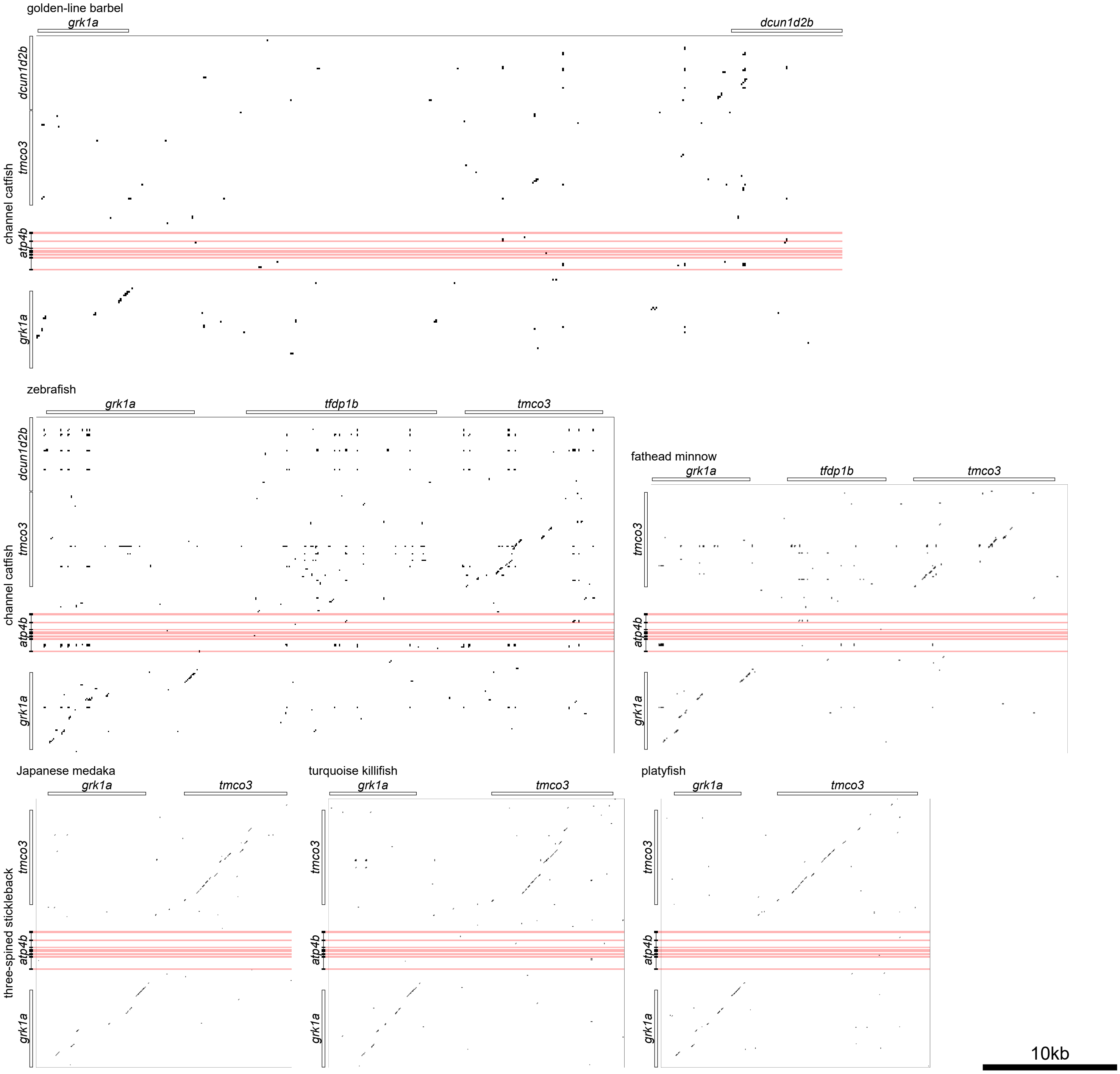

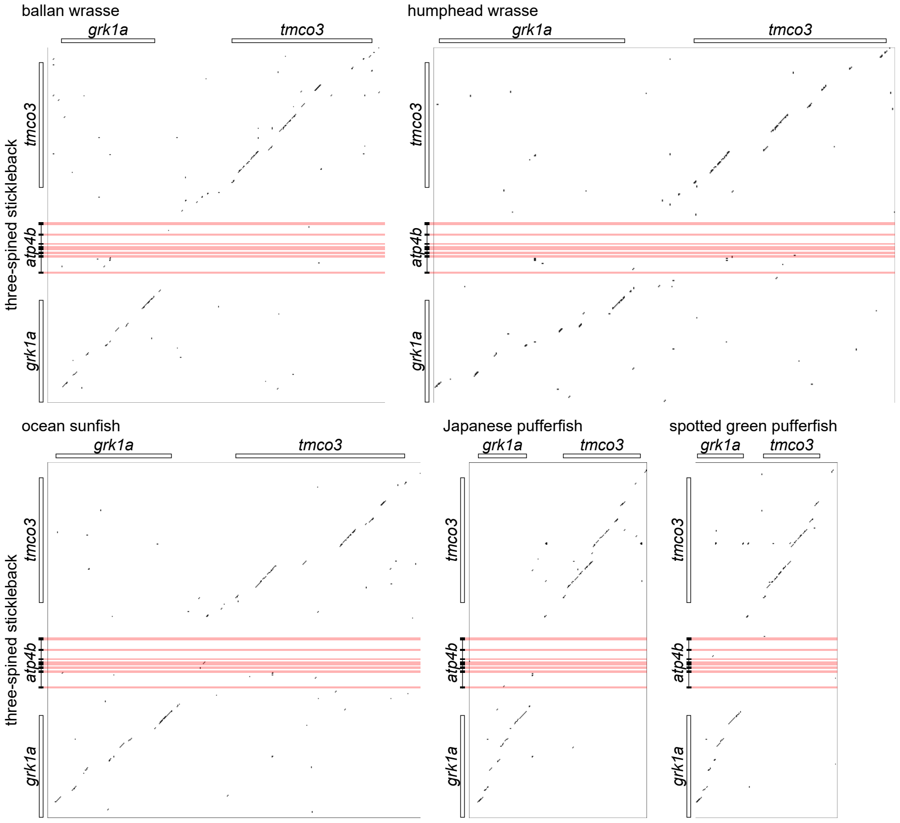

Supplementary Fig. 6. Dot plot analyses of *atp4b*

The *atp4b* gene and its flanking regions of three-spined stickleback (*Gasterosteus aculeatus*) or channel catfish (*Ictalurus punctatus*) in comparison with the corresponding genome regions of various agastric fish species are shown. Homologous regions were plotted with dotmatcher program (window size: 20; threshold: 70).

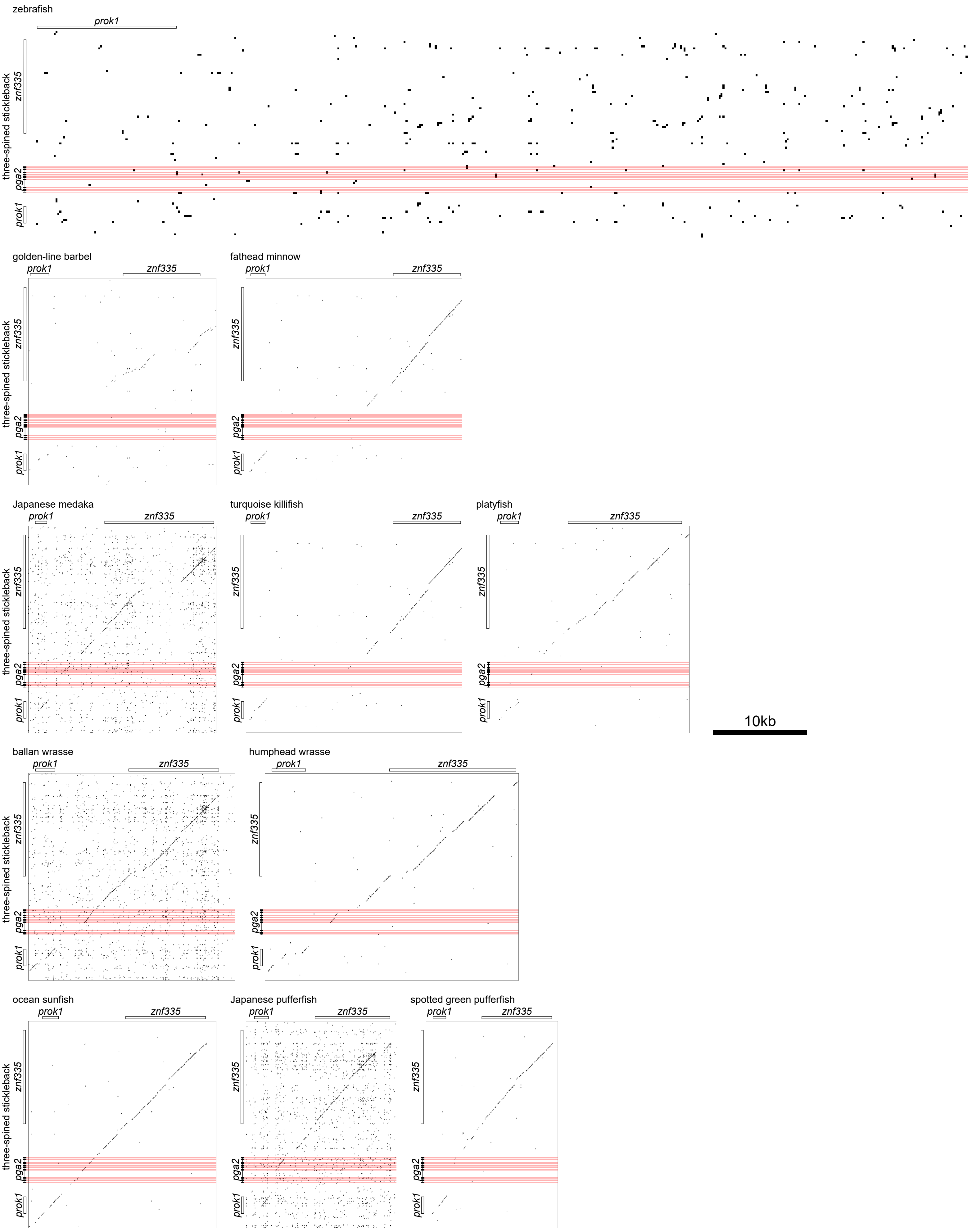

Supplementary Fig. 7. Dot plot analyses of *pga2*

The *pga2* gene and its flanking regions of three-spined stickleback (*Gasterosteus aculeatus*) or channel catfish (*Ictalurus punctatus*) in comparison with the corresponding genome regions of various agastric fish species are shown. Homologous regions were plotted with dotmatcher program (window size: 20; threshold: 70).

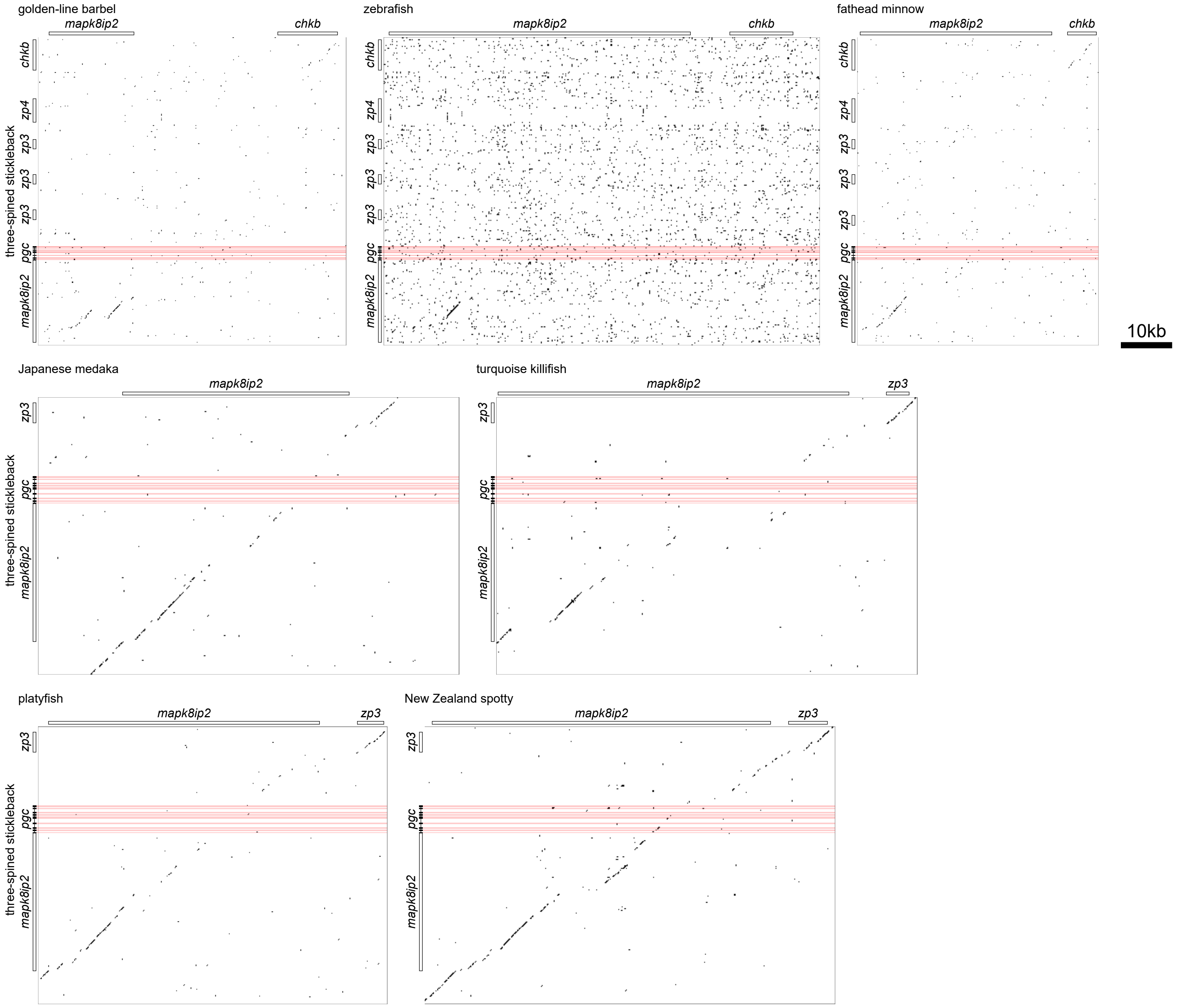

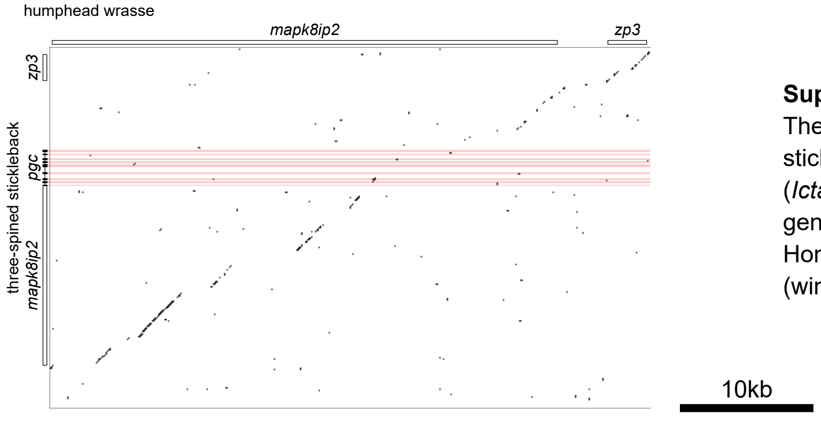

Supplementary Fig. 8. Dot plot analyses of pgc
The *pgc* gene and its flanking regions of three-spined stickleback (*Gasterosteus aculeatus*) or channel catfish (*Ictalurus punctatus*) in comparison with the corresponding genome regions of various agastric fish species are shown. Homologous regions were plotted with dotmatcher program (window size: 20; threshold: 70).

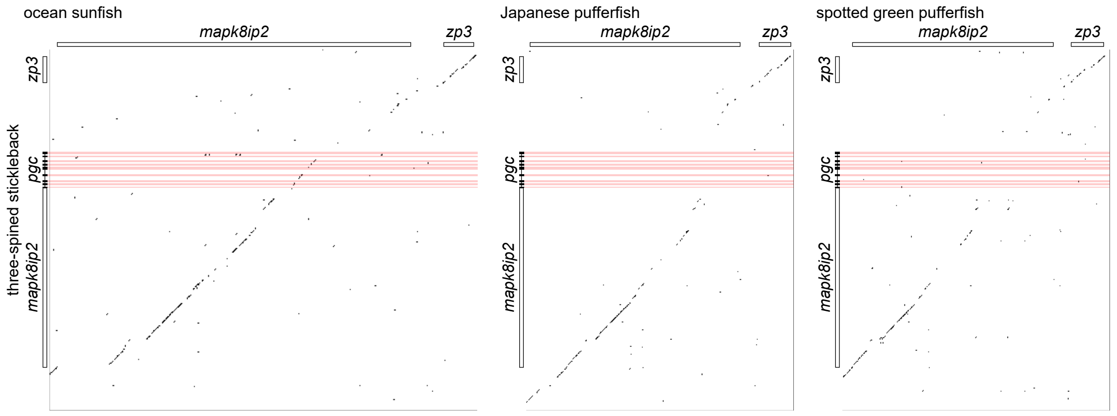

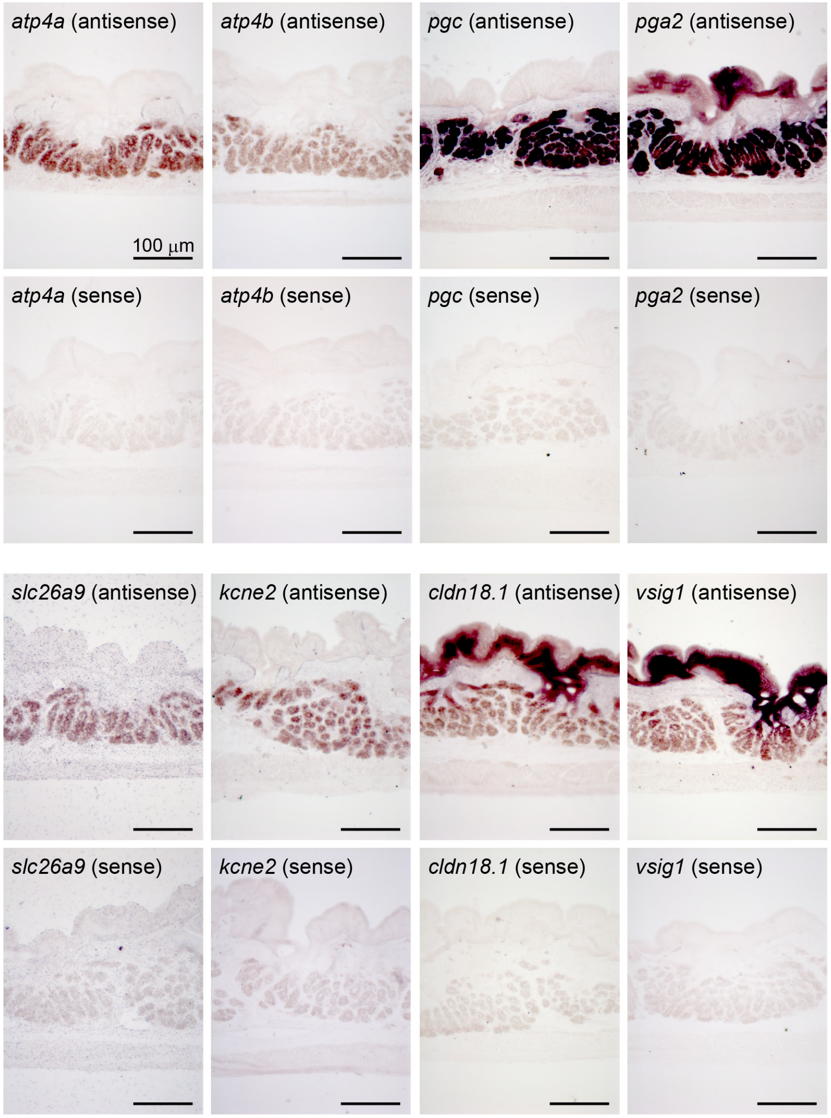

Supplementary Fig. 9. *In situ* hybridization histochemistry analysis of the three-spined stickleback stomach with antisense or sense probes. The gastric wall sections were stained with antisense or sense probes.

```
Xenopus tropicalis Atp4b    MATFNEKKTCSQRMENFGRFLWNPDTSEFMGRTFAKWVYISLYYAAFYVIMIGIFALSIY
coelacanth Atp4b            MATFNEKKTCSQRMENFQRFIWNPDAGELMGRTLINWVWISLYYVAFYCIMSCLFALALY
Nile tilapia Atp4b          MATLKEKRTCGQRCEDFGHFVWNSENGTFMGRTPEKWVYISLYYVAFYVIMTGLFSLAIW
Atlantic cod Atp4b          MAALKEKRTCGQRCEDFGRFVWNSDNGTLFGRTPEKWVYISLYYVAFYVVMTGLFCLAIW
anole lizard Atp4b          MATLNEKKTCSQRMENFQRFVWNPDTGQLMGRTLINWVWISLYYVAFYVVVTGLFALSIF
human Atp4b                 MAALQEKKTCGQRMEEFQRYCWNPDTGQMLGRTLSRWVWISLYYVAFYVVMTGLFALCLY
echidna Atp4b               ------------------------------------------------------------
platypus Atp4b              ------------------------------------------------------------

Xenopus tropicalis Atp4b    SLMKTMNPFVPDYQDELKSPGVTMRPDPYGDEVIELFYNKAENSTYLPLVTSLCDFLSVY
coelacanth Atp4b            VMLSTLNPYTPTYQDRLHAPGVMVRPDVFGEEGVQIFFNLSEKSSWERHVKTLQDFLSVY
Nile tilapia Atp4b          VLMYTISPYTPDYQDRLSSPGVMVWPDTYGEEDVEISYNTSDKASCMAMANILHDFLKPY
Atlantic cod Atp4b          TLMYTLDPYTPDYQDRLPSPGVMVWPDKYDEEHIVITYNSSDKVSVSKMSSCLHNFLGQY
anole lizard Atp4b          SLMMTLNPYTPDYQDRLKSPGVTLRPDVYGEKGLEIYYNVSEEHSWEGFVKTLQKFLSGY
human Atp4b                 VLMQTVDPYTPDYQDQLRSPGVTLRPDVYGEKGLEIVYNVSDNRTWADLTQTLHAFLAGY
echidna Atp4b               ----MRRCSPREQTSEGGANTPETSPLTFSREPLLEAR-EFSLSFRRSIFGQKAPVLAPY
platypus Atp4b              -------------MSAQDQPGVTLRPDVFRDEGLQLCN-ISDRSTWEVLTKIPHTFLSGY
                                          .          *  :  : :       .             .*  *

Xenopus tropicalis Atp4b    NKTVQEKMN-ANCSDN---TRMSCANPKENSKSCQFTTDMLGNCSWEHDHTFGYKSGTPC
coelacanth Atp4b            NKSVQFEKN-CNCTRGKYYFQDSFKGPDHTKYACRFIREMLGNCSGLQDPTFGYKVGQPC
Nile tilapia Atp4b          NDTKQLECNNYNCTKGKYFIQKTFSAPHHTKWVCPFTQSMLGPCSGIEDPTFGYNSTMPC
Atlantic cod Atp4b          NDTLQKDCD-HNCTKGKYFMQHNFLPGTHTKESCSFSVSQLGNCSGLHDPTFGYNCDMPC
anole lizard Atp4b          NETAQQANK--NCYREGYYFQKKFDAPNHTKHSCKFTQEMLGNCSGLVDPNFGFSEGSPC
human Atp4b                 SPAAQEDSI--NCTSEQYFFQESFRAPNHTKFSCKFTADMLQNCSGLADPNFGFEEGKPC
echidna Atp4b               SSVAQHENQ--NCTSGEYDFQNKFLAPNHTKFSCKFTADRLQNCSGVQDRNFGFEDGKPC
platypus Atp4b              SPIAQNENQ--NCTSGEYHFQKKFLAPNHIKFFC-FTADMLQNGSGVQDRNFGFEYGKPC
                            .   *      **       : .     . .  * *  . *   *   * .**:.   **

Xenopus tropicalis Atp4b    LFIKMNRIINFVPGNKTVPLVNCSAEHG--DLGDVHYYPGNDTYGTIGFQYFPYCGKKMQ
coelacanth Atp4b            FIIRMNRIINFLPGNGTAPYVNCTKVGGH-SLGETETFPVN---GTFDLSYFPYYGKKAQ
Nile tilapia Atp4b          VIIKMNRIIDFLPSNNTALPPYVNCTILD-GEDSVAKIEYFPDGGIMDPSYFPYYGKLAQ
Atlantic cod Atp4b          VIIKMNRIINYLPSKNNTQDIQVNCTVLE-GEENVKKIEYFPENALMDRSYFPYYGKLAQ
anole lizard Atp4b          LIIKMNRIINFLPGNGTEPRVNCTTLDDE-SPLDVQYYPRN---GTFKLHYFPYYGCKAQ
human Atp4b                 FIIKMNRIVKFLPSNGSAPRVDCAFLDQPRELGQPLQVKYYPPNGTFSLHYFPYYGKKAQ
echidna Atp4b               FIIKMNRIINFLPGTASPPRVECTTLDRK--ESEPLEVVYYPDNGTFSPHYFPYYGKKAQ
platypus Atp4b              VIIKMNRMINFLPSTASPPRVECTTP----------------------------------
                            .:*:***::.::*.. .

Xenopus tropicalis Atp4b    PNYTNPLVAVKLLNPPLNKELSVVCKVSGHGITSDNPHDPYEGKVSFKLKIEDKPGSSSA
coelacanth Atp4b            PNYTNPLIAVKFLNLERNKEITVECKVIGKEIITDNPHDPYEGKVLFRVKICD-------
Nile tilapia Atp4b          PTYVNPLVAVRFS-LVGEKDAKIQCRVVSEKISYENIHDPYEGKVVFYLKAVK-------
Atlantic cod Atp4b          PNYLNPLVAVRIS-LTGKRSAKFQCRVVADKITYENIHDPYEGKVVFTLQNLHVPSSPSS
anole lizard Atp4b          PSYSNPLVAVKLLNVPINKGIHVVCRVVGTGITSDNPHDPYEGKVEFKVCIKA-------
human Atp4b                 PHYSNPLVAAKLLNIPRNAEVAIVCKVMAEHVTFNNPHDPYEGKVEFKLKIEK-------
echidna Atp4b               PHYSNPLVAAKFLNIPEDVEVSVVCRVVADKITSNNPQDPYEGKVEFKVKIETADPVISC
platypus Atp4b              PHYSNPLVAAKFLNIPKDVKVDVVCRVAAVKITSDNPHDPCEGKVEFKSENSNP------
                            * * ***:*.::     .    . *:* .  :  :* :** **** *

Xenopus tropicalis Atp4b    N----------------------------------------
coelacanth Atp4b            -----------------------------------------
Nile tilapia Atp4b          -----------------------------------------
Atlantic cod Atp4b          VHHHSSTL---------------------------------
anole lizard Atp4b          -----------------------------------------
human Atp4b                 -----------------------------------------
echidna Atp4b               YSDPLSKHSTNKSTLSASLYTFLRRNHGVKYLPLSLSLERL
platypus Atp4b              -TGPHQISSGFSSTLNLEA----------------------
```

Supplementary Fig. 10. Multiple alignment of Atp4b sequences.
The predicted amino acid Atp4b sequences of echidna (GenBank accession number: XP_038617574) and platypus (XP_039770947) were aligned with amino acid sequences of Atp4b in human (NP_000696), anole lizard (XP_003218799) *Xenopus tropicalis* (NP_001005806), coelacanth (XP_005998136), Atlantic cod (XP_030233591), and Nile tilapia (XP_003445766). The proposed amino-terminal cytoplasmic and transmembrane domains are shown in green and magenta, respectively.

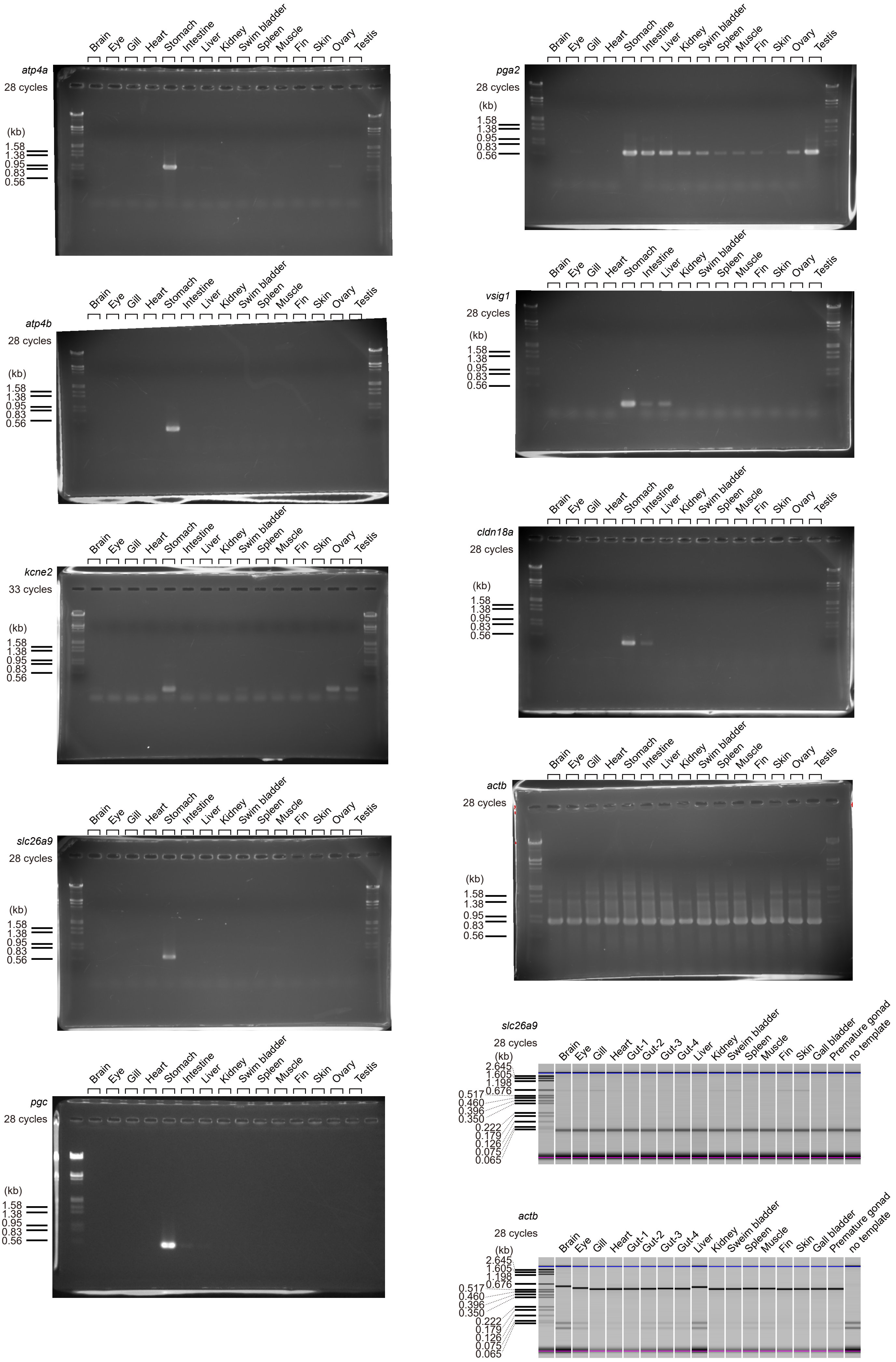

Supplementary Fig. 11. Whole images of agarose electrophoresis and a Microchip Electrophoresis system for DNA/RNA Analysis MCE-202 MultiNA.

cldn18b_stickleback
ATGGCGCCTTCAGTCCTGCAGAATGGTGGATTCGTTTCGGGTTTGGTCGGCGCTGCGGCCCTCATCGCAGCGACTGCCATGAACAATTGGAGCG
TCAAGGACCGGCAGGGGGATGTTGTGACATCCGTTTACACCTACAAGGGTCTGTGGCGGGACTGTGAGACCACTTCCTCCGGACTCACCGAGTG
CCGCCCGCTCTACGGCCTCCTGGGCTTCTCAGGTGTGTTCCAGGCCGTGCGAGCCCTGATGATCGTGGGCGTGGTGCTGGCCGTCCTGGCAGC
GGTGATATCAGTTTTCTCCCTGCTCATGAACAGCATGGCAGACACCACCAAGGCCAAGATGAGCCTGACCGCCGGCATCATGTGTGGCATCGCAG
GAGCTTCTATCTACGCCAATCAGATCGTAGCCAGTTTCAGGATGTCCAACCCCTACACATTTGGCCCTGCACTGTTCGTGGCCTGGATAGGAGGGG
GCGTCCTGCTCATTGGGGGTGTCCTCAAGTCACTGGCTTTCAGGGGGATG
cldn18b_cod
ATGGCGCCCTCGGTGCTGCAGACGGGCGGCTTCATACTGGGCGTGGTCGGCGCGGCCGCTCTCATCGCCGCCACCGCCATGAACAACTGGAGC
GTGAAGGACCGGCAGGGCGACGTGGTGACCTCGGTGTACACCTACAAGGGCCTGTGGCGGGACTGCGAGACCACCTCCTCCGGCCTCACCGA
GTGCCGGCCCCTCTACGGCCTGCTGGGCTTCTCAGGCATGTTCCAGGCAGTGCGGGCGCTCATGATCGTGGGTGTGGTCCTGAGCGTGCTCGG
GGCGCTGATCTCCGTTTTTTCCTTGCTCATGAACAGCATGGCCGACTCCTCCAAGGCCAAGATGAGCCTCACCGCCGGCGTCATGTGTGGCATTG
CGGGGGCGTCTATCTATGCCAACCAGATCGTGGCCAGCTTCAGGCAGGCCAGCCCATACACCTTCGGCCCGGGGCTGTTCGTGGCCTGGATCTC
CAGCGGGGTGCTGCTGGTGGCCGGGGTGCTGAAGAGCCTGGCCTTCAAGGGAATC
cldn18b_ tilapia
ATGGCGCCTTCAGTTCTGCAAAATGGTGGATTCATCTTGGGTCTCATCGGTGCAACTGCCCTCATTGCAGCCACAGCCATGAACAACTGGAGCGT
GAAGGACAGGCAGGAGCAGGTCGTGACCTCCGTTTATACCTACAAGGGTCTGTGGCAAGACTGTGAGACGACCTCCGCCGGACTCACAGAGTGC
CGCCCGCTCTACGGCCTTCTGGGTTACTCAGGTACCTTCCAGGCGGTGCGAGCTCTGATGGTAGTGGGTGTGGTGCTGAGCGTGCTCGGGGCG
CTGATATCAGTTTTTTCCCTGCTCATGAATAAGATGGCAGACAGCGCAAAGGCTAAGATGAGCCTCACTGCTGGCATCATGTGTGGCATTGCAGGA
GCTTCTGTGTATGCCAATCAAATTGTGGAAAGTTTCAGGACGTCCACCACCTACACATTTGGCCCAGCACTGTTCGTTGGCTGGATCGGAGGGGG
CGTTCTGCTCATTGGTGGCATCCTGAAGTCTCTCGCTTTCAGAGAGATG
cldn18b_Takifugu
ATGGCTCCTTCCGTCCTGCAGAACGGCGGATTCCTCCTGGCTGTGGTCGGCACCGCGGCCCTGATCGCTGCTACGGGCACCAGCAAATGGAGC
GTGAGGGACCGGCCGGGGGACGAGGAGGCGTCTCTGTACCGACACAAGGGTCTGTGGCAGGACTGCGCGACAACCTTCTCTGGGTTCACCCA
GTGCGGGTCGCTCCAGGGCGTTCTGAAGCCTCTCGGTGCCTTTTGGGCGGTGCGAGCCCTGATGATCATCGGCGTGCTGCTGAGCGTCCTCGG
AGCCATGATGTCGCTGTTCTCCCTGGTCCTGGGCAGCCTGGAGGACTCCACCAAGGCCCGGATGTGTCTGACAGCCGGGGTCGCCTGTGGCAT
CACAGGAGCTTCTATTTATGCCAGTCACATTCTGGCCAGTTTGAAGACGTCCACCCCATACACCTTTGGCCCTGCGTCCTTTGTGGGTCTGATGGG
AGGAAACGTTCTGGTCATGGCTGGGATCCTGAAGTCAATGGCCTTCAGAGAAATT
cldn18b_zebrafish
ATGAGTGCCCCAGCTCTGCAGACCACAGGCTTCGTTTCAGGCGTCATCGGGACGGCTGGTGTTTTTGCAGCCACCCTGATGGACGTCTGGTGTT
TCAGAAACCAGCAACAGGATAGGGTCAGCTCCATCTACACTTATAAGGGTCTGTGGAAGGACTGTGAGATGTCCGGGACTGTATTCCCTGAGTGC
CAGCCTCTCTACAGCCATCCCAACTACTCAGGAATACTCCAGGCTGCAAGAGCTCTGATGATAATAGCGATTTTAGTGGCTGTGATCGCAGTGTTCA
TCGGGTTTTTCTGCTTGCTCATGAAAAATATGCAGCTTTCCACCAGGGCCAAACTCATCCTAAGTTCTGCGATCATTTGTGGTATTGCTGCAGCATC
AGTGTATGCTGATCAACTAGTGCCTAGTTTCATGATGCCCCAGTCCTACACGTTTGGCCCTGCCCTCTACATAGCCTGGGTGGGAGCAGCTTTGTT
AATTCTGGGAGGAATTTTGATGAGCATCGCATACAAAAGAATG
cldn18_Xenopus
ATGTCGGTAACAATGTGTCAAACCATGGGCTTTCTTGTATCAGCCCTGGGCTTTGCTGGGATTATAGCAGCAACTGCTTTGGATCCATGGAGCACG
CAAGATTTATACGATAATCCAGTTACTGCTGTGTTCCAGTACCAGGGACTCTGGAAGAGTTGTGTCCAACAGAGTTCCGGATTCACCGAGTGTCGA
CCCTACTACACTATCCTTGGGTTACCAGCTATGTTCCAGGCAGTCAGAGCCCTGATGATTGTGGGCATAGTGTTGGGAGCCATTGGCTTACTTGTG
GCCATTTTTTCCCTGATTATTGGTAACATGGAGGATTCTGCCAAGGCCAACATTACCTTGACATCTGGTATCATGTGTTCAATAATCGGAGTATCTGTC
TTTGCCAACATGTTGATTACAAACTTCTGGATGAATATGACATATACTTTTGGGGCAGCCCTTTTTGTTGGATGGGTCGCTGGAGGCTTGACGTTGAT
TGGAGGTGTTATGATGTGCATAGCATGCCGTGGACTC
cldn18_coelacanth
ATGTCGGTGACCATGTGCCAATCGGTGGGGTTCCTGCTTTCTGTTCTGGGCCTGGCCGGGATCATCGCGGCCACCGGCCTGGACATGTGGAGTA
CCCAGGACCTTTACGGCAATCCAGTGACCGCTGTCTTCAACTACCAGGGACTGTGGCGGACGTGTGTGCGGGAGAGCTCTGGGTTCACGGAGT
GCCGACCCTACTTCACCATTCTGGGACTGCCAAGCATATTCCAGGCTGTGAGAGCCTTGATGATCGTTGGCCTTGTGTTGGGGGCCGTTTCTGCC
CTGGTTTCGATTTTTGCTATGATTATTGGAAACATGGATAACTCAGCTAAAGCCAAACTGACCTTGGTTTCAGGAATCATATGTGCTATTGTTGGTGTT
TCCATCTTTGCAAACATGCTGGTCACCAATTTCTGGATGTCATTCACTTACACGTTTGGTTCAGCCCTGTTTGTAGGCTGGGTTGGCGGTGGGCTG
GCAGTAGCAGGCGGAATCATGATGTGCATTGCCTGTAGAGGAATG
cldn18_human
ATGTCCACCACCACATGCCAAGTGGTGGCGTTCCTCCTGTCCATCCTGGGGCTGGCCGGCTGCATCGCGGCCACCGGGATGGACATGTGGAGC
ACCCAGGACCTGTACGACAACCCCGTCACCTCCGTGTTCCAGTACGAAGGGCTCTGGAGGAGCTGCGTGAGGCAGAGTTCAGGCTTCACCGAAT
GCAGGCCCTATTTCACCATCCTGGGACTTCCAGCCATGCTGCAGGCAGTGCGAGCCCTGATGATCGTAGGCATCGTCCTGGGTGCCATTGGCCTC
CTGGTATCCATCTTTGCCCTGATCATTGGCAGCATGGAGGACTCTGCCAAAGCCAACATGACACTGACCTCCGGGATCATGTGTGCAATTGCTGGA
GTGTCTGTGTTTGCCAACATGCTGGTGACTAACTTCTGGATGAACATGACCTACACATTTGGTGCGGCTCTGTTCGTGGGCTGGGTCGCTGGAGG
CCTCACACTAATTGGGGGTGTGATGATGTGCATCGCCTGCCGGGGCCTG
cldn18a_stickleback
ATGGCGGCCACCCTCTGTCAGGTGATGGGCTTCATTCTCAGTTTGCTAGGGGTGGCGGGAATAATAGCGGCGACGGGGATGGACCAGTGGGCGA
CGGAAGACCTCTTCGACAACCCCGTGACGGCCGTGTACTCGTACTCGGGCCTGTGGAACTCGTGCGTCCGGCAGAGCTCCGGATTCACCGAGT
GCCGGCCGTACTTCACCATTCTGGGACTGCCAGCTCTGCTGCAGGCCGTCCGGGCCTTGATGATCGTCGGCATCGTCCTCGGAGCCATCGGAGG
CCTCATCGCCATATTTTCACTCTTGATGGGGAACATGGAGGACAACATCAAAGCCACAATGACTCTGACAGCCGGCATCATGTGCGGCATTGCCGG
GGTGTCGGCCTTTGCTAACTTGATCGTGCAAAGCTTTCGGTTCACGACGCCGTACACCTTCGGCCCCGCTCTTTTCGTGGGCTGGATCGGCGGA
GCCGTCCTGGTCATCGGAGGCGTCATGATGTGTCTGGCCTGCCGTGGAATG
cldn18a_tilapia
ATGGCGGCCACATTGTGTCAGGGTTTAGGCTTCGTTCTGAGTTTGATAGGAATAGCGGGAATAATCGCCGCGACGGGGATGGACCAGTGGGCCAC
ACAAGACCTCTTTGACAACGTTGTGACAGCCGTGTACTCGTACTCGGGCCTGTGGAGGTCCTGTGTTAGGCAGAGCTCCGGCTTCACAGAGTGT
CGACCATACTTCACCATCCTCGGCCTGCCAGCTCTGCTCCAAGCCGTCCGAGCCTTGATGATTGTTGGGATTGTCCTTGGCGCTATTGGCTTACTG
ATCGCCATATTCTCGCTGTTGATGGGGAACATGGAGGACAACTTGAAAGCCACCATGACTCTGTCAGCTGGGATCATGTGTGGGATTGCTGGGGT
GTCAGCCTTTGCCAATTTGATTGTACAAAGTTTTCAGTTCACTACACCATATACTTTTGGCCCTGCACTTTTCGTGGGTTGGATCGGTGGCGCTATCT
TGTTCATCGGCGGCATCTTGATGTGCCTGGCCTGCCGTGCAATG
cldn18a_cod
ATGGCGGCCACTCTGTGCCAGGTGATGGGCTTCATCCTGAGCCTGATCGGCGTGGCGGGGATGGTCGTGGCGACCGCAATGGACCCCTGGGCC
ACCCAGGACCTGATGGACAACCCCGTCACCTCCGTCTACATCTTCTCGGGCCTCTGGAAGGCCTGCGTCCGCCAGAGCTCCGGCTTCACCGAGT
GCCGGCCGTACTTCACCATCATGGGGTTGCCAGCGCTGCTGCAGGCGGTGCGAGCGTTGATGATCGTGGGCATTATCCTGGGCGCCATCGGCTG
CCTGGTCGCCATCTTCGCCCTGCTGATGGGCAACATGGAGGACAACATCAAGGCCACCATGACGCTGGCCGCCGGGATCGTGTGTGGGATTGCG
GGCGTGTCGGCCTTCGCTAACCTCATCGTGCAGAGCTTCCGGTTCAACAACACATACACGTTTGGCCCCGCCCTCTTTGTGGGCTGGATAGGGTC
GGCCATCTTGGTTATTGGCGGCGTCCTGATGTGTCTGGCCTGCCGTGGAATG

Supplementary Fig. 12. Claudin 18 nucleotide sequences used to calculate substitution rates. The coding regions were aligned using ClustalW software and sites containing gaps were deleted manually without shifting the reading frame.
