# Supplementary material for: Convergent gene losses and pseudogenizations in multiple lineages of stomachless fishes
Source: Commun Biol. 2024 Apr 3;7:408. doi: 10.1038/s42003-024-06103-x (PMC10991444; doi:10.1038/s42003-024-06103-x)
Supplement: Supplementary file 2 — Reporting summary [file 42003_2024_6103_MOESM2_ESM.pdf]

nature portfolio

Corresponding author(s): Akira Kato

Last updated by author(s): Feb 20, 2024

Reporting Summary

Nature Portfolio wishes to improve the reproducibility of the work that we publish. This form provides structure for consistency and transparency in reporting. For further information on Nature Portfolio policies, see our Editorial Policies and the Editorial Policy Checklist.

Statistics

For all statistical analyses, confirm that the following items are present in the figure legend, table legend, main text, or Methods section.

n/a	Confirmed	
☐	☒	The exact sample size (*n*) for each experimental group/condition, given as a discrete number and unit of measurement
☐	☒	A statement on whether measurements were taken from distinct samples or whether the same sample was measured repeatedly
☐	☒	The statistical test(s) used AND whether they are one- or two-sided<br>*Only common tests should be described solely by name; describe more complex techniques in the Methods section.*
☒	☐	A description of all covariates tested
☒	☐	A description of any assumptions or corrections, such as tests of normality and adjustment for multiple comparisons
☐	☒	A full description of the statistical parameters including central tendency (e.g. means) or other basic estimates (e.g. regression coefficient) AND variation (e.g. standard deviation) or associated estimates of uncertainty (e.g. confidence intervals)
☒	☐	For null hypothesis testing, the test statistic (e.g. *F*, *t*, *r*) with confidence intervals, effect sizes, degrees of freedom and *P* value noted<br>*Give P values as exact values whenever suitable.*
☒	☐	For Bayesian analysis, information on the choice of priors and Markov chain Monte Carlo settings
☒	☐	For hierarchical and complex designs, identification of the appropriate level for tests and full reporting of outcomes
☒	☐	Estimates of effect sizes (e.g. Cohen's *d*, Pearson's *r*), indicating how they were calculated

Our web collection on statistics for biologists contains articles on many of the points above.

Software and code

Policy information about availability of computer code

Data collection	Nucleotide sequence data were downloaded from public database using Google Chrome browser and stored using Microsoft Excel software. Gel images were obtained with CCD camera using Kodak Image Station 2000R system. Tissue images were obtained by TOCO automatic virtual slide system (Path Imaging Inc., Tokyo, Japan) or a microscope equipped with a digital CCD camera (AxioCam HRc; Carl Zeiss, Oberkochen, Germany).
Data analysis	Nucleotide sequence data were analyzed using BLAST analyses using Ensembl and NCBI. Calculation of nucleotide substitution rates were performed using ClustalW and MEGA6 softwares. Gel images were processed with 1D image analysis software (Kodak) and Adobe Photoshop. Tissue images were processed with AxioVision 4.1 software (Carl Zeiss).

For manuscripts utilizing custom algorithms or software that are central to the research but not yet described in published literature, software must be made available to editors and reviewers. We strongly encourage code deposition in a community repository (e.g. GitHub). See the Nature Portfolio guidelines for submitting code & software for further information.

Data

Policy information about availability of data

All manuscripts must include a data availability statement. This statement should provide the following information, where applicable:

- Accession codes, unique identifiers, or web links for publicly available datasets
- A description of any restrictions on data availability
- For clinical datasets or third party data, please ensure that the statement adheres to our policy

All resources are available from the authors upon reasonable request.

Research involving human participants, their data, or biological material

Policy information about studies with human participants or human data. See also policy information about sex, gender (identity/presentation), and sexual orientation and race, ethnicity and racism.

Reporting on sex and gender	No human study was involved in this study.
Reporting on race, ethnicity, or other socially relevant groupings	No human study was involved in this study.
Population characteristics	No human study was involved in this study.
Recruitment	No human study was involved in this study.
Ethics oversight	No human study was involved in this study.

Note that full information on the approval of the study protocol must also be provided in the manuscript.

Field-specific reporting

Please select the one below that is the best fit for your research. If you are not sure, read the appropriate sections before making your selection.

☐ Life sciences ☐ Behavioural & social sciences ☒ Ecological, evolutionary & environmental sciences

For a reference copy of the document with all sections, see nature.com/documents/nr-reporting-summary-flat.pdf

Ecological, evolutionary & environmental sciences study design

All studies must disclose on these points even when the disclosure is negative.

Study description	For nucleotide sequence analyses, we used sequence data from public genome databases. For fish tissue analyses, we used the three-spined stickleback (Gasterosteus aculeatus) as a stomach fish and the humphead wrasse (Cheilinus undulatus) as a stomachless fish.
Research sample	We obtained three-spined stickleback (Gasterosteus aculeatus) and humphead wrasse (Cheilinus undulatus) from a local dealer.
Sampling strategy	The three-spined stickleback humphead wrasse were anesthetized by immersion in 0.1% MS222, which was neutralized to pH 7.4 with sodium bicarbonate prior to use, and then decapitated. The tissues for RNA preparation were removed with ophthalmic scissors and frozen in liquid nitrogen. For histological analyses, the stomach of the stickleback was fixed in 4% paraformaldehyde in 0.1 M phosphate buffer at pH 7.4 for 1 day at 4°C.
Data collection	Synteny analyses were performed by Akira Kato, Chihiro Ota, and Ayumi Nagashima using the public genome databases. RT-PCR was performed by Akira Kato, An-Ping Chen, Zinia Islam, and Naoko Hayashi using RNA extracted from the stickleback tissues. Histological analyses were performed by Akira Kato, Supriya Pipil, Makoto Kusakabe, and Marty Kwok-Shing Wong using stickleback stomach tissue.
Timing and spatial scale	Synteny and phylogenetic analyses were performed once in 2011-2012 and reanalyzed in 2022-2023. RT-PCR was performed on three-spined stickleback in 2012-2014 and on humphead wrasse in 2023. Histological analyses were carried out in 2013-2015.
Data exclusions	No data were excluded from the analyses.
Reproducibility	Nucleotide data analyses were performed with confirmation by two or more persons. RT-PCR and histological analyses were performed twice to confirm reproducibility using the same sample.
Randomization	Since there are not many stickleback and humphead wrasse in circulation and we had only a few chances to get them, we used all

Randomization	healthy fish for the experiments and could not buy fish many times.
Blinding	n/a

Did the study involve field work? ☐ Yes ☒ No

Reporting for specific materials, systems and methods

We require information from authors about some types of materials, experimental systems and methods used in many studies. Here, indicate whether each material, system or method listed is relevant to your study. If you are not sure if a list item applies to your research, read the appropriate section before selecting a response.

Materials & experimental systems

n/a	Involved in the study	
☒	☐	Antibodies
☒	☐	Eukaryotic cell lines
☒	☐	Palaeontology and archaeology
☐	☒	Animals and other organisms
☒	☐	Clinical data
☒	☐	Dual use research of concern
☒	☐	Plants

Methods

n/a	Involved in the study	
☒	☐	ChIP-seq
☒	☐	Flow cytometry
☒	☐	MRI-based neuroimaging

Animals and other research organisms

Policy information about studies involving animals; ARRIVE guidelines recommended for reporting animal research, and Sex and Gender in Research

Laboratory animals	Three-spined stickleback (Gasterosteus aculeatus) and humphead wrasse (Cheilinus undulatus) were obtained from a local dealer.
Wild animals	The three-spined stickleback and humphead wrasse were originally obtained in Japan.
Reporting on sex	Fish tissues other than ovary and testis were once pooled without distinguishing between males and females. Fish ovary and testis were obtained from females and males, respectively, and pooled.
Field-collected samples	We obtained the three-spined stickleback and humphead wrasse from a local dealer.
Ethics oversight	The animal protocols were in accordance with a manual approved by the Institutional Animal Experiment Committee of the Tokyo Institute of Technology.

Note that full information on the approval of the study protocol must also be provided in the manuscript.
